# Multi‑omics identification of a novel signature for serous ovarian carcinoma in the context of 3P medicine and based on twelve programmed cell death patterns: a multi-cohort machine learning study

**DOI:** 10.1186/s10020-024-01036-x

**Published:** 2025-01-08

**Authors:** Lele Ye, Chunhao Long, Binbing Xu, Xuyang Yao, Jiaye Yu, Yunhui Luo, Yuan Xu, Zhuofeng Jiang, Zekai Nian, Yawen Zheng, Yaoyao Cai, Xiangyang Xue, Gangqiang Guo

**Affiliations:** 1 Department of Gynecology, The First Affiliated Hospital, Wenzhou Medical University, Wenzhou, Zhejiang China; 2 School of Medicine, Southern University of Science and Technology, Shenzhen, Guangdong China; 3 First Clinical College, Wenzhou Medical University, Wenzhou, Zhejiang China; 4 Wenzhou Collaborative Innovation Center of Gastrointestinal Cancer in Basic Research and Precision Medicine, Wenzhou Key Laboratory of Cancer-Related Pathogens and Immunity, Department of Microbiology and Immunology, Institute of Molecular Virology and Immunology, Institute of Tropical Medicine, School of Basic Medical Sciences, Wenzhou Medical University, Wenzhou, Zhejiang China; 5 Second Clinical College, Wenzhou Medical University, Wenzhou, Zhejiang China; 6 Cancer Institute (Key Laboratory of Cancer Prevention and Intervention, China National Ministry of Education) of the Second Affiliated Hospital and Institute of Translational Medicine, Zhejiang University School of Medicine, Hangzhou, Zhejiang China; 7 Department of Obstetrics, The First Affiliated Hospital, Wenzhou Medical University, Wenzhou, Zhejiang China

**Keywords:** Serous ovarian carcinoma, Predictive model, Programmed cell death, Cell death index, Predictive preventive and personalized medicine (PPPM/3PM)

## Abstract

**Background:**

Predictive, preventive, and personalized medicine (PPPM/3PM) is a strategy aimed at improving the prognosis of cancer, and programmed cell death (PCD) is increasingly recognized as a potential target in cancer therapy and prognosis. However, a PCD-based predictive model for serous ovarian carcinoma (SOC) is lacking. In the present study, we aimed to establish a cell death index (CDI)–based model using PCD-related genes.

**Methods:**

We included 1254 genes from 12 PCD patterns in our analysis. Differentially expressed genes (DEGs) from the Cancer Genome Atlas (TCGA) and Genotype-Tissue Expression (GTEx) were screened. Subsequently, 14 PCD-related genes were included in the PCD-gene-based CDI model. Genomics, single-cell transcriptomes, bulk transcriptomes, spatial transcriptomes, and clinical information from TCGA-OV, GSE26193, GSE63885, and GSE140082 were collected and analyzed to verify the prediction model.

**Results:**

The CDI was recognized as an independent prognostic risk factor for patients with SOC. Patients with SOC and a high CDI had lower survival rates and poorer prognoses than those with a low CDI. Specific clinical parameters and the CDI were combined to establish a nomogram that accurately assessed patient survival. We used the PCD-genes model to observe differences between high and low CDI groups. The results showed that patients with SOC and a high CDI showed immunosuppression and hardly benefited from immunotherapy; therefore, trametinib_1372 and BMS-754807 may be potential therapeutic agents for these patients.

**Conclusions:**

The CDI-based model, which was established using 14 PCD-related genes, accurately predicted the tumor microenvironment, immunotherapy response, and drug sensitivity of patients with SOC. Thus this model may help improve the diagnostic and therapeutic efficacy of PPPM.

**Supplementary Information:**

The online version contains supplementary material available at 10.1186/s10020-024-01036-x.

## Background

Ovarian cancer (OC) is the most common malignant tumor worldwide, having the third highest mortality rate after breast and cervical cancers (Sung et al. 2021). Nearly 70% of all histopathological types of ovarian carcinomata are serous ovarian carcinomata (SOC), which are characterized by a complex origin and pathogenesis. Patients with SOC are often diagnosed at advanced stages, and fewer than 5% of them have tumors confined to the ovaries (stage I) at diagnosis (Cho and Shih Ie 2009). Furthermore, SOC can be divided into high-grade serous ovarian carcinoma (HGSOC) and low-grade serous ovarian carcinoma (LGSOC) (Cho and Shih Ie 2009). HGSOC, which is associated with chemotherapy resistance, poor prognosis, and frequent recurrence, accounts for 90% of all SOC. Notably, the 10-year mortality of the vast majority of patients is as high as 70% (Deng et al. 2022). Standard treatments, such as tumor reduction surgery, platinum-based chemotherapy, or radiotherapy, are available; however, most patients experience severe side effects or develop chemical resistance and relapse within a few years, making treatment ineffective (Xiao et al. 2022). Thus, a good prediction model may significantly improve the precision of treatments for patients with SOC and help enhance their quality of life. However, effective predictive models for these patients are lacking.

Predictive, preventive, and personalized medicine (PPPM/3PM) is a prominent area of focus in oncology. This approach focuses on targeted prevention measures and personalized treatment strategies, which are important intervention concepts in the shift from cancer biology to precision cancer medicine (Cheng and Zhan 2017; Liu et al. 2023). Furthermore, effective biomolecular markers and predictive models, which may assist physicians in identifying patients at high risk, understanding their cancer characteristics, and providing personalized treatment options, form the basis of PPPM implementation (Lu et al. 2021). However, it important is to acknowledge multi-omics analysis, which identifies precise diagnostic and prognostic markers that may help improve PPPM in these patients.

Programmed cell death (PCD), or regulatory cell death, is the process of autonomous cell death under gene regulation, which differs from accidental cell death. PCD includes necroptosis, pyroptosis, ferroptosis, parthanatos, entosis, NETosis, and lysosome-dependent cell death (Peng et al. 2022a). Abnormal PCD of cancer cells occurs as a result of the occurrence and development of cancer; these cells can escape cell death through PCD-related gene mutation and subsequently proliferate, metastasize, and invade, resulting in adverse consequences. For example, mutations of RAS and TP53 are involved in abnormalities of ferroptosis (Chen et al. 2021a). In general, the downregulation and inhibition of PCD-related pathways are more likely to promote the escape of cancer cells and lead to cancerous behavior. Tong et al. (Tong et al. 2022) summarized the key mechanisms underlying the proliferation and metastasis of cancers associated with the downregulation of PCD-related genes and pathways (necroptosis, pyroptosis, ferroptosis, and cuproptosis). Hence, the first step toward understanding the clinical potential of PCD in cancer is uncovering PCD-related genes and their association with cancer progression.

Dysregulated PCD pathways are potential new targets for cancer therapy. Peng et al. (Peng et al. 2022a) systematically summarized the key pathways associated with PCD, such as tissue necrotic factor-associated ligands and their receptors in apoptosis, the ULK1 complex in autophagy, and the RIPK1/RIPK3/MLKL pathway in necroptosis. Additionally, they suggested many potentially effective interventions. Chen et al. (Chen et al. 2021b) detailed the role of PCD, such as ferroptosis and pyroptosis, in the pathogenesis and clinical treatment of pancreatic cancer and highlighted the potential of activating cell death via precision oncology therapy. Furthermore, an increasing understanding of PCD has led to an increasing number of cancer therapies capable of activating PCD in cancer cells being utilized. More importantly, homologous cancer cells cannot avoid all types of cell death, indicating that susceptible PCD types and drugs may be used to target cancer cell resistance, which provides better treatment outcomes (Raudenská et al. 2021). Thus, gaining a better understanding of PCD will enable researchers to elucidate the degree of malignancy of cancer cells and improve prognoses.

In addition, PCD, which is closely associated with the tumor microenvironment (TME), affects cancer progression. It is caused by the release of intracellular components, including cytokines, adenosine triphosphate, calreticulin, and heat shock proteins, which modulate the TME (Liu et al. 2022). PCD may change the prognosis of patients via the TME. However, it may help improve immune activity, which benefits anti-tumor activity. In contrast, it may lead to an excessive inflammatory response, which promotes tumor development (Du et al. 2021; Liu et al. 2022). In addition, extensive crosstalk takes place between different PCDs. For example, the NLRP3 inflammasome contributes to pyroptosis, apoptosis, necroptosis, and ferroptosis (Huang et al. 2021). Therefore, understanding the expression of PCD-related genes in tumor cells and the tumor immune microenvironment may help enhance the clinical application of PCD.

## Working hypothesis and expected impacts in the framework of predictive, preventive, and personalized medicine

PCD plays an important role in cancer development and in achieving therapeutic effects. To achieve cell proliferation, cancer cells must avoid PCD. Different PCD expression patterns may indicate differences in metastasis risks, invasion tendencies, drug resistance, and immune microenvironments and thus reflect patients' survival rates and time to some extent. Furthermore, there is an urgent need to identify accurate risk and prognosis indicators for patients with SOC. Currently, the study of PCD in SOC remains in its infancy, and its relevance in predicting the prognosis of patients with SOC remains unclear. Therefore, we used various databases to screen survival-related PCD genes and developed a cell death index (CDI) that may be applied to improve the prognosis predictions of patients with SOC and to evaluate their survival time, TME, and drug resistance. As a novel signature, we hope that the developed CDI, may enable patients to choose personalized and precisely targeted treatments within the PPPM framework, leading to improved therapeutic outcomes.

## Methods

### Data collection

We collected 1254 PCD-related genes from previous reports (Tang et al. 2019; Zou et al. 2022), including the 12 PCD patterns mentioned below. In total, 580 apoptosis genes, 52 pyroptosis genes, 88 ferroptosis genes, 367 autophagy genes, 101 necroptosis genes, 14 cuproptosis genes, nine parthanatosis genes, 15 entotic cell death genes, eight NETotic cell death genes, 220 lysosome-dependent cell death genes, seven alkaliptosis genes, and five oxeiptosis genes were identified (Table S1). Finally, these genes were included in different analyses and screened.

The training set included data from 340 patients from GDC TCGA Ovarian Cancer (TCGA-OV). The screening criteria were as follows: (i) survival information; (ii) cancer stage; (iii) clinical diagnosis of SOC; (iv) after surgery; (v) removal of technique duplications from the same patient; and (vi) gene expression data. Gene expression profiles were obtained from the bulk RNA-seq dataset of GDC TCGA-OV provided by UCSC Xena and converted into log_2_(TPM + 1) values. The validation set was divided into two cohorts because of the inconsistent use of platforms. Cohort 1 included GSE26193 (79 cases) and GSE63885 (70 cases), using the GPL570 platform and the Affymetrix Human Genome U133 Plus 2.0 Array. In total, 149 patients with SOC were enrolled in this study. We acquired the CEL files from GEO. The R package affy was used to perform rma to convert the CEL files to an expression measure, and the combat function of the R package sva was used to perform the batch correction. In cohort 2 (GSE140082), 276 patients with a pathological diagnosis of SOC were selected, and normalized expression data from the GEO dataset were used for verification. We further incorporated the GSE53963 cohort to further validate the accuracy of our model (174 cases) for ROC analysis. Single-cell transcriptome sequencing data were obtained from the GEO database (GSE184880 and GSE213243), whereas spatial transcriptome data were acquired from the 10 × genomics database (https://www.10xgenomics.com/resources/datasets/human-ovarian-cancer-11-mm-capture-area-ffpe-2-standard; Table S2). The datasets included in this study were selected based on the following criteria: (1) The platforms used in these datasets must cover a relatively comprehensive range of genes. The number of missing model genes is < 2; (2) The selected datasets should exhibit high sequencing quality and provide comprehensive clinical information. The necessary clinical information are: grade, stage, survival information, and pathological diagnosis; (3) These selected datasets also provide complete raw data, enabling us to perform a unified re-analysis. Moreover, the number of patients who meet the criteria (including those integrated on the same platform) should be > 100; (4) The single-cell dataset requires the use of Chromium Single Cell 3′ V3 Reagent Kits (10 × Genomics) to construct a library that provides complete and accessible sequencing results, with < 20% of low-quality cells. In addition, the dataset needs to include rich and representative cell types and clinical patient information.

### Identification of expression and variation levels of PCD-related genes in SOC

We collected SOC and ovarian transcriptome data from TCGA-OV and GTEx integrated with UCSC Xena, respectively. We used the R package edgeR for variance analysis, standardized methods for the trimmed mean of M-values, and a threshold of | log_2_FC |> 0.5, P < 0.05. We obtained 779 eligible differential genes, and a Circos plot of the differential genes was drawn up using the R package circlize. We used the R package maftools to analyze and visualize somatic mutation data and copy number mutation data from TCGA-OV integrated with UCSC Xena.

### Functional enrichment analysis of PCD-related differentially expressed genes

Kyoto Encyclopedia of Genes and Genomes (KEGG) and Gene Ontology (GO) enrichment analyses, along with Gene Set Variation Analysis (GSVA), were performed using the R package clusterProfiler to confirm differentially expressed genes (DEGs)-related biological functions and differences in biological pathways between the high and low CDI groups. The hallmark used for the GSVA analysis was msigdb v2022.1.

### Construction of survival prediction model for PCD-related genes

We screened 779 DEGs obtained via differential analysis to improve the accuracy of the prediction model. The R package Survminer was used to screen 272 genes, which were highly correlated with survival. Furthermore, we obtained a model of 20 and 14 genes using LASSO regression and stepwise Cox regression, respectively. The model outputs each patient's CDI using the predict.coxph function of the R package Survival. The median number of CDI cases was used to define each dataset's high and low CDI groups. We performed a principal component analysis (PCA) for the cluster discrimination of cells. Subsequently, the R packages Survival and Survminer were used to perform a Kaplan–Meier (KM) analysis to investigate the relationship between CDI and survival. The R package timeROC was used for time-dependent receiver operating characteristic (ROC) analysis to evaluate the effectiveness of the CDI.

### Establishment of nomogram

We summarized the clinicopathological characteristics (age, stage, and grade) of the patients and the CDI, using univariate and multivariate Cox regression to illustrate the potential prognostic significance of the CDI, and constructed a nomogram using the R package regplot. The accuracy and feasibility of the nomogram were evaluated using a calibration curve, decision curve analysis (DCA), and ROC analysis. The R package rms was used to perform Cox regression and plot calibration plots of the data derived from TCGA-OV. The R package ggDCA was used for DCA.

### Functional enrichment analysis between high- and low-CDI subgroups

DEGs between subgroups were analyzed using the R package limma. Pathway and process enrichment analyses were performed using MetaScape software (http://metascape.org/). For the DEGs, pathway and, process enrichment analyses were performed using the following ontology sources: the KEGG Pathway, GO Biological Processes, Reactome Gene Sets, Canonical Pathways, CORUM, WikiPathways, and the PANTHER Pathway. All genes in the genome were used as enriched backgrounds. Terms with a P-value < 0.01, a minimum count of three, and an enrichment factor > 1.5 (the enrichment factor is the ratio between the observed counts and counts expected by chance) were collected and grouped into clusters based on their membership similarities. Specifically, p-values were calculated based on the cumulative hypergeometric distribution, and q-values were calculated using the Benjamini –Hochberg procedure to account for multiple tests. Kappa scores were used as the similarity metric when performing hierarchical clustering on the enriched terms, and subtrees with a similarity of > 0.3 were considered a cluster. The most statistically significant term within a cluster was selected to represent the cluster.

Gene Set Enrichment Analysis (GSEA) was performed using the R package GSVA; GSVA, which allowed for the unsupervised estimation of pathway activity variation across a sample population. Gene sets, including "c2.cp.kegg.v2023.1. Hs.symbols.gmt" and "c2.cp.reactome.v2023.1. Hs.symbols.gmt" were downloaded from MSigDB (https://www.gsea-msigdb.org/gsea/msigdb).

### Single-cell RNA-seq dataset analysis

Single-cell transcriptome sequencing datasets of SOC from the GEO database were used to validate the predictive model further. The count matrices were merged, integrated, and processed with the R package Seurat. For quality control, we removed cells with mitochondrial gene content over 25% and the number of genes fewer than 500 or over 6000. Subsequently, we performed data normalization via the NormalizeData function with standard parameters. The processed expression matrix was scaled using the ScaleData function. Furthermore, we identified highly variable features using the FindVariableFeatures function. Next, PCA was performed using the RunPCA function based on the 3000 highly variable genes detected in the assay of variable features. Additionally, T-distributed stochastic neighbor embedding (tSNE) dimensionality reduction analysis was performed using the RunTSNE function with the top 30 principal components as input to visualize the dataset.

Next, the whole dataset and the myeloid subset were clustered with the resolution set at 0.5. Cell types were annotated based on the expression of typical markers and the predict.coxph function of the Survival package was used to calculate the CDI for each cell. We used the progeny function in the R package progeny to estimate the activity of multiple pathways to compare high-CDI cell clusters with low-CDI cell clusters at the single-cell level. Moreover, DEGs were identified using the FindMarkers and FindAllMarkers functions in the Seurat package with adjusted p-values set at < 0.05. GSEA was performed using the R package clusterProfiler. Hallmark and metastatic gene sets were obtained from MSigDB v2022.1 for this analysis. Additionally, macrophage M1 and M2 signatures were obtained from the study by Sun et al. (Sun et al. 2022). We explored the differences in intercellular communication between the high and low CDI clusters using the CellChat package.

### Immune cell abundance analysis in TME

We calculated the Pearson correlation between the immune checkpoint genes and CDI based on bulk RNA-seq and the gene expression profiles of the arrays to explore the predictive model for the abundance analysis of tumor-infiltrating immune cells further. The compositional differences of 22 tumor-infiltrating immune cells were evaluated by scoring with the R package CIBERSORT, and the results were visualized using box plots. Furthermore, we compared the ratio of M2/M1 macrophages among the three cohorts and calculated Pearson's correlation of the CDI. To verify the relationship between macrophage polarization and the CDI, macrophages were distinguished from myeloid cells in GSE184880 based on the expression of marker genes and subsequently classified into different clusters based on the level of CDI values.

### Spatial transcriptome data processing and analysis

The filtered feature-barcode matrix and spatial imaging data were downloaded from the 10 × genomics database. In brief, the spatial datasets were imported, and further analysis was performed using the Seurat package. Next, data were normalized and scaled using SCTransform with standard parameters. Hereafter, PCA was performed using the RunPCA function. To visualize and explore the dataset, tSNE dimensionality reduction analysis was performed using the RunTSNE function with the top 30 principal components as input. Subsequently, data were clustered (FindClusters) with the resolution set at 0.5. This was followed by using the spacexr package was to deconvolute spatial transcriptomic spots into cell types (Cable et al. 2022). Mapping was performed using creat.RCTD and run.RCTD functions with a full pattern to deconvolute the data from Visium spots into underlying cell types, predicting the proportion of each cell type in each spot. The GSE184880 dataset was used as a reference scRNA-seq dataset (Xu et al. 2022). The predict.coxph function of the R package Survival was used to calculate the CDI for each spot. We calculated the log-scaled CXCL9/SPP1 (CS) ratio using the normalized expression profile to identify the macrophages detrimental to prognosis (Bill et al. 2023). DEGs were identified using the FindMarkers function, and GO enrichment analysis was performed using the R package clusterProfiler.

### Mutational analysis

We obtained neoantigens and mutation loads for SOC from the Cancer Immunome Atlas database (https://tcia.at/). The mutation landscape was visualized using maftools (R package). Differentially mutated genes (DMGs) were analyzed via the mafCompare function.

### Prediction of immunotherapy and drug sensitivity

The TCGA-OV cohort gene expression profiles was imported into the Tumor Immune Dysfunction and Exclusion (TIDE, http://tide.dfci.harvard.edu) network platform to predict the immunotherapy response. The R package oncoPredict was used to predict the half-maximal inhibitory concentration (IC50) for chemotherapy response in the TCGA-OV cohort.

### Statistical analysis

All statistical analyses were conducted using R software (v.4.2.2). Student's *t*-test or the Wilcoxon test was used to compare data between low and high CDI groups in RNA-Seq datasets. The significance of the statistical differences between low and high CDI clusters in single-cell transcriptome sequencing datasets was analyzed using the MAST test. The log-rank test compared the survival curves between low and high CDI groups. Statistical significance was set at P < 0.05.

## Results

### Workflow and methods

**Fig. 1:**
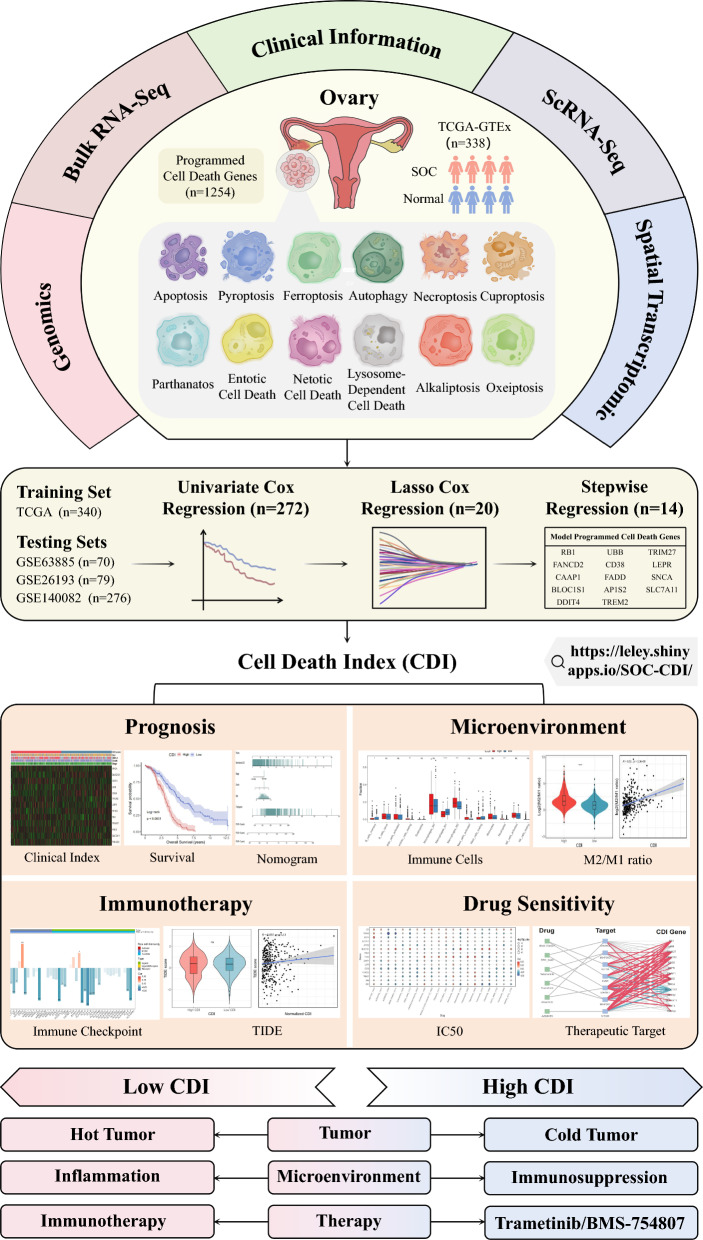
Flowchart for comprehensive analysis of diverse PCD patterns in patients with SOC

The workflow is illustrated in Fig. 1. For the training cohort, we identified 340 patients with SOC from the TCGA; for validation cohorts, we identified 79 patients from GSE26193, 70 patients from GSE63885, and 276 patients from GSE140082. Furthermore, we collected 1254 PCD-related genes from previous reports (Tang et al. 2019; Zou et al. 2022) to select the genes needed to build the model. In addition, we conducted gene mutation analysis in 338 patient samples from TCGA-GTEx and ultimately identified 779 differentially expressed PCD-related genes. For the single-cell RNA transcription dataset, we collected seven and one OC samples from GSE184880 (Xu et al. 2022) and the scRNA-seq library of Ren et al. (Ren et al. 2022), respectively. Finally, we screened 14 PCD genes, including 5/12 cell death patterns, via the nomogram model to predict patient prognosis. In addition, we analyzed the association between the CDI and the TME, drug sensitivity, and single-cell sequencing results.

### Different expression analysis of PCD-related genes in patients with SOC

**Fig. 2:**
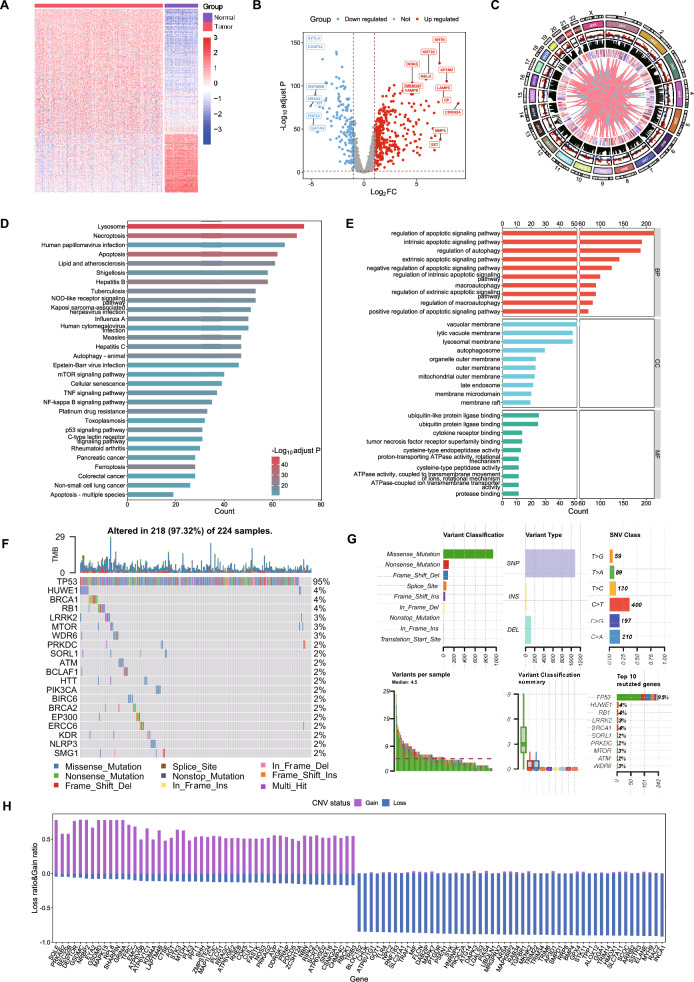
Variant landscape of PCD-related genes in patients with SOC. **A** Heatmap of PCD-related differentially expressed genes (DEGs) between SOC and normal tissues. **B** Volcano plot of PCD-related DEGs. Points with labels were part of the obvious DEGs with an adjusted P-value < 0.001 and |log_2_FC|> 4. **C** Circos plot of location, fold-change (FC), expression level, and significant correlation with the PCD-related DEGs. **D** Kyoto Encyclopedia of Genes and Genomes (KEGG) enrichment analysis of DEGs. **E** Gene Ontology (GO) enrichment analysis of the DEGs. **F** Oncoplot of the top 20 most frequently mutated PCD-related genes in the TCGA-OV cohort. **G** Summary of somatic mutations in the PCD-related genes in the TCGA-OV cohort. **H** Top 100 PCD-related genes with the most significant copy number variations

We identified PCD-related DEGs in the TCGA-GTEx cohort and analyzed their functions to explore the potential impact of PCD on SOC. First, we observed the variation landscape of DEGs in SOC tissues, identifying 526 upregulated and 253 downregulated genes. We used a heatmap to detect the RNA expression of PCD-related DEGs, with red and blue representing upregulated and downregulated expression levels, respectively (Fig. 2Fig. 2B). Additionally, we analyzed the chromosomal locations, changes in expression, and correlations of the PCD-related DEGs (Fig. 2C). In addition, KEGG enrichment analysis showed that the most notable pathways with enriched DEGs were mainly associated with cell death regulation, including lysosome and necroptosis, with a high number of enriched genes and significant p-values (Fig. 2D). The GO analysis confirmed these findings, indicating that the DEGs were significantly concentrated in the regulation of the apoptotic signaling pathway, the intrinsic apoptotic signaling pathway, and autophagy (Fig. 2E). We also specifically analyzed PCD-related gene variations. The results showed that 97.32% (218/224) of the patients with SOC had mutations; the top 20 mutations of PCD-related genes were displayed, of which TP53 was the gene with the highest frequency of mutations (95%), and the remaining 19 genes had mutation frequencies ranging from 2–4%; missense mutation was the main variant classification, and single nucleotide polymorphism was the main variant type (Fig. 2F and G). The copy number variation (CNV) status analysis results showed that PCD-related genes in patients with SOC had frequent copy number changes, which may help cancer cells escape PCD (Fig. 2H). The above results show the variation landscape of the PCD-related genes in patients with SOC, reveal the biological functions of PCD-related DEGs in SOC processes, and suggest a potential link between PCD and SOC.

### Construction and validation of predictive model based on PCD-related genes

**Fig. 3:**
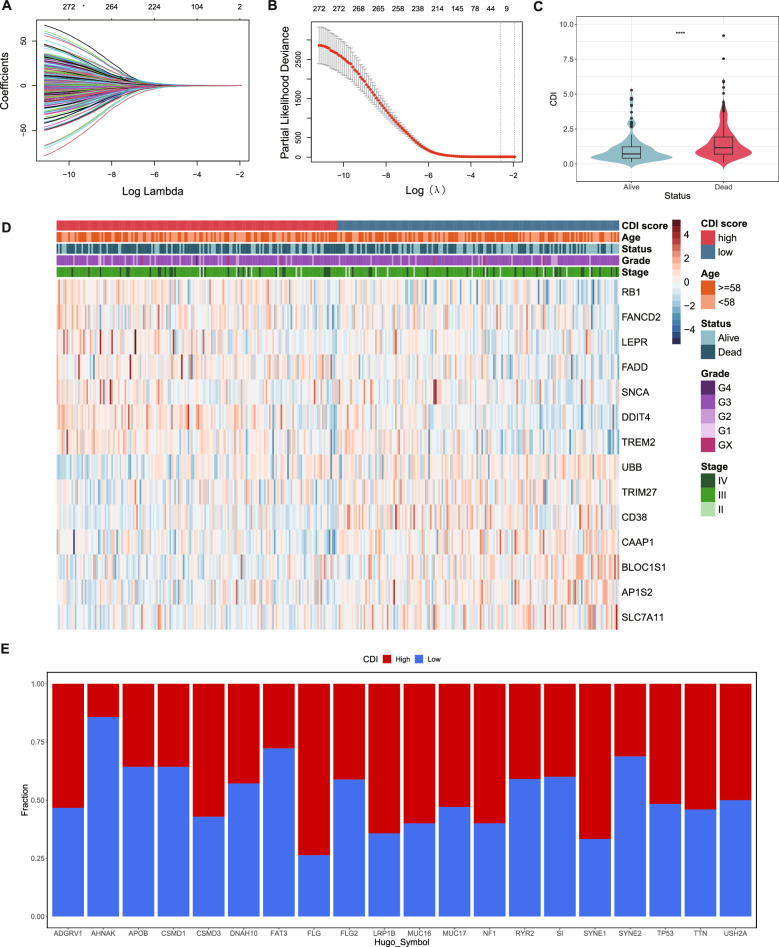
Construction of the PCD-related gene signature for patients with SOC. **A** LASSO coefficient profiles of 272 PCD genes. **B** Cross-validation of the gene signature. **C** Violin plot showing the relationship between the cell death index (CDI) and survival status. **D** Heatmap of the clinical features and model gene expression in the TCGA-OV cohort. The age cutoff was the median patient age. **E** The mutation frequency of the top 20 mutable genes between high and low-CDI groups in SOC patients in TCGA-OV

We used Lasso regression and Cox analysis to screen the PCD-related DEGs further and build a prognosis gene model to explore the impact of PCD on the prognosis of patients with SOC and to detect the best survival-related genes. We identified the best punishment coefficient among the 272 highly survival-related genes (lambda.min = 0.07488). We narrowed the gene range according to the coefficient, eventually retaining 14 genes (*RB1, UBB, TRIM27, FANCD2, CD38, LEPR, CAAP1, FADD, SNCA, BLOC1S1, AP1S2, SLC7A11, DDIT4, and TREM2*) for predictive model construction, six being derived from apoptosis, four from autophagy, two from ferroptosis, one from necroptosis, and two from lysosome-dependent cell death (FADD belongs to both apoptosis and necroptosis) (Fig. 3A and B). Pearson's correlation analysis showed the correlation between each model gene (Fig. S1), and KM analysis showed the influence of the expression level of each model gene on the survival rate of patients (Fig. S2). The Wilcoxon test was used to compare the expression differences of each model gene between SOC and normal tissues (Fig. S3A). After testing the effectiveness of the included genes, we calculated the CDI using the following formula:

$$CDIscore = 0.16220*RB1{\mkern 1mu} \,expression - 0.17038{\mkern 1mu} *{\mkern 1mu} UBB{\mkern 1mu} \,\,expression{\mkern 1mu} - {\mkern 1mu} 0.41386{\mkern 1mu} *{\mkern 1mu} TRIM27{\mkern 1mu} \,\,expression{\mkern 1mu} + {\mkern 1mu} 0.26017{\mkern 1mu} *{\mkern 1mu} FANCD2{\mkern 1mu} \,\,expression{\mkern 1mu} + {\mkern 1mu} 0.29119{\mkern 1mu} *{\mkern 1mu} CD38\,\,expression + {\mkern 1mu} 0.25412{\mkern 1mu} *{\mkern 1mu} LEP\,{\text{Re}}xpression - {\mkern 1mu} 0.40824{\mkern 1mu} *{\mkern 1mu} CAAP1{\mkern 1mu} \,\,expression{\mkern 1mu} + {\mkern 1mu} 0.43810{\mkern 1mu} *{\mkern 1mu} FADD\,\,expression + {\mkern 1mu} 0.20286{\mkern 1mu} *{\mkern 1mu} SNCA\,\,expression{\mkern 1mu} - {\mkern 1mu} 0.22865{\mkern 1mu} *{\mkern 1mu} BLOC1S1\,\,expression - {\mkern 1mu} 0.26146{\mkern 1mu} *{\mkern 1mu} AP1S2\,\,expression{\mkern 1mu} - {\mkern 1mu} 0.27573{\mkern 1mu} *{\mkern 1mu} SLC7A11{\mkern 1mu} \,\,expression{\mkern 1mu} + {\mkern 1mu} 0.12693{\mkern 1mu} *{\mkern 1mu} DDIT4{\mkern 1mu} \,\,expression{\mkern 1mu} + {\mkern 1mu} 0.09397{\mkern 1mu} *{\mkern 1mu} TREM2{\mkern 1mu} \,\,expression{\mkern 1mu}$$

Next, we performed multiple analyses to reveal the relationship between these 14 genes and SOC. We used the TCGA cohort as the training set, calculated the CDI of each patient with SOC, and divided the patients into high and low CDI groups with the median as the boundary. Furthermore, we generated a violin diagram that indicated that the CDI of patients in the death group was significantly higher than that in the survival group to confirm the relationship between the CDI and the survival status of patients. Therefore, suggesting that a high CDI indicates a poor prognosis (Fig. 3C). The heat map further demonstrates the relationship between the CDI and age, survival status, grade, stage, and 14 model genes from the perspective of clinical characteristics. The CDI was significantly correlated with the survival status of the patients; the mortality of patients in the high CDI group was significantly higher than that of the patients in the low CDI group (Fig. 3D). However, the CDI was not associated with patient’s stage, grade, or age (Fig. S3B). These results suggested that CDI may be an independent factor in the prognosis of patients with SOC.

Next, we explored the relationship between CDI and prognosis from the perspective of genetics. The stacked bar chart shows the differences between the top 20 mutated genes of the high and low CDI groups (Fig. 3E). The chart showed that the mutation rate of AHNAK was higher in the low CDI group than in the high CDI group, indicating that the low CDI group has a better prognosis (Ghodke et al. 2021) and that immunotherapy may exert a better effect on the low CDI group (Zhao et al. 2021b). These results indicated that a higher CDI score may associated with a worse prognosis. We successfully constructed a CDI related to patient prognosis and preliminarily validated the predicted model.

### Internal training and external validation of prognostic implications of CDI

**Fig. 4:**
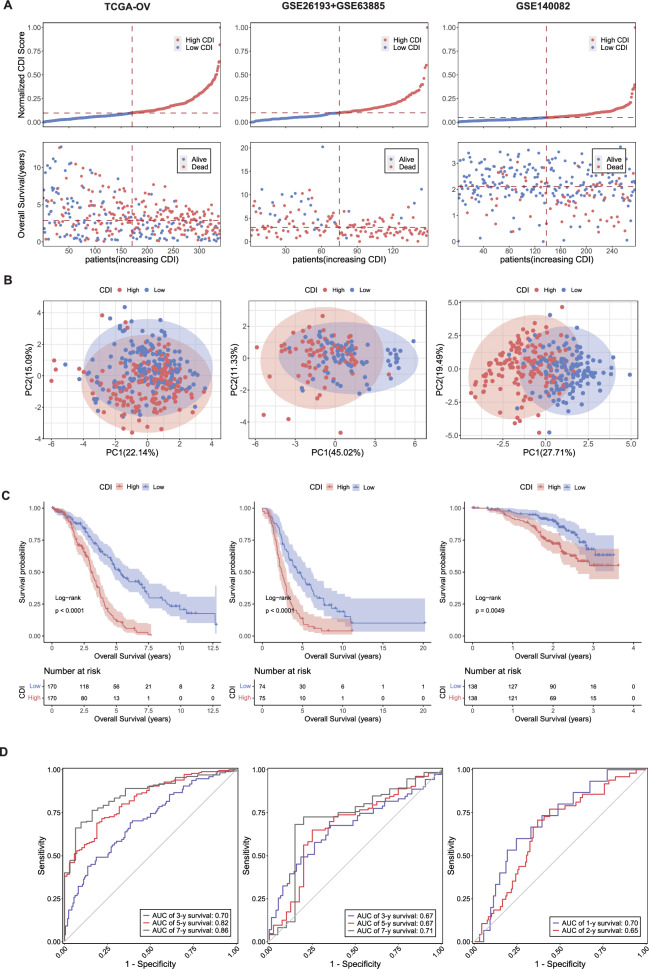
Prediction model performance evaluation. **A** Distribution of survival status and time according to the normalized CDI in the TCGA-OV, GSE63885 + GSE26193, and GSE140082 cohorts. Dashed lines represent the dividing lines of the median number. **B** The principal component analysis plot based on the CDI in the TCGA-OV, GSE63885 + GSE26193, and GSE140082 cohorts. **C** Kaplan–Meier (KM) survival curve for the overall survival (OS) of the low and high CDI group patients in the TCGA-OV, GSE63885 + GSE26193, and GSE140082 cohorts. **D** Receiver operating characteristic (ROC) analysis of the model in the TCGA-OV, GSE63885 + GSE26193, and GSE140082 cohorts (Only the 3-, 5-, and 7-year survival is shown, and the rest is detailed in supplementary materials)

We conducted internal training and external validation using the three cohorts, TCGA-OV, GSE63885 + GSE26193, and GSE140082. First, we compared the differences between the survival statuses of the high and low CDI groups, which showed significantly lower overall survival (OS) in the patients with a high CDI than in those with a low CDI (Fig. 4A). The PCA results also confirmed significant differences between patients with high and low CDIs, suggesting that CDI may be a good indicator of differentiation (Fig. 4B). Next, we described the relationship between the patient CDI and survival rate using KM analysis, which also showed that the patients in the high CDI group had a shorter OS in general, and that, for the same OS, the survival rate of patients in the high CDI group was significantly lower than that of the patients in the low CDI group (Fig. 4C). Additionally, we used a ROC analysis to validate the sensitivity and specificity of the predictive model to facilitate clinical applications. In the TCGA-OV and GSE26193 + GSE63885, we selected 1 to 9-year survival to validate prognostic performance of the model, and in GSE140082, limited by sample follow-up time, we selected 1 and 2-year survival time points to validate the model's performance (Fig. 4D and Fig. S4A). Our results showed that CDI had the best performance in predicting training cohort survival, as well as competent performance in the validation cohorts. Besides, we further selected the GSE53963 cohort and performed ROC analysis; the results further showed that our model has good predictive performance and reliability (Fig. S4B). To better show the predictive ability of our model, we have also included a comparison of our model with other published signatures, which also illustrates our model has robust predictive ability and performs better than many published biomarkers (Table S3). In all, these results indicated that CDI shows important prognostic significance for patients with SOC and that patients with a high CDI tend to have a worse OS, suggesting that the CDI may be closely associated with other tumor characteristics.

### Establishment and assessment of the nomogram survival model

**Fig. 5:**
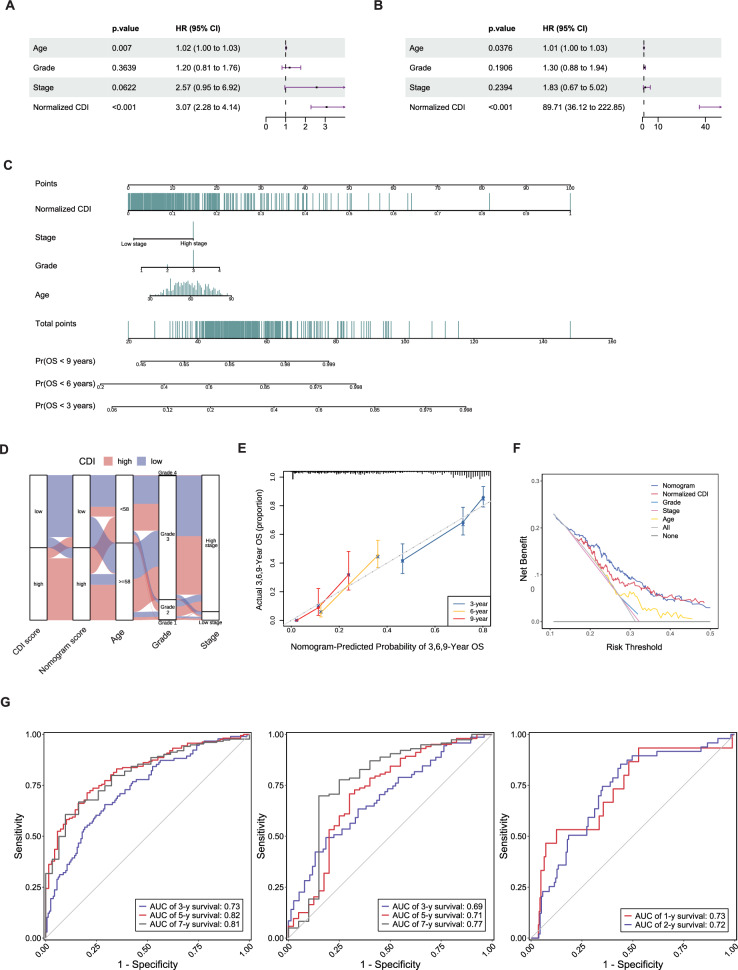
Establishment and performance evaluation of nomogram model. **A** Univariate Cox hazard regression for the clinicopathologic characteristics and normalized CDI in the TCGA-OV cohort. **B** Multivariate Cox hazard regression for the clinicopathologic characteristics and normalized CDI in the TCGA-OV cohort. **C** Nomogram predicting the 3-, 6-, and 9-year OS of patients with SOC. **D** An alluvial diagram shows the interrelationship between the grade, stage, nomogram, and CDI groups in patients with SOC. **E** Calibration plots for 3-, 6-, and 9-year OS probabilities in the TCGA-OV cohort. **F** Decision curve analysis for predicting the OS. **G** ROC analysis of the nomogram in the TCGA-OV, GSE63885 + GSE26193, and GSE140082 cohorts (only the 3-, 5-, and 7-year survival is shown, and the rest is detailed in supplementary materials)

We performed univariate and multivariate Cox regression analyses on the training set to clarify whether the CDI is an independent prognostic indicator of SOC. Univariate Cox regression analysis indicated that the CDI was significantly associated with OS in patients with SOC (HR = 3.07, 95%: 2.28–4.14, P < 0.05; Fig. 5A, Fig. S5A). Following the removal of other confounding factors, multivariate analysis indicated that the CDI was an independent prognostic predictor of SOC (HR = 89.71, 95% CI 36.12–222.85, P < 0.05; Fig. 5B). Univariate and multivariate Cox regression analyses combining the CDI and clinical parameters were also performed for the GSE63885 + GSE26193 and GSE140082 cohorts and the results were consistent with the TCGA-OV cohort (Figs. S5B and S5C). Based on this result, we constructed a nomogram including clinical parameters (age, stage, and grade) and the CDI to estimate 3-, 6-, and 9- year OS (Fig. 5C). Interrelationships among the nomogram score, age, stage, grade, and CDI score of patients are presented in the alluvial diagram (Fig. 5D). Calibration curves revealed that the nomogram-predicted OS was consistent with the observed OS (Fig. 5E). DCA indicated that the nomogram could accurately predict OS and was effective in clinical practice, outperforming other clinical factors (Fig. 5F). In addition, we further verified the accuracy of the nomogram via ROC analysis results of TCGA-OV, GSE63885 + GSE26193, GSE140082, and GSE53963, which showed that nomogram comprehensively considered more prognostic factor and was a good supplement to the prognostic accuracy of CDI (Fig. 5G; Fig. S4C; Fig. S4D). Overall, these results demonstrated from multiple aspects that the nomogram model showed good ability and reliability for OS prediction in patients with SOC; the predictive model we constructed may be obtained from https://leley.shinyapps.io/SOC-CDI/.

### Functional enrichment analysis of programmed cell death-genes model

**Fig. 6:**
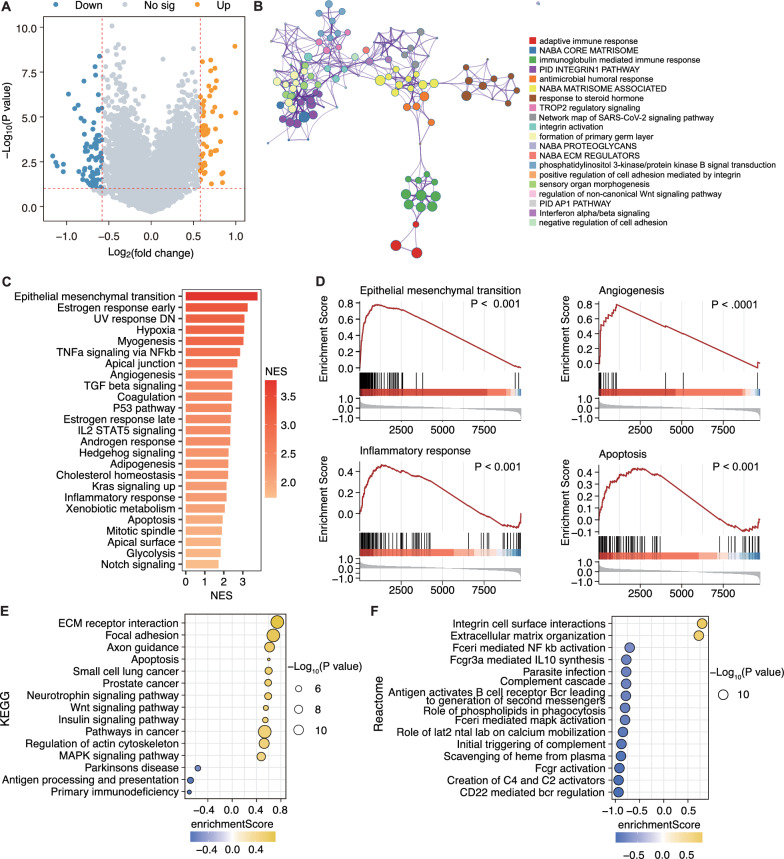
Functional enrichment analysis of PCD genes model between low- and high-CDI patients with SOC. **A** Genes differentially expressed between the high- and low-CDI patients with SOC. Yellow: upregulated genes in the high CDI group; blue: upregulated genes in the low CDI group; **B** Network of the top 20 clusters with their representative enriched terms (one per cluster): colored by cluster ID, where nodes that share the same cluster-ID are typically close to each other. **C** Gene Set Enrichment Analysis (GSEA) enrichment plots for the DEGs; the top 25 pathways are displayed based on NES values. **D** GSEA of the epithelial-mesenchymal transition, angiogenesis, inflammatory response, and apoptosis. **E** KEGG analysis of genes in patients with SOC with high and low CDI. **F** Reactome pathway analysis of genes in patients with SOC with high and low CDI

We performed a functional enrichment analysis between the high and low CDI groups to elucidate the underlying molecular mechanisms of this PCD-genes model. DEGs between the high and low CDI groups were identified using a volcano plot (fold change > 1.5, P < 0.05; Fig. 6A). The functions of the DEGs were identified via GO and GSEA and further verified using KEGG and Reactome pathway analyses. The cluster-enrichment term network suggested that the DEGs were mainly involved in immune and cancer-related pathways, such as adaptive immune response, immunoglobulin-mediated immune response, NABA ECM REGULATORS, and interferon alpha/beta signaling (Fig. 6B). Additionally, the GSEA analysis revealed that the enriched pathways of the high CDI group included epithelial-mesenchymal transition (EMT), angiogenesis, inflammatory response, and apoptosis, which were largely associated with inflammatory responses, cell invasion, and migration (Fig. 6C and D). Furthermore, KEGG and Reactome pathway analyses revealed that the significantly enriched pathways in the high CDI group were apoptosis, the Wnt signaling pathway, pathways in cancer, the mitogen-activated protein kinase (MAPK) signaling pathway, and extracellular matrix organization, suggesting that the differential expression of these DEGs may be associated with changes in the tumor immune microenvironment (Fig. 6E and F). These results provide evidence that the aberrant expression of PCD genes may be involved in cancer development and illustrate cancer-immunity interaction as a potential mechanism of the PCD-genes model, prompting that relevant studies of this model in TME and immunotherapy are needed.

### scRNA-seq showed robust tumor signaling pathway activity in tumor cell clusters with high CDI

**Fig. 7:**
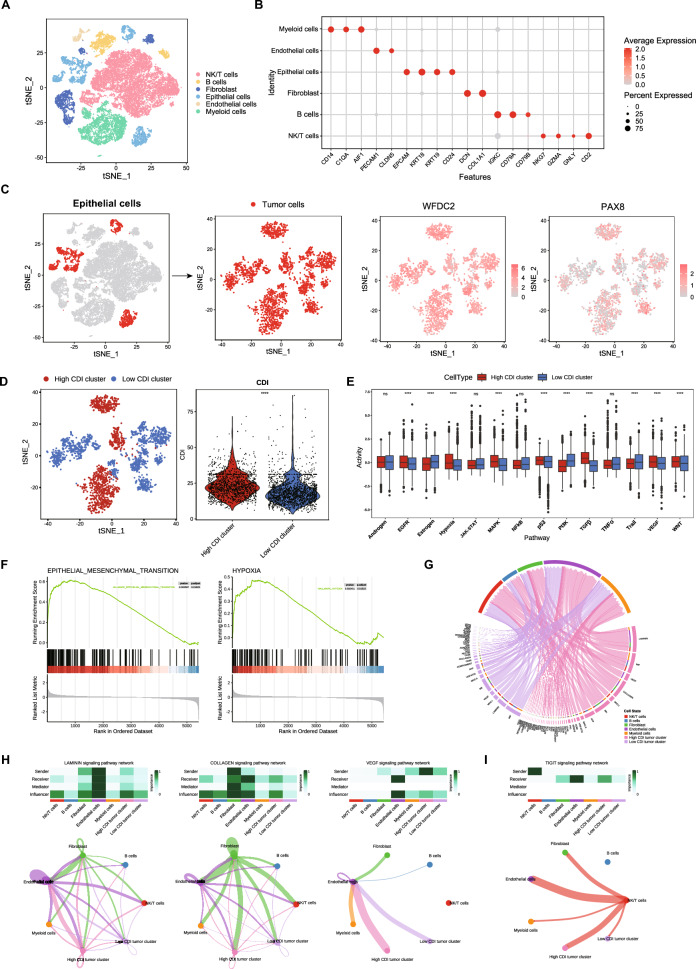
Single-cell transcriptome analysis reveals an association between CDI and malignant tumor cells. **A** t-distributed stochastic neighbor embedding (tSNE) visualization of the diverse cell types in tumor samples from GSE184880. **B** Bubble plots of cell-type marker gene expression levels. **C** tSNE visualization of the tumor cells and WFDC2 and PAX8 expression. **D** tSNE and violin plots showing the high and low CDI tumor groups and the CDI values of the tumor cells. **E** Box plot of scaled scores of the cancer-relevant pathways in the tumor cells. **F** GSEA of the hallmark epithelial-mesenchymal transition gene set and hypoxia. **G** Chord diagram of the signaling pathways from the high and low CDI tumor groups to other cells. **H** Heatmaps and Circos plots of the LAMININ, COLLAGEN, and VEGF signaling pathway networks. **I** Heatmaps and Circos plots of the TIGIT signaling pathway networks

We analyzed the single-cell RNA transcriptome data from patients with SOC to explore SOC's microenvironment and immune status. We annotated different cell clusters, including NK/T cells, B cells, fibroblasts, epithelial cells, endothelial cells, and myeloid cells (Fig. 7A). The bubble plot showed the expression levels of cell-type marker genes, including myeloid cells (characterized by CD14, C1Q4, AIF1), endothelial cells (PECAM1, CLDN5), epithelial cells (EPCAM, KRT18, KRT19, COL1A1), fibroblasts (DCN, THY1), B cells (IGKC, CD79A, CD79B), and NK/T cells (CD2) (Fig. 7B). Furthermore, we used tSNE to visualize tumor cells and the expression levels of WFDC2 and PAX8 (Fig. 7C). Notably, WFDC2 is widely used as a biomarker in OC, and PAX8 is an essential histological marker in a majority of epithelial OCs, as they are highly expressed in most malignant OCs (Gokulnath et al. 2021; James et al. 2022). Based on the CDI value, the tumor cells were classified into high and low CDI clusters with a relatively clear resolution (Fig. 7D). As shown in Fig. 7E, we compared the scaled score of normalized activators of cancer-related pathways in the two CDI clusters, which showed that 10 activators varied significantly and that pathways such as epidermal growth factor receptor (EGFR), hypoxia, MAPK, transforming growth factor beta (TGF-β), and vascular endothelial growth factor (VEGF) were upregulated in the high CDI clusters. The EGFR, hypoxia, MAPK, p53, phosphoinositide 3-kinase (PI3K), TGF-β, VEGF, and Wnt pathways are involved in the progression, invasion, metastasis, or drug resistance of OC (Basu et al. 2015; Belur Nagaraj et al. 2021; Chen et al. 2018; Dorayappan et al. 2018; Huang et al. 2020; Wang et al. 2023; Zhao et al. 2021a).

Furthermore, GSEA revealed that the hallmark EMT and hypoxia gene sets were enriched in the cells of high CDI clusters (Fig. 7F). Additionally, we investigated the interactions between tumor cells in the high and low CDI groups and other types of cells in the TME. The chord diagram shows high and low CDI clusters with different cell signaling pathways (Fig. 7G). In the LAMININ, COLLAGEN, and VEGF signaling pathway networks, tumor cells with a high CDI produced more signaling associations with endothelial cells and fibroblasts (Fig. 7H), which may facilitate the migration and metastasis of tumor cells and angiogenesis (Carmeliet 2005; Hao et al. 2022; Song et al. 2022). In the TIGIT signaling pathway network (TIGIT-NECTIN2 axis), tumor cells with a high CDI interacted with NK/T cells more significantly, which was conducive to tumor immunosuppression (Fig. 7I) (Ho et al. 2021; Sim et al. 2022). The above results illustrated that tumor cells with a high CDI in patients with SOC are more inclined toward invasion, metastasis, and angiogenesis and have stronger tumor signaling pathway activities.

We performed scRNA-seq on another cohort, GSE213243, and obtained consistent results (Fig. S6). The high CDI group was correlated with the androgen, EGFR, estrogen, hypoxia, JAK-STAT, MAPK, NF-kB, PI3K, TGF-α, Trail, and VEGF pathways (Fig. S6E) and contained enriched EMT and hypoxia gene sets (Fig. S6F). Expression of the cancer-associated metastasis gene set in high CDI clusters was significantly increased, indicating that patients with SOC with a high CDI may be more prone to cancer metastasis (Fig. S6G). In addition, we explored the relationship between the tumor cells and model genes (Fig. S7). Violin plots showed the magnitude of the CDI values for different cell types, and the CDI was highly composed of epithelial cells (Fig. S7A). The bubble plot showed that different CDI model genes may correspond to different components in the TME, such as TREM2, mainly to myeloid cells, and CD38 to B cells (Fig. S7B). Moreover, we separately calculated the scores of tumor cells in single-cell transcriptome data using different death-type genes and corresponding parameters from the model, which showed that the scores from the apoptosis gene set and the lysosome-dependent cell death gene set differed most significantly between the two groups, suggesting that genes with these two models dominate the CDI division of tumor cells (Fig. S7C). Furthermore, differential analysis of tumor cells in the two single-cell datasets (high CDI vs. low CDI) found that all genes from the lysosome-dependent cell death gene set (BLOC1S1, AP1S2) were stable in both datasets (Fig. S7D).

### Dissection of tumor microenvironment based on programmed-cell death signature

**Fig. 8:**
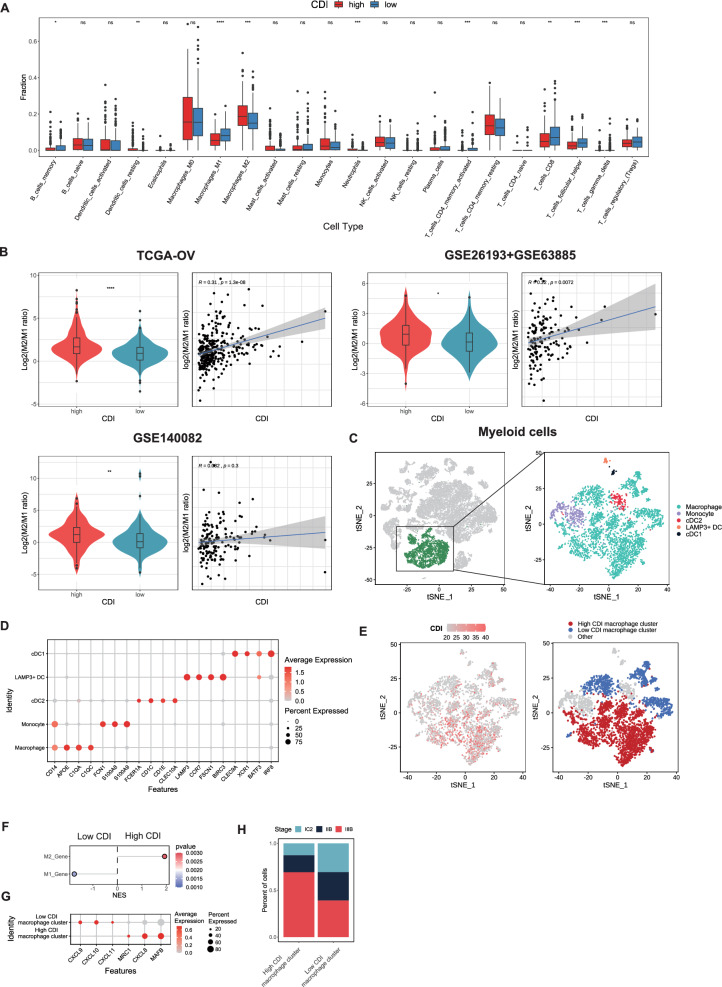
Dissection of tumor microenvironment (TME) based on PCD signature. **A** Box plots of the proportions of 22 types of immune cells between the high and low CDI groups, as predicted by CIBERSORT. **B** Violin plots of the log-homogenized M2/M1 macrophage ratio (M2/M1 ratio) between the high and low CDI groups in the TCGA-OV, GSE63885 + GSE26193, and GSE140082 cohorts. **C** tSNE visualization of myeloid cells from five patients with SOC in the GSE184880 cohort. **D** Bubble plot of the myeloid subset marker gene expression. **E** tSNE visualization of the CDI cluster group of macrophages. **F** Lollipop plot of the GSEA results for macrophage M1 and M2 scoring. **G** Bubble plot of the differentially expressed genes between the high and low CDI clusters of macrophages. **H** Bar plots showing that macrophages in the high CDI cluster are more likely to originate from high-stage patients

PCD may influence cancer progression via the TME. Hence, we conducted landscaping of the TME using the predictive model. We attempted to identify 22 tumor immune cell infiltration landscape using CIBERSORT to determine whether the proportion of immune cells varied between the high and low CDI groups. The TCGA-OV results revealed that M2 macrophages and neutrophils were significantly higher in the TME of the high CDI group than in the low CDI group. In comparison, M1 macrophages, activated memory CD4^+^ T cells, Tfh cells, and γδT cells were higher in the low CDI group (Fig. 8A). GSE63885 + GSE26193 and GSE140082 also revealed a higher proportion of M1 macrophages in the low CDI group than in the high CDI group (Figs. S8A and S8B). We found that in these three datasets, the high CDI group had a higher M2/M1 macrophage ratio (M2/M1 ratio), and the correlation analysis showed that the M2/M1 ratio is positively correlated with CDI (Fig. 8B).

We performed further analyses using myeloid cells from scRNA-seq data (Sun et al. 2022). We showed the main constituent myeloid cells and their marker genes' expression levels (Fig. 8C and D). Next, tSNE analysis was conducted to classify the macrophages into high and low CDI clusters based on their CDI values (Fig. 8E). Subsequently, GSEA revealed that the high CDI group had more significant M2 macrophage characteristics than the low CDI group (Fig. 8F). Meanwhile, we found that secretion of factors associated with anti-inflammatory (MRC1, CXCL8, and MAFB) (Cambier et al. 2023; Kim 2017) in the high CDI group was significantly enhanced, while CXCL9, CXCL10, and CXCL11 expression in the low CDI group was increased (enhanced paracrine CXCL9, CXCL10, and CXCL11 expression indicates anti-tumor activity (Tokunaga et al. 2018); (Fig. 8G). Regarding the relationship between CDI and the tumor stage, we found that myeloid cells with a high CDI were mainly from patients with an advanced stage of cancer. Further, we observed that tumors mostly metastasized to the extra-pelvic involvement of the peritoneum (IIIb) (Fig. 8H). The high CDI group formed a TME dominated by M2 macrophage infiltration, whereas the low CDI group exhibited M1 macrophage infiltration.

### Colocalization of CDI and malignant markers identified by spatial transcriptomics

**Fig. 9:**
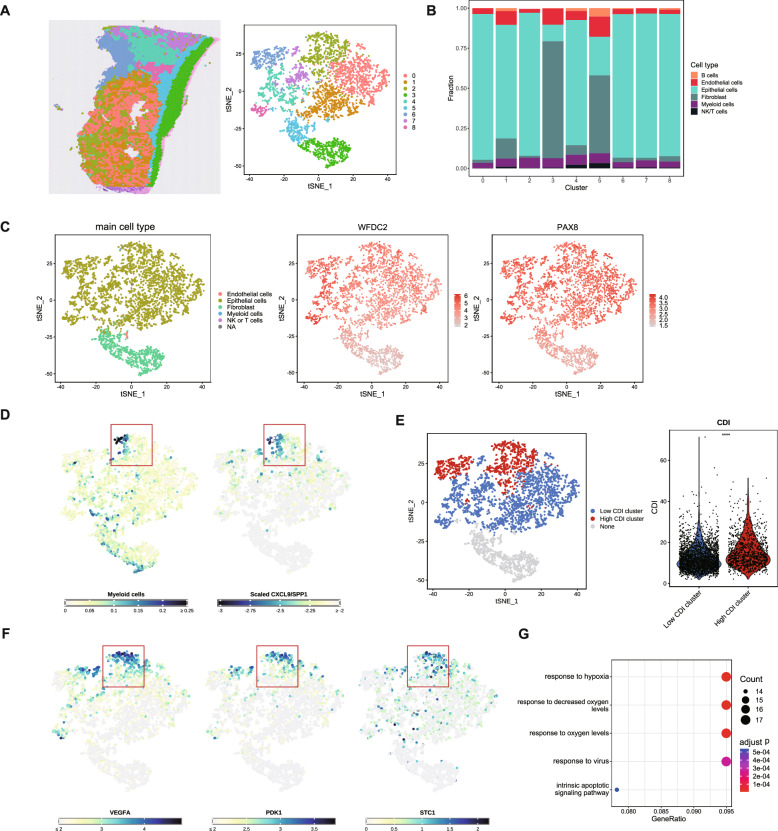
High CDI region overlaps with malignant areas of the tumor. **A** Projections and tSNE visualization of spot clusters from a patient with SOC. **B** Bar plots of cell proportions after deconvolution. **C** tSNE visualization of the main cell type for each spot and the expression of SOC marker genes. **D** tSNE visualization of the myeloid cell deconvolution and normalized CS values. **E** tSNE and violin plots showing the CDI group of clusters dominated by tumor cells. **F** tSNE visualization of angiogenesis-related gene expression. **G** Bubble plots showing the enriched GO terms (n = 5) of the top 200 upregulated genes in the high CDI cluster dominated by tumor cells

Next, we performed a spatial transcriptional analysis of the OC samples from 10 × Genomics (FFPE samples, HE staining), and pathologists annotated the samples to confirm the spatial relationship between the CDI and malignant tumor cells. Based on the spatial characteristics of the cells, Leuven clustering grouped all the cells into nine clusters (Fig. 9A). Deconvolution of the spatial transcriptome using the scRNA-seq data from Xu et al. (Xu et al. 2022) confirmed the cell composition of the nine clusters; the results showed that fibroblasts dominated clusters three and five, whereas the remaining clusters were mainly epithelial cells (Fig. 9B). The spatial distribution characteristics of each cell cluster are shown (Fig. S9A). The expression distribution of malignant markers of OC (WFDC2 and PAX8) and pathological identification enabled us to determine the aggregated areas of tumor cells (Fig. 9C; Fig. S9B).

Combining the distribution of myeloid cells, the expression of macrophage markers, CD14, APOE, C1QA, C1QC, and the scaled CXCL9/SPP1 (CS) ratio (Bill et al. 2023), we found macrophage aggregation associated with poor prognostic characteristics in the cluster 2 region (red box) (Fig. 9D; Fig. S9C). Based on the spatial distribution characteristics of the CDI and angiogenesis-related genes, we found that high CDI and angiogenesis characteristics (VEGFA + /PDK1 + /STC1 +) (Claesson-Welsh and Welsh 2013; Law and Wong 2013; Zhou et al. 2021) were co-located in cluster 2 (Fig. 9E and F). Angiogenesis may cause the formation of malignant ascites and metastasis in OC, accelerating its progression (Monk, Minion and Coleman 2016). We performed GO enrichment analysis on the top 200 genes, whose expression in the high CDI group was significantly higher than that in the low CDI group. Genes were significantly enriched in response to hypoxia (Fig. 9G), which possibly activates the expression of hypoxia-inducible factors (HIFs) and multiple angiogenic growth factors, contributing to intratumoral blood vessels (Wicks and Semenza 2022). Therefore, cluster 2 is likely to be a malignant tumor region. In summary, colocalizing the CDI with malignant markers of tumor cells in a space may predict the immune microenvironment and malignant regions of tumors.

### Identification of mutation landscape and tumor neoantigens

**Fig. 10:**
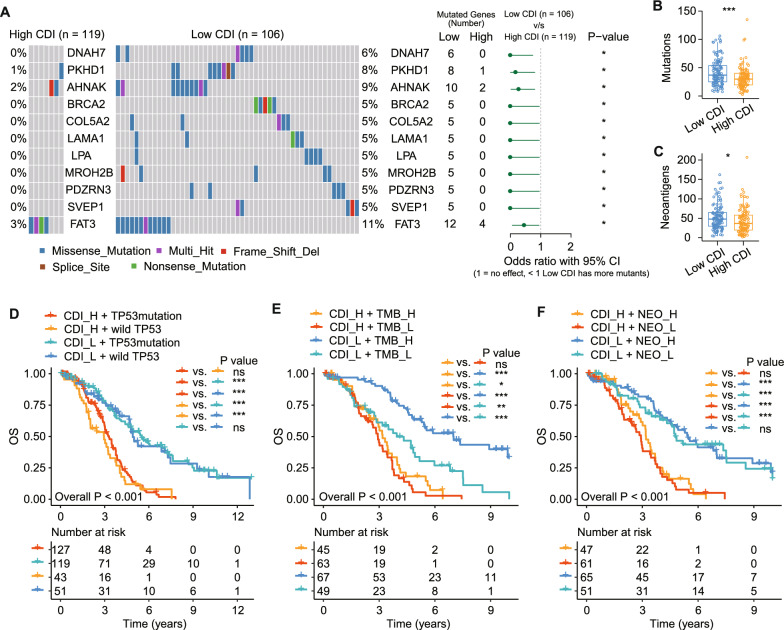
Identification of mutation landscape and tumor neoantigens. **A** Waterfall plot of differentiated somatic mutation features between the high and low CDI groups. **B, C** Mutations and neoantigen loads between the two subgroups are displayed.** D, E** and **F** Survival analyses of patients with SOC stratified by the CDI and mutation loads, TP53 status, or neoantigen burden using KM survival analysis. NEO, neoantigen burden; H, high; L, low. *P < 0.05, **P < 0.01, ***P < 0.001

The efficacy of immunotherapy is affected by various factors, such as immune infiltrates in the TME and the tumor mutational landscape. We speculated that patients with different CDI scores may show differences in tumor progression and immunotherapeutic responses. Furthermore, somatic mutations produce neoantigens that enhance tumor-specific immune responses, making neoantigens an emerging target for personalized immunotherapy (Xie et al. 2023). Therefore, we analyzed the somatic mutation frequency in patients with SOC to discover potential neoantigens. The top 20 most frequently mutated genes are displayed (Fig. S10A). Of these genes, the mutation rates of AHNAK and FAT3 were significantly higher in the low CDI group, whereas the other genes showed no statistical differences (Fig. S10B). Differentially mutated genes (DMGs) were identified and showed a high mutation frequency in the low CDI group (Fig. 10A), suggesting a cumulative effect of low-frequency mutations. Furthermore, we found higher tumor mutational burden (TMB) and neoantigen levels in the low CDI group (P_TMB_ < 0.001, P_Neoantigen_ = 0.015; Fig. 10B and C). Based on the KM analysis, when the CDI integrated with the TP53 mutation status, neoantigens, and TMB, patients with high TMB levels exhibited a longer OS than those with low TMB levels and a low CDI (Low CDI + Low TMB vs. Low CDI + High TMB, P < 0.001; Fig. 10D). Therefore, combining the CDI score with the TMB, we can accurately predict the prognosis of patients with SOC (Low CDI + High TMB vs. High CDI + Low TMB, P < 0.001; Fig. 10D). By contrast, the TP53 mutation status and neoantigens did not reliably predict survival in the patients with SOC (Low CDI + TP53 mutation vs. Low CDI + wild-type TP53, P = 0.77; High CDI + TP53 mutation vs. High CDI + wild-type TP53, P = 0.250; Fig. 10E; Low CDI + Low neoantigen vs. Low CDI + High neoantigen, P = 0.713; High CDI + Low neoantigen vs. High CDI + High neoantigen, P = 0.405; Fig. 10F). Thus, our model based on PCD is more effective in predicting survival than the existing biomarkers (Low CDI + TP53 mutation vs. High CDI + TP53 mutation, P < 0.001; Low CDI + wild TP53 vs. High CDI + wild TP53, P < 0.001; Low CDI + High neoantigen vs. High CDI + High neoantigen, P < 0.001; Low CDI + Low neoantigen vs. High CDI + Low neoantigen, P < 0.001; Low CDI + Low TMB vs. High CDI + Low TMB, P = 0.002; Low CDI + High TMB vs. High CDI + High TMB, P < 0.001) and shows great potential for use in selecting personalized treatment for patients with identical TP53, neoantigen and TMB status.

### Benefits of immunotherapy

**Fig. 11:**
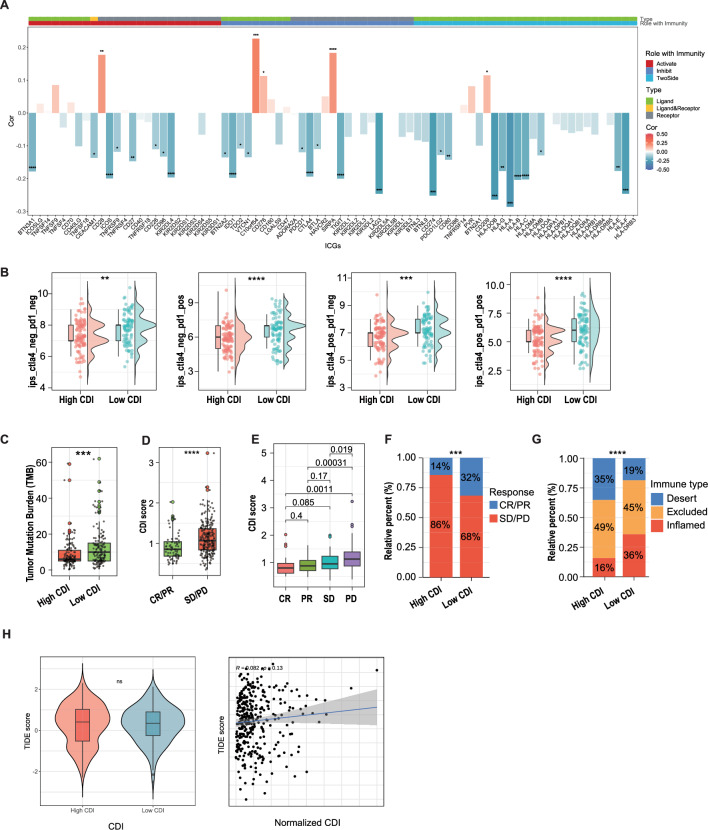
Evaluation of immunotherapy's benefits. **A** Bar plot of the correlation between immune checkpoint genes and CDI values in the TCGA-OV cohort. *: P < 0.05, **: P < 0.01, ***: P < 0.001. **B** The immunophenoscore scores of ips_ctla4_neg_pd1_neg (CTLA4-/PD1 − treatment), ips_ctla4_neg_pd1_pos (CTLA4-/PD1 + treatment), ips_ctla4_pos_pd1_neg (CTLA4 + /PD1 − treatment), and ips_ctla4_pos_pd1_pos (CTLA4 + /PD1 + treatment) between the high and low CDI groups. **C** Tumor mutational burden scores in the high and low CDI groups of the IMvigor210 cohort. **D** Differences in the CDI scores between patients with complete response (CR)/partial response (PR) and those with stable disease (SD)/progressive disease (PD) in the Imvigor210 cohort. **E** The variation in CDI scores among patients with CR, PR, SD, and PD in the Imvigor210 cohort. *P < 0.05, **P < 0.01, ***P < 0.001.** F** The differing response rates to immunotherapy between the high and low CDI groups in the Imvigor210 cohort. **G** Subgroup analysis based on immune phenotypes revealed differences between the high and low CDI groups in the immune-inflamed, immune-excluded, and immune-desert subtypes. **H** Tumor immune dysfunction and exclusion (TIDE) scores of the high and low CDI groups in the TCGA-OV cohort

Immune checkpoint (IC) molecules, such as programmed cell death 1 (PD-1)/programmed cell death ligand 1 (PD-L1) and cytotoxic T lymphocyte-associated molecule-4 (CTLA-4), significantly influence immune regulation. Upregulation of IC gene expression inhibits the anti-tumor immune response and facilitates tumor growth. In this regard, immune checkpoint inhibitors (ICIs) have been developed to achieve effective anti-tumor immunotherapy (Toor et al. 2020). The bar plot showed the correlation between IC gene expression and the CDI value in the TCGA-OV cohort. Most genes, including *PD-L1, CTLA4, LAG3, HLA-A, IDO1,* and *TIGIT*, showed higher expression in the low CDI group (Fig. 11A, Fig. S10C), indicating that the low CDI group was more likely to respond to ICIs. The immunophenoscore (IPS) was used to determine the sensitivity of high and low CDI groups to ICIs. Furthermore, scores for the four different treatments were calculated separately: (i) ips_ctla4_neg_pd1_neg (CTLA4-/PD1 − treatment); (ii) ips_ctla4_neg_pd1_pos (CTLA4-/PD1 + treatment); (iii) ips_ctla4_pos_pd1_neg (CTLA4 + /PD1 − treatment); and (iv) ips_ctla4_pos_pd1_pos (CTLA4 + /PD1 + treatment). IPS was significantly higher in the low CDI group receiving CTLA4 + /PD1 − and CTLA4 + /PD1 + treatments (Fig. 11B), suggesting that patients with a low CDI respond better to anti-CTLA4 or combined anti-CTLA4 and anti-PD1 therapy. In the IMvigor210 cohort, the low CDI group showed a higher TMB (Fig. 11C). Subsequently, we obtained CDI scores for complete response (CR)/partial response (PR) and stable disease (SD)/progressive disease (PD) cases, which showed that the CDI scores were significantly higher in patients with SD/PD than in those with CR/PR (Fig. 11D and E).

The chi-square test revealed that the proportion of CR/PR was higher in the low CDI group. In contrast, the proportion of SD/PD was higher in the high CDI group, indicating that the anti-PD1 antibody was more effective in the low CDI group (Fig. 11F). Immunophenotyping showed that the high CDI group was characterized by immune desert. However, the low CDI group was mainly characterized by immune inflammation (Fig. 11G). The TIDE score was used to predict the effects of immunotherapy (Fig. 11H). There was no statistically significant difference between the high and low CDI groups; however, we observed a positive correlation between the TIDE score and the CDI value, indicating that the low CDI group was less prone to tumor immune escape and that immunotherapy may be more effective for patients with SOC with a low CDI. In addition, IFNG and CD274 (PD-L1) scores were negatively correlated with CDI values. In contrast, exclusion, cancer-associated fibroblast (CAF), and M2-like tumor-associated macrophage (TAM) scores were positively correlated with CDI values, suggesting that immunotherapy may be more beneficial for patients with a low CDI (Fig. S10D). Overall, our results revealed that the PCD-genes model performed satisfactorily in predicting the benefits of immunotherapy. Thus, patients with a low CDI may be more suitable for immunotherapy and have better survival outcomes.

### Estimation of drug sensitivity and chemotherapy resistance

**Fig. 12:**
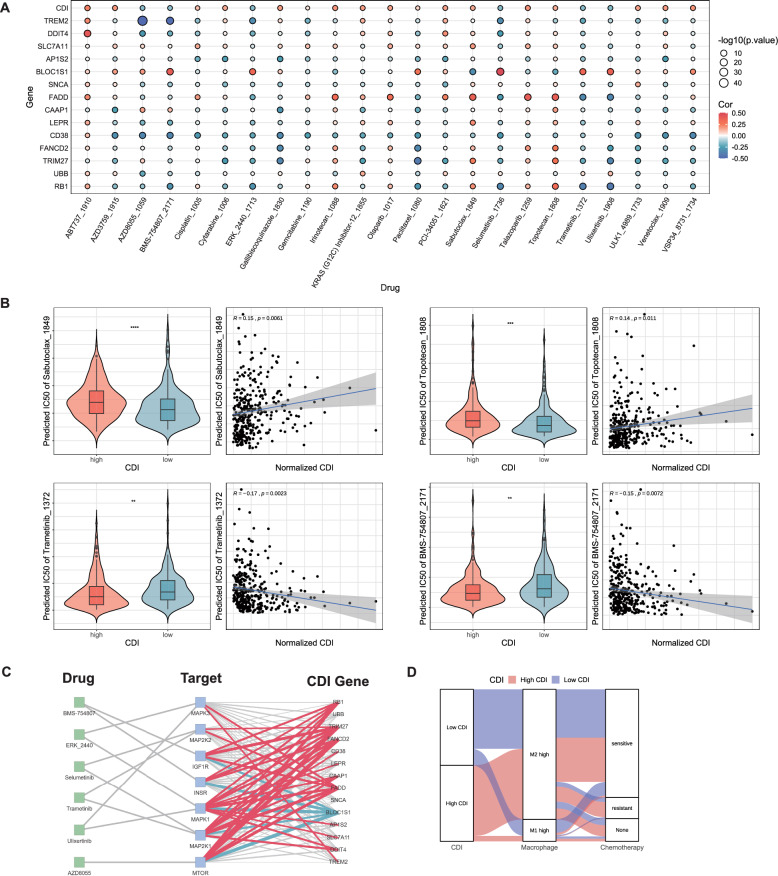
Estimation of drug sensitivity and chemotherapy resistance. **A** Bubble plot showing the relationship among drugs, model genes, and CDI. **B** Violin plots compare the drugs' half-maximal inhibitory concentration (IC50) between the high and low CDI groups in the TCGA-OV cohort. **C** Correlation between the model genes and classical therapeutic targets in SOC. The red and blue lines represent positive and negative correlations, respectively. The thickness of the lines represents the degree of correlation, and the gray line represents P > 0.05. **D** Alluvial diagram showing the relationship between macrophages, chemotherapy, and CDI in the TCGA-OV cohort

We focused on drugs commonly used by patients with cancer and identified the IC50 values of these anticancer drugs in SOC samples to explore the clinical utility of this PCD-genes model for drug sensitivity evaluation and anticancer drug selection. The therapeutic effects of the 20 representative anticancer drugs on SOC varied with the magnitude of the CDI (Fig. 12A). Among them, the four drugs with differential performance in the high and low CDI groups are shown (Fig. 12B), where Trametinib_1372 (the MEK inhibitor) and BMS-754807 (the dual IGF-1R/InsR tyrosine kinase inhibitor) showed lower IC50 values in the high CDI group; by comparison, Sabutoclax_1849 (the pan-active BCL-2 protein family antagonist) and Topotecan_1808 (Topoisomerase I inhibitor) exhibited higher IC50 values. This indicated that patients with SOC and a high CDI are more sensitive to Trametinib_1372 and BMS-754807, which may be therapeutic candidates for these types of patients. Patients with SOC and a high CDI may be prone to resistance to the other two drugs. Subsequently, we found a correlation between the 14 model genes and classical therapeutic targets for SOC (Fig. 12C). The alluvial diagram shows the correlation between the CDI, M1/M2 macrophages, and chemotherapy sensitivity; the low CDI group exhibited greater sensitivity to chemotherapy (Fig. 12D). These results indicated that our model showed good predictive ability for identifying drug sensitivity, which may enable patients to select more rational drug treatments.

## Discussion

### Advantages and innovations of using multi-omics to reveal the potential of programmed-cell death: a paradigm shift from reactive medicine to PPPM

Increasing evidence indicates that PCD significantly influences the malignant behavior of tumors and thus guides their treatment. For example, gastrointestinal cancer is associated with ferroptosis, necroptosis, and pyroptosis (Zhu and Li 2023). A combination of inducers of ferroptosis, necroptosis, and pyroptosis, as well as ICIs, provides synergism in the treatment of various types of tumors, such as small cell lung cancer (Niu et al. 2022; Tang et al. 2020). Regarding OC, researchers have summarized the mechanisms underlying autophagy, immunogenic cell death, pyroptosis, alkaliptosis, ferroptosis, and necroptosis. In addition, they proposed interventions to induce PCD initiation in tumor cells and emphasized the relevance of PCD-related gene expression to prognosis- and drug-efficacy-related prediction (Chen et al. 2022a; Zhang and Liu 2022). Prognostic models or risk scores based on expression levels and differences between PCD-related genes were used in various cancers, such as esophageal squamous cell carcinoma, hepatocellular carcinoma, lung adenocarcinoma, stomach adenocarcinoma, renal clear-cell carcinoma, providing new approaches for prognosis and guiding therapy (Cao et al. 2024; Chen et al. 2022b; Khan et al. 2022; Pan et al. 2022; Peng et al. 2022b; Wang et al. 2024; Zhang et al. 2022). Notably, two prognostic models, based on ferroptosis and necroptosis, have been constructed to predict the prognosis of patients with advanced OC treated with platinum drugs (Li et al. 2022b). Feng et al. confirmed that ferroptosis- and iron-metabolism-related long non-coding RNAs included in their model could independently predict OS and therapeutic effects in patients with OC (Feng et al. 2022). The expression of PCD-related genes and pathways may reflect patient prognosis; however, models capable of comprehensively guiding the relationship between PCD and OC are lacking, with more PCD-related genes needed to achieve a more accurate prognostic prediction.

Furthermore, multi-omics analysis incorporates data from different genetic levels, such as genomics, transcriptomics, and metabolomics, to comprehensively explore tumor characteristics and the role of PCD in tumors. Xiong et al. integrated whole genomics, CNV, and RNA sequencing data to classify and predict the characteristics of natural killer/T cell lymphoma (Xiong et al. 2020). Additionally, Qin et al. included various Cuproptosis genes to calculate individual Cuproptosis scores (CS). They combined them with multi-omics analyses to verify the prognostic role of CS in various cancers (Qin et al. 2023). The application of multi-omics technology can greatly deepen our understanding of tumor molecular mechanisms, their interactions, and the potential of PCD in cancer. The rapid development of various omics technologies has enabled researchers to dissect the complex molecular mechanisms underlying tumorigenesis and search for new biomarkers and drug targets, which may promote the practice of PPPM. This is due to some abnormalities at the DNA, RNA, protein, metabolite, and imaging levels associated with cancer being compounded by individual differences between the processes of diagnosis and treatment (Lu and Zhan 2018). Multi-omics data enables those concerned to clarify the complex pathogenesis of patients, provide treatment decisions, and realize the value of applying PPPM. Integrating multi-omics makes it possible to translate many research results into individualized and meaningful clinical guidance aimed at providing more precise medical services to patients within the PPPM framework.

In this study, we used PCD as the core, multi-omics analysis as the methodology, and the CDI as a predictive model for SOC for the first time, hoping to promote better implementation of PPPM in patients with SOC. The innovations associated with our model are as follows: first, multiple PCD genes and patterns provide a CDI with good foresight and prevention capabilities. In our study, which was not specific to a particular PCD, we selected 14 genes from 12 PCD patterns to construct reliable predictors of CDI. We considered the effects of multiple PCD on the prognosis of patients with SOC and the potential crosstalk between different forms of PCD, which ensured the accuracy of our model and enabled a more comprehensive understanding of the role of PCD in SOC. Second, we used multi-omics analysis to interpret and verify the developed model. Integrating genomics, single-cell transcriptomics, bulk transcriptomics, spatial transcriptomics, and clinical information enabled us to identify differences between the biological functions, tumor immune cell infiltration, immunotherapy responses, and drug sensitivity of patients with SOC. Multi-omics analysis provides personalized tumor characteristics for patients with SOC, which facilitates accurate treatment and suggests the potential molecular mechanisms underlying the type of cancer involved. Overall, given the potential of PCD and multi-omics in the context of PPPM, the predictive model in this study will greatly help improve the early identification of patients at high risk, accurate prognosis prediction, and personalized treatment strategies in patients with SOC.

### Predictive accuracy of CDI and CDI-based targeted prevention and personalized medicine services

SOC is a common type of ovarian malignancy, usually with asymptomatic development. Thus, SOC, especially poorly differentiated HGSOC, which is more invasive, exhibits rapid tumor progression and is already advanced at the time of discovery, leads to poor prognoses (Deng et al. 2022). The methods currently used by clinicians to manage SOC are based on imaging, histology, and serum markers, which lack sensitivity, making it challenging to achieve PPPM (Punzón-Jiménez et al. 2022). Considering the limited diagnostic options and poor prognoses for SOC, it is imperative to develop a promising individual prognostic indicator designed to stratify and predict the OS along with other cancer characteristics of patients with SOC. In the era of precision cancer medicine, an individual indicator is also a requirement for PPPM because it would allow for providing precise and personalized treatment for every patient to the maximum possible extent. We used the expression of 14 PCD-related genes and a new computational framework to construct a stable and accurate prognostic indicator, namely the CDI. Based on the CDI, patients with SOC were divided into high and low CDI groups, which was shown using the univariate and multivariate regression analyses to be an independent prognostic risk factor for patients with SOC. We found that a high CDI score was associated with a shorter survival time and a worse prognosis, leading to adverse clinical outcomes. Subsequently, the CDI was combined with multiple clinical characteristics (age, stage, and grade) to construct a tumor predictive model and a nomogram, which showed a good prognostic prediction effect and clinical utility in the training (TCGA-OV) and validation sets (GSE63885 + GSE26193 and GSE140082). This model, which we constructed, may be beneficial for the timely detection of patients with poor prognoses for SOC and for developing early and targeted interventions to improve patient outcomes in line with the PPPM concept.

Tumor cells release inflammatory mediators and intracellular components that alter the TME, leading to immune activation or suppression, which may complicate tumor treatment and prognosis (Liu et al. 2022). Considering the complexity and high heterogeneity of SOC, the PPPM approach is best suited for effectively helping physicians who are engaged in developing personalized treatment plans. We explored the impact exerted by differences in the TME and anti-tumor immunotherapy on the high and low CDI groups of patients with SOC, duly considering the complex association between PCD and TME. Functional enrichment analysis and scRNA-seq showed that the high CDI group was associated with a high activity of tumor signaling pathways, such as EMT, angiogenesis, and hypoxia, implying that tumors in patients with a high CDI were capable of more invasion and metastasis, which may lead to poor survival outcomes. Furthermore, M2 macrophages were significantly upregulated in the high CDI group, whereas M1 macrophages were upregulated in the low CDI group. M1 and M2 macrophages are important TME components and differentiate from TAM under different stimuli (Liu et al. 2021). Both macrophages are involved in tumor-associated inflammatory responses; however, M1 mainly promotes inflammation and tumor inhibition. At the same time, M2 acts as an immunosuppressive factor, which promotes tumor growth and metastasis, including angiogenesis, neovascularization, matrix activation, and remodeling (Liu et al. 2021). A high M2/M1 ratio is associated with poor prognosis in most tumors, and thus an elevated M2/M1 ratio in OC is considered to be a predictor of poor prognosis (Larionova et al. 2020; Schweer et al. 2022; Wang et al. 2021; Zhou et al. 2022). We found that the M2/M1 ratio was positively correlated with a high CDI, predicting a poor prognosis for patients with SOC. Therefore, increasing M1 macrophage polarization or decreasing M2 macrophage polarization may be considered a therapeutic approach, as for SOC (Li et al. 2022a).

Immunotherapy is a promising clinical strategy for treating multiple cancer types, including SOC. However, the response and efficacy of immunotherapy in different patients need to be validated, as only a subset of patients may benefit from it (Hu et al. 2022; Zhang and Zhang 2020). Therefore, difficulties faced by physicians involved in decision-making regarding the use of immunotherapy for a particular patient are detrimental to the efficacy of the PPPM approach in treating SOC. Thus, based on the PCD gene model, we examined the efficacy of immunotherapy in different CDI groups to provide personalized treatment strategies within the context of PPPM. Moreover, TMB measures the number of gene mutations in cancers, with a higher TMB resulting in more neoantigens, predicting a better immunotherapy response (Jardim et al. 2021). In our study, patients with a low CDI had more mutations and neoantigens than those with a high CDI; these additional mutations and neoantigens improved the sensitivity to immunotherapy in patients with a low CDI. ICIs are the most thoroughly investigated and critically important immunotherapeutic agents, and the clinical application of PD-1 or PD-L1 and CTLA-4 inhibitors has improved the OS of various patients with cancer (Riley et al. 2019). We found that the IC expression in the low-CDI group was higher than that in the high-CDI group, suggesting that targeting IC molecules may be effective as a tumor suppression treatment for patients with SOC and a low CDI. Furthermore, the low CDI group had higher IPS scores, higher response rates to anti-PD1, and better survival outcomes, indicating that patients with a low CDI may respond better to treatment with ICIs.

In this study, we also found that the TIDE score was positively correlated with the CDI value, suggesting a low possibility of tumor immune escape in the low CDI group, further substantiating our view. Overall, the immune infiltration situation prompts us to propose the "hot tumor" / "cold tumor" concept (Galon and Bruni 2019), in which the low CDI group with higher immune cell infiltration and immune checkpoint expression represents "hot tumors", whereas it is the opposite for the high CDI group. The former is more suitable for immunotherapy, whereas immunotherapy aimed at the latter is hardly beneficial. Considering the potential of immunotherapy, the "hot/cold tumor" concept is an important complement to PPPM. We emphasize using the CDI to distinguish hot tumors from cold tumors in SOC, promoting personalized patient treatment, reducing unnecessary medical costs, and increasing the clinical benefits of immunotherapy.

Drug resistance and tumor recurrence remain problematic, posing huge obstacles to the treatment (Chandra et al. 2019) despite the use of tumor reduction surgery, radiotherapy, chemotherapy, new targeted therapy, and immunotherapy for OC. We hope that our prediction model based on PCD genes will facilitate the search for new targeted drugs, improve the prognosis of patients with OC, and lead to more advanced medical PPPM strategies. Notably, data from a predictive model for drug sensitivity suggested that patients with a high CDI may be potentially resistant to Topotecan_1808 and Sabutoclax_1849; however, Topotecan_1808 has been widely used to treat recurrent OC (Brogden and Wiseman 1998). The MEK inhibitor, Trametinib_1372, is effective for progressive or recurrent LGSOC (Gershenson et al. 2022). BMS-754807, with dual IGF-1R/InsR tyrosine kinase inhibition, has promising anti-tumor activity against various cancers, including breast, pancreatic, and lung cancers (Awasthi et al. 2012; Hou et al. 2011; Zhang et al. 2023). Both drugs showed high sensitivities in the high CDI group. Hence, we speculate that Trametinib_1372 and BMS-754807 may be potential therapeutic options for patients with SOC and a high CDI. Therefore, targeting PCD in cancer cells appears to be a promising therapeutic strategy. Targeting specific genes or regulating the PCD signaling pathway may stimulate autophagy-dependent cell death, apoptosis, and pyroptosis, inducing cancer cell death or inhibiting drug resistance capabilities of cancer cells, achieving anti-tumor objectives (Peng et al. 2022a). In breast cancer, autophagy is now a new therapeutic target, where several small-molecule compounds may alter the autophagy levels of cancer cells by targeting signaling pathways and proteins, such as the unc-51-like kinase 1 complex, PI3KC1-Akt-mTORC1, Ras-Raf-MAPKs, and p53, improving breast cancer treatment efficacy by overcoming drug resistance (Liao et al. 2022). Naturally derived indole alkaloids may regulate ferroptosis, necroptosis, and autophagy in cancer and thus play a prominent role in developing anticancer agents (Qin et al. 2022). Furthermore, combined correlation analysis of model genes and classical therapeutic targets showed that our selected 14 PCD model genes, which were differentially expressed between SOC and normal ovarian tissues, contained genes that were closely associated with the IGF1R, INSR, and mTOR targets and the MAPK pathway. Therefore, targeting these genes or related signaling pathways may regulate PCD activity, prevent cancer cells from escaping cell death, and provide new options for cancer treatment, optimizing the development and application of anti-OC drugs.

### Molecular mechanisms reveal the working principle of CDI

Multi-omics analysis may help identify changes in cancer cells with different degrees of malignancy at the molecular level. However, further investigation of the molecular mechanisms underlying SOC may form a basis for establishing reliable biomarkers and optimizing PPPM. Discussing and analyzing the differences in pathway enrichment and gene mutations between high and low CDI groups may help reveal those molecular mechanisms underlying the CDI in SOC, helping to clarify the working principles of the CDI, optimize the accuracy of the prediction model, characterize more potential mechanisms and prognostic markers, and provide directions for improving PPPM in SOC.

Both EMT and angiogenesis were enriched in the high CDI group. EMT is a reversible biological process in which epithelial cells are transformed into mesenchymal cells with interstitial phenotypes. It is recognized as a malignant behavioral pattern that enables cancer cells to develop and metastasize. Additionally, mesenchymal phenotype cells interact with the TME and recruit pro-tumorigenic M2 macrophages (Dongre and Weinberg 2019). This is consistent with our observations of M2 macrophage aggregation and poor prognosis in the high CDI group. Furthermore, angiogenesis is an oncogenic pathway where newly disordered vasculature is conducive to tumor invasion, metastasis, and drug resistance. Activation of angiogenesis is closely associated with poor prognosis in breast cancer, and the proposed targeted treatment strategies aimed at inhibiting angiogenesis based on angiogenesis-related markers may effectively improve therapeutic strategies (de Heer et al. 2020). Therefore, angiogenesis contributes to the worse prognoses for patients with a high CDI and is an essential factor influencing the selection of treatment strategies. Moreover, single-cell RNA transcriptome data from patients with SOC showed enrichment of the hypoxic pathway in the high CDI cluster. HIFs can activate the EMT and angiogenic pathways mentioned above and other cancer malignant pathways, such as cancer stem cell specification, extracellular matrix remodeling, and cell motility, leading to immune escape and the invasion of cancer cells (Wicks and Semenza 2022). The single-cell RNA transcriptome analysis results were confirmed using spatial transcriptomic analysis. The above research results make the predictive ability of CDI more convincing.

In conclusion, an in-depth exploration of differentially activated pathways and expressed genes between the high and low CDI groups supports the significance of the CDI in PPPM and explains why patients with a high CDI have a worse prognosis. Additionally, it provides references for scholars interested in the crosstalk between PCD and other malignant pathways in cancer.

### Limitations

This study has some limitations. First, bias could not be avoided since this was a retrospective study of collected data. Hence, multicenter, large-scale studies may be required to validate our model. Second, a study of patients with SOC receiving relevant treatments should be conducted to characterize the ability of the model to predict prognoses, immunotherapy efficacy, and drug sensitivity. Third, CDI certainly still requires validation based on a large prospective cohort. Finally, the data used to analyze and build the model in our study were obtained from SOC samples. Thus, investigations are needed to understand whether PCD-related genes exert similar effects on other cancers. Pan-cancer analysis revealed the CNV percentage and correlations of CNV with the mRNA expression of 14 PCD-related genes in each cancer, demonstrating a correlation between the variant landscape of PCD-related genes and cancer characteristics, including the activity of cancer-related pathways and immune cell infiltration in the pancreas (Fig. S11). As a potential predictive method, the established model based on PCD-related genes may apply to various cancers. Thus, detailed studies exploring the correlation between PCD-related genes and different cancers, using multi-databases and multi-omics, would be helpful in this regard.

## Conclusion

In this study, we began with 12 PCD patterns (apoptosis, necroptosis, pyroptosis, ferroptosis, cuproptosis, entotic cell death, NETotic cell death, parthanatosis, lysosome-dependent cell death, autophagy-dependent cell death, alkaliptosis, and oxeiptosis) and systematically screened for PCD-related genes that were differentially expressed between SOC and normal tissues. Finally, 14 PCD-related genes were selected using stringent criteria to construct a PCD-gene model, namely CDI, which was found to be an independent prognostic factor with high accuracy and clinical utility. The model was validated via scRNA-Seq, bulk RNA-Seq, spatial transcription, and genomic analyses. The CDI can predict the survival status and prognosis of patients with SOC, reveal complex TME cell infiltration characteristics, detect drug resistance, and provide new insights into SOC prognosis and treatment. Thus, these findings may lead to a reliable support system for targeted prevention and individualized treatment of SOC, enhancing the implementation of clinical treatment in the context of PPPM.

## Supplementary Information

Additional file 1.

## Data Availability

The TCGA and GEO databases are publicly available. All datasets were downloaded directly from the indicated websites. The datasets and custom scripts are available upon request.

## Abbreviations

AUC: Area under the curve
CDI: Cell death index
EMT: Epithelial–mesenchymal transition
IC: Immune checkpoint
OC: Ovarian cancer
OS: Overall survival
PPPM/3PM: Predictive, preventive, and personalized medicine
PCD: Programmed cell death
TMB: Tumor mutational burden
TME: Tumor microenvironment
