# Supplementary material for: Multi‑omics identification of a novel signature for serous ovarian carcinoma in the context of 3P medicine and based on twelve programmed cell death patterns: a multi-cohort machine learning study
Source: Mol Med. 2025 Jan 8;31:5. doi: 10.1186/s10020-024-01036-x (PMC11707953; doi:10.1186/s10020-024-01036-x)
Supplement: Supplementary file 1 — Additional file 1. [file 10020_2024_1036_MOESM1_ESM.docx]

Supplementary Information


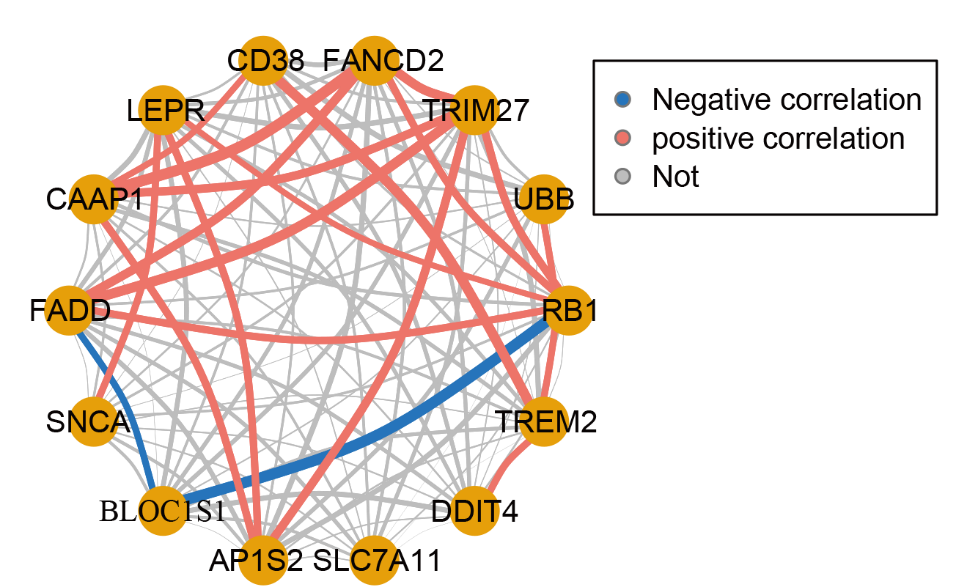


Supplementary Fig. 1. Correlation network of CDI model genes.


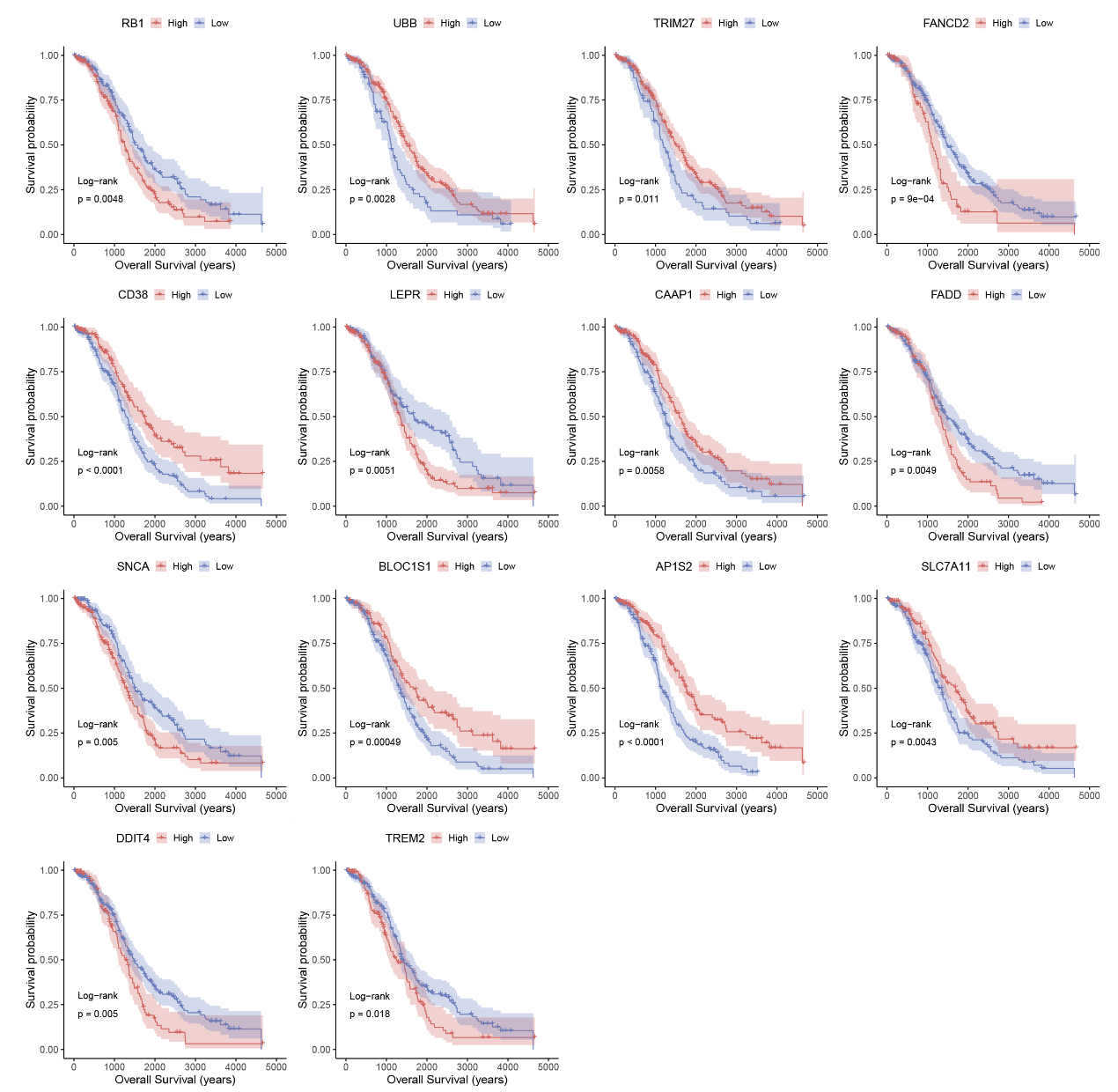


Supplementary Fig. 2. Kaplan–Meier survival curve for OS of SOC patients grouped by each model gene expression in TCGA-OV cohort.


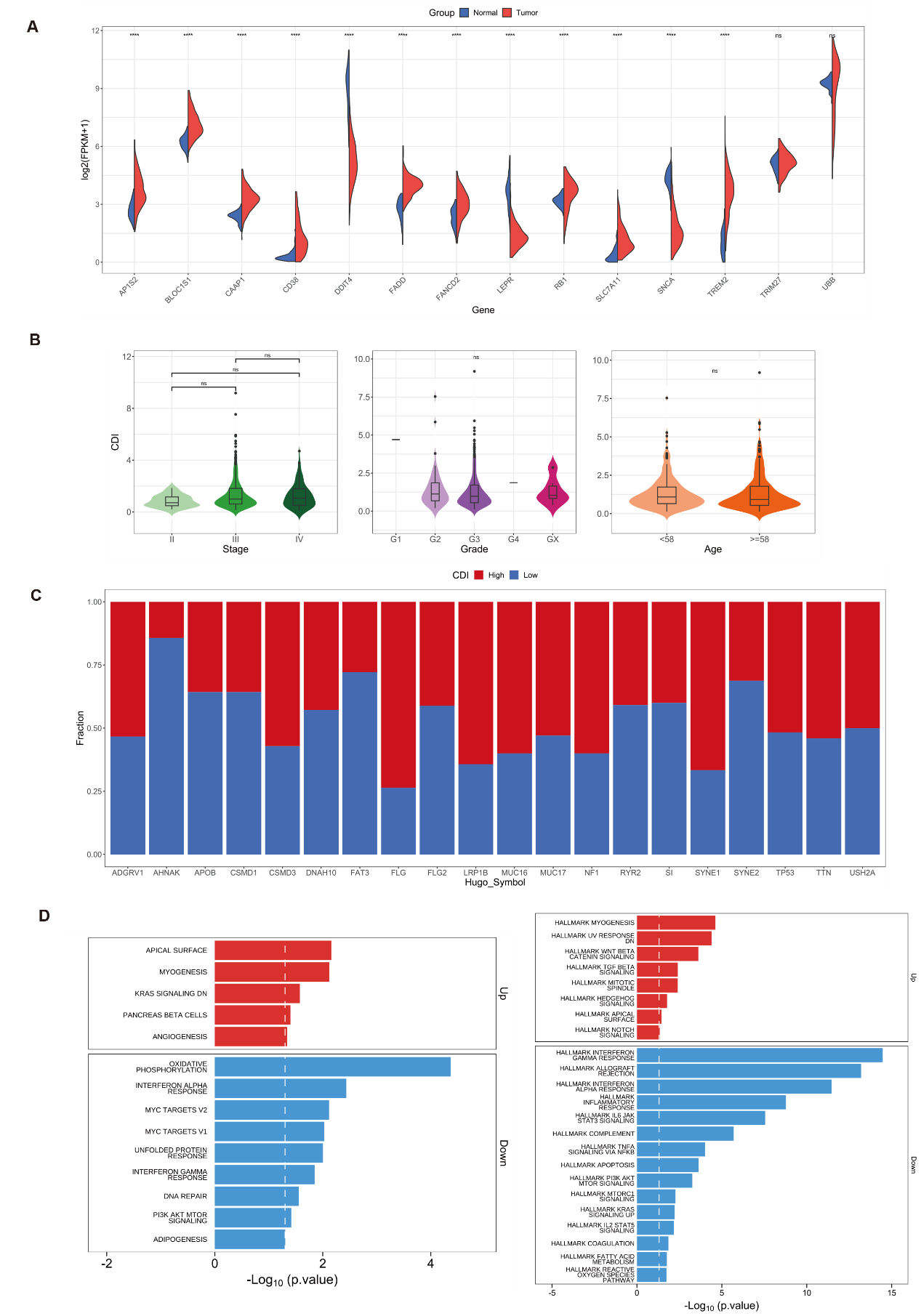


Supplementary Fig. 3. (A) The expression level of model genes in SOC patients from Tumor (TCGA-OV) and Normal (GTEx). (B) Violin plots of the relationship between CDI and stage, grade, age in TCGA-OV cohort.


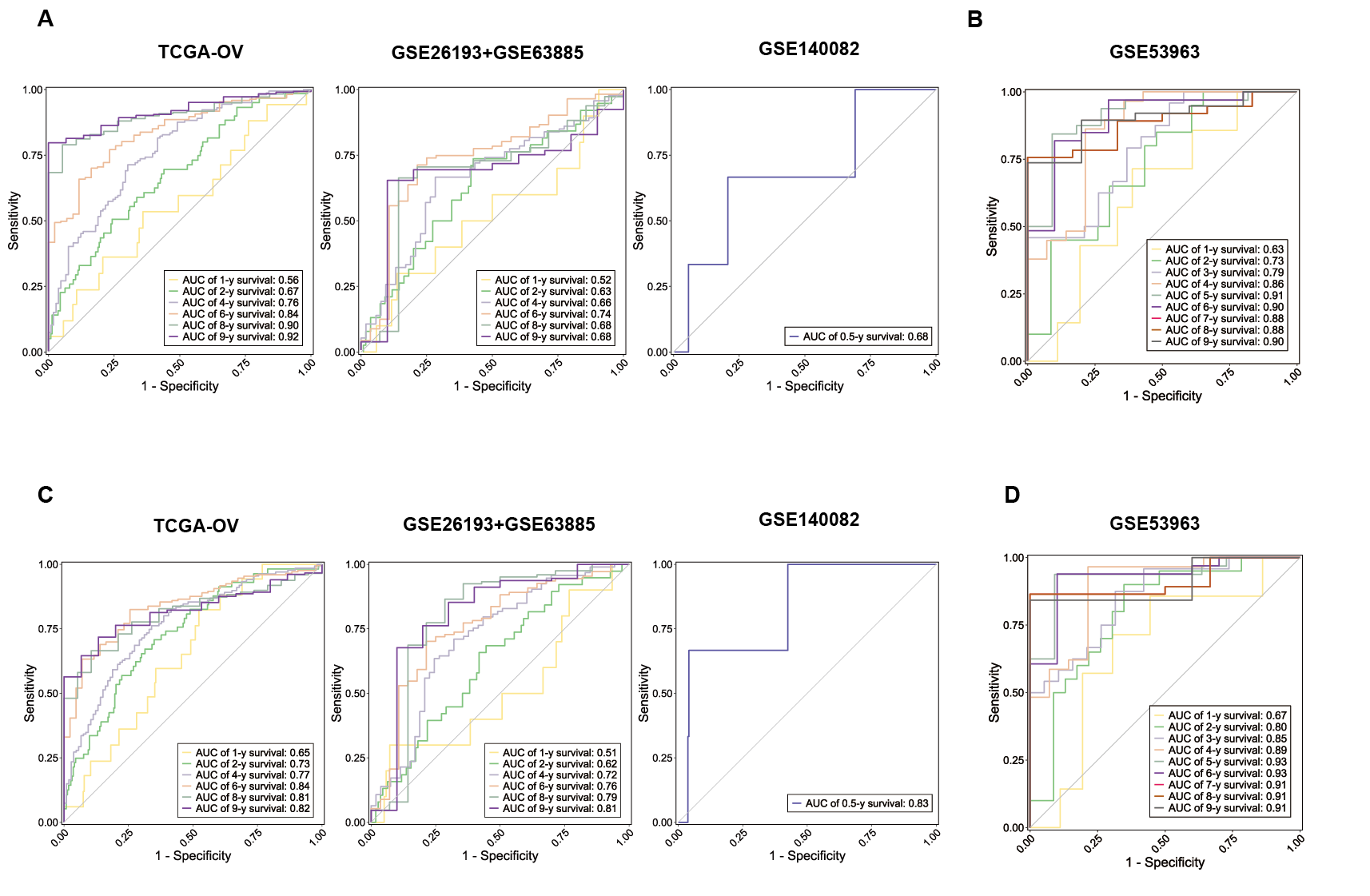


Supplementary Fig. 4. Receiver operating characteristic (ROC) analysis of the model and nomogram in the TCGA-OV, GSE63885+GSE26193, GSE140082, and GSE53963 cohort. (A) ROC analysis of the model in the TCGA-OV, GSE63885+GSE26193, and GSE140082 cohort. (B) ROC analysis of the model in the GSE53963 cohort. (C) ROC analysis of the nomogram in the TCGA-OV, GSE63885+GSE26193, and GSE140082 cohort. (D) ROC analysis of the nomogram in the GSE53963 cohort.


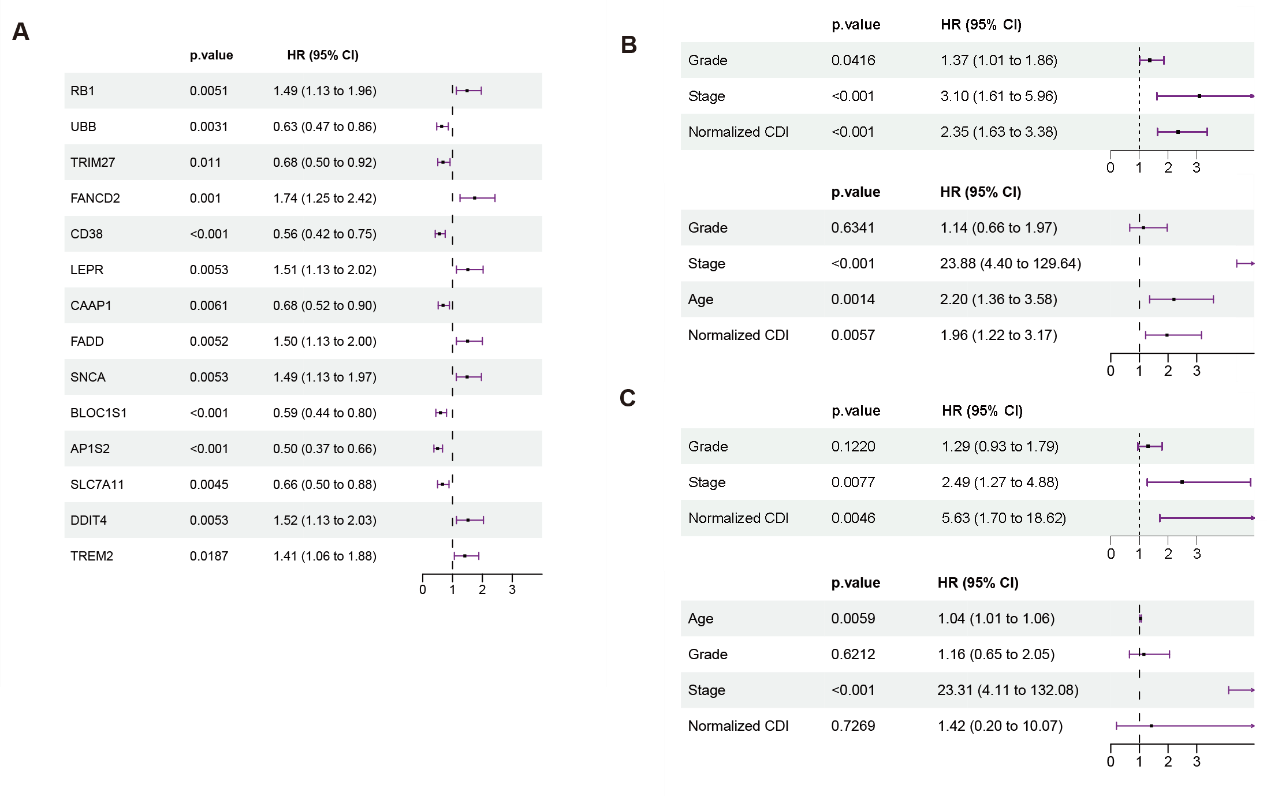


Supplementary Fig. 5. Forest plots for univariate and multivariate Cox analysis in multiple cohorts. (A) Univariate Cox analysis of model genes in TCGA-OV cohort. (B & C) Univariate and multivariate Cox analysis integrating CDI and clinicopathological indexes based on GSE63885+GSE26193 (B) and GSE140082 (C) cohorts.


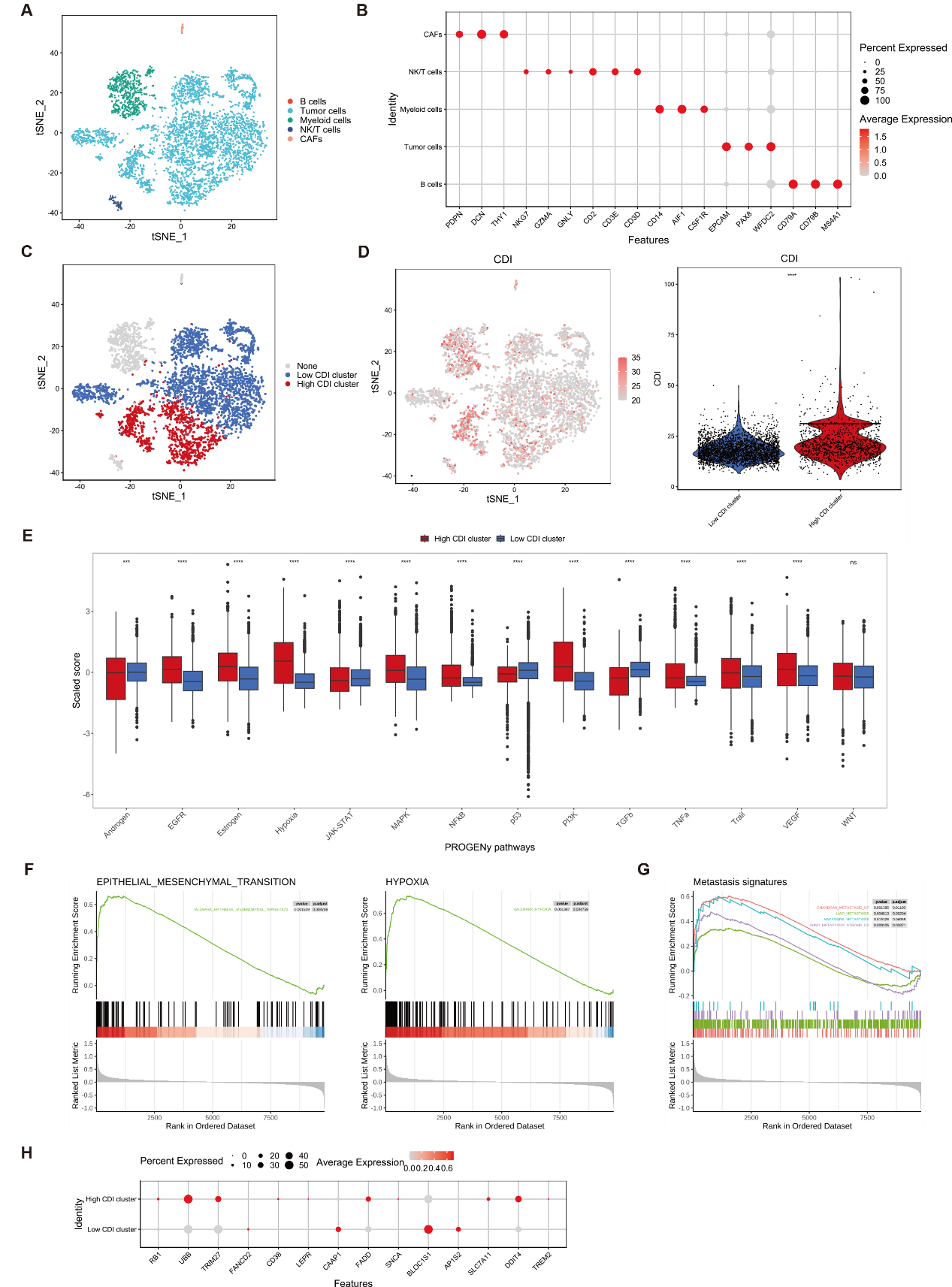


Supplementary Fig. 6. Single cell transcriptome analysis reveals the association between CDI and malignant tumor cells in GSE213243 cohort, Related to Figure 7. (A) tSNE visualization of diverse cell types in tumor sample from GSE213243, colored by each cell type. (B) Bubble plots of cell-type marker genes expression. (C) tSNE visualization of the CDI cluster group of tumor cells. (D) tSNE and Violin plot showing CDI values of tumor cells. (E) Box plot of the normalized activity of cancer-relevant pathways. (F) GSEA showing the hallmark epithelial-mesenchymal transition gene set and hypoxia are enriched in high CDI cluster. (G) GSEA showing high CDI cluster was enriched for elevated metastasis signatures.


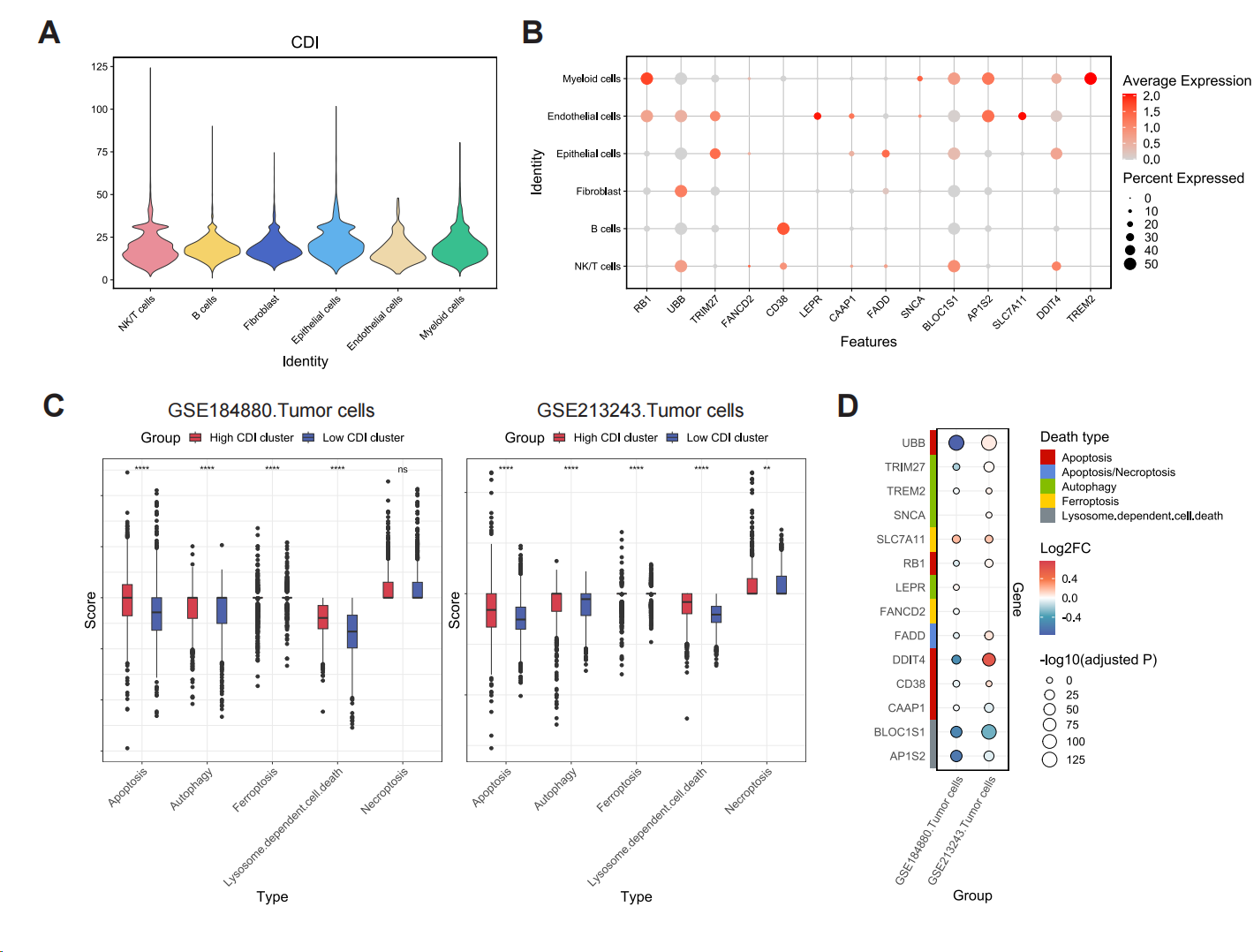


Supplementary Fig. 7. (A) Violin plot showing CDI values of cells from GSE184880 that Distinguished by cell type. (B) Bubble plots of CDI model genes expression in different types of cells from GSE184880. (C) Scores of tumor epithelial cells calculated by different death-type genes and corresponding parameters from CDI model. (D) Bubble plots of model genes expression in tumor epithelial cells.


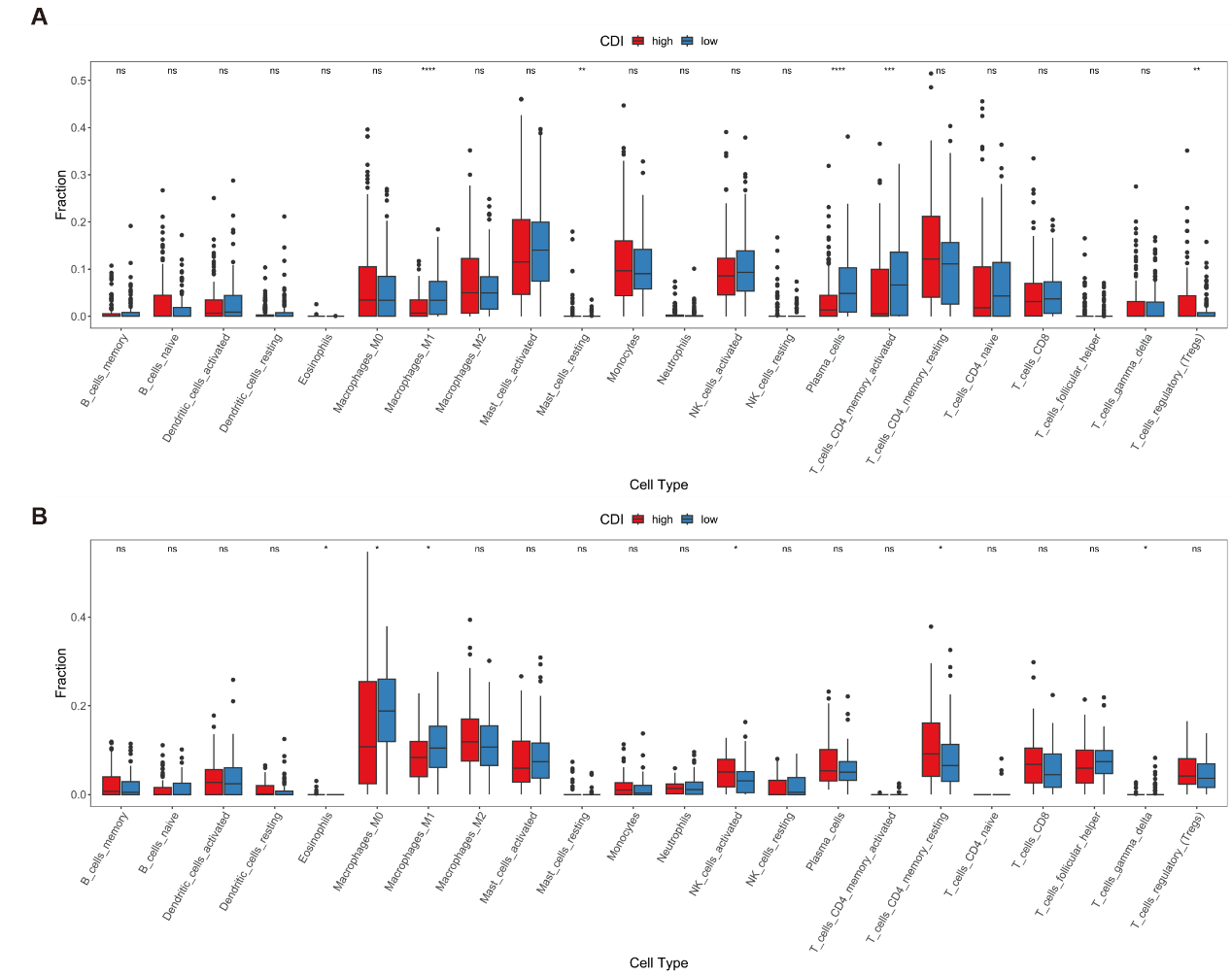


Supplementary Fig. 8. (A & B) Box plots of the proportion of 22 kinds of immune cells predicted by CIBERSORT in GSE63885+GSE26193 (A) and GSE140082 (B).


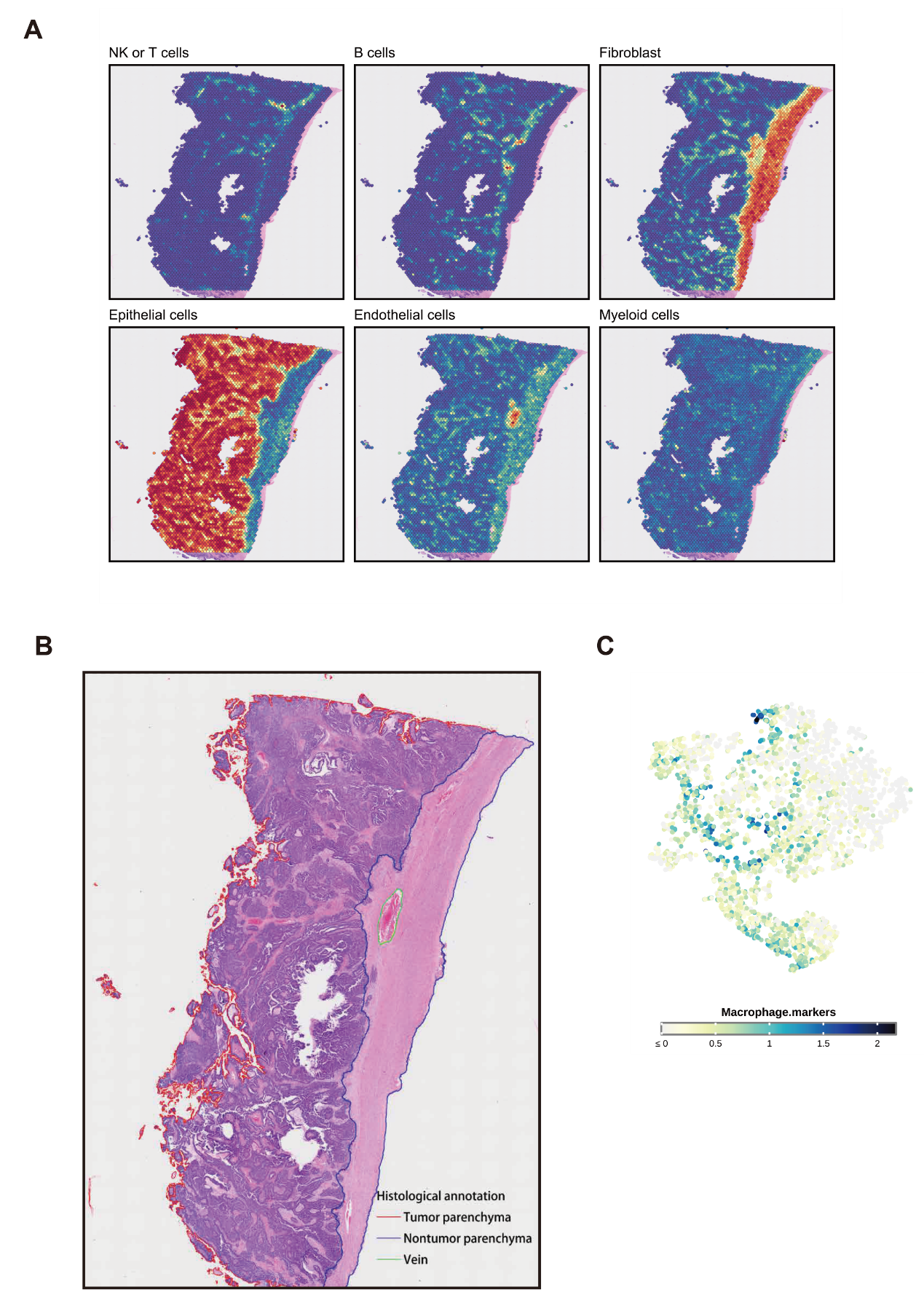


Supplementary Fig. 9. Supplementary results of spatial transcriptome, Related to Figure 9. (A) projection of cell proportions from deconvolution onto H&E image. (B) Overview of SOC tissue histology. (C) tSNE visualization of macrophage marker score.


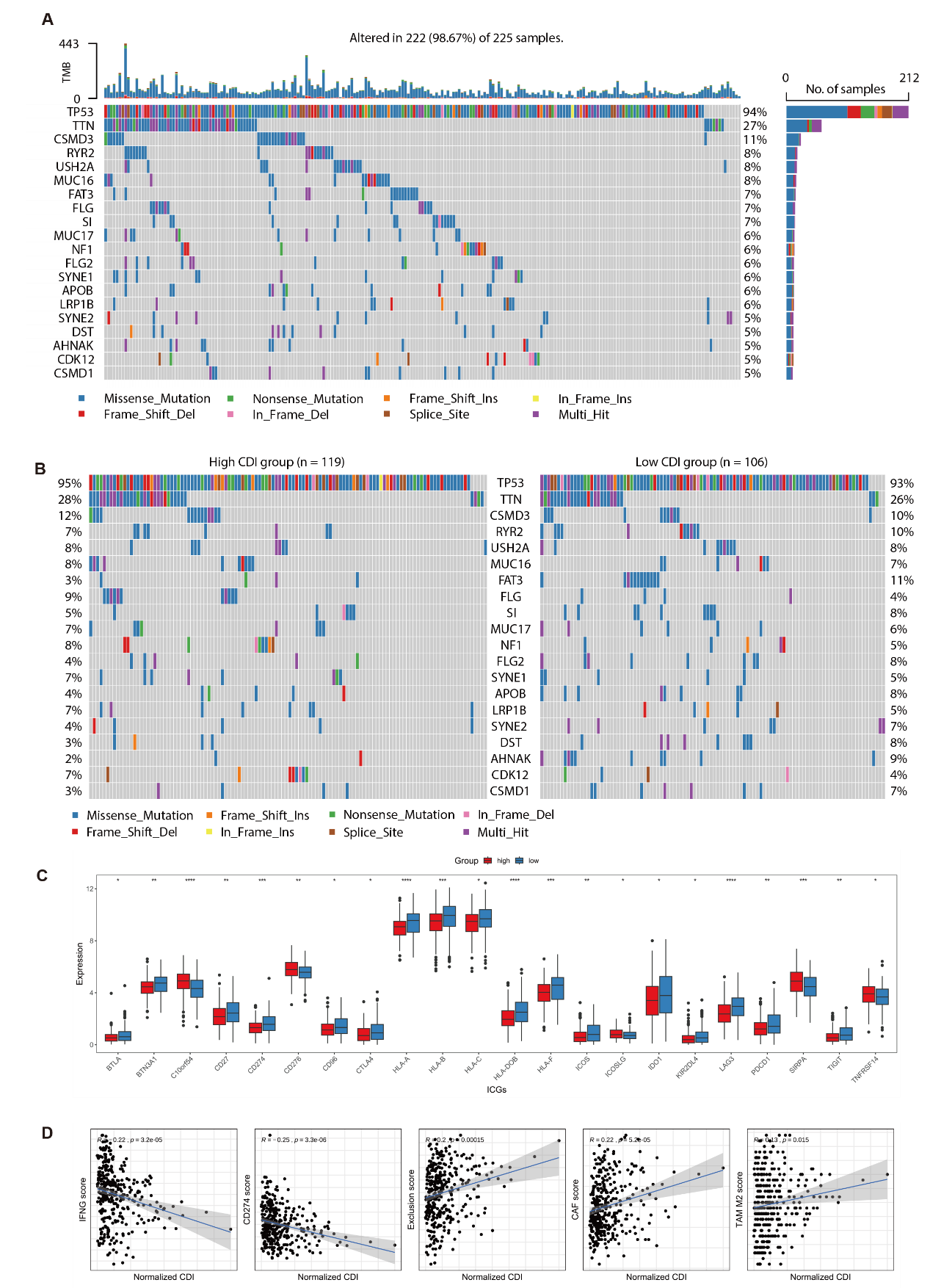


Supplementary Fig. 10. (A) Waterfall of mutation frequencies of top 20 genes in SOC patients. (B) The differences in top 20 mutation frequencies of genes in SOC patients of the high- and low- CDI groups. *:P< 0.05, **:P< 0.01, ***:P<0.001. (C) Box plot of expression of immune checkpoint genes in TCGA-OV. (D) IFNG, CD274, Exclusion, CAF, TAM M2 enrichment scores from TIDE module.


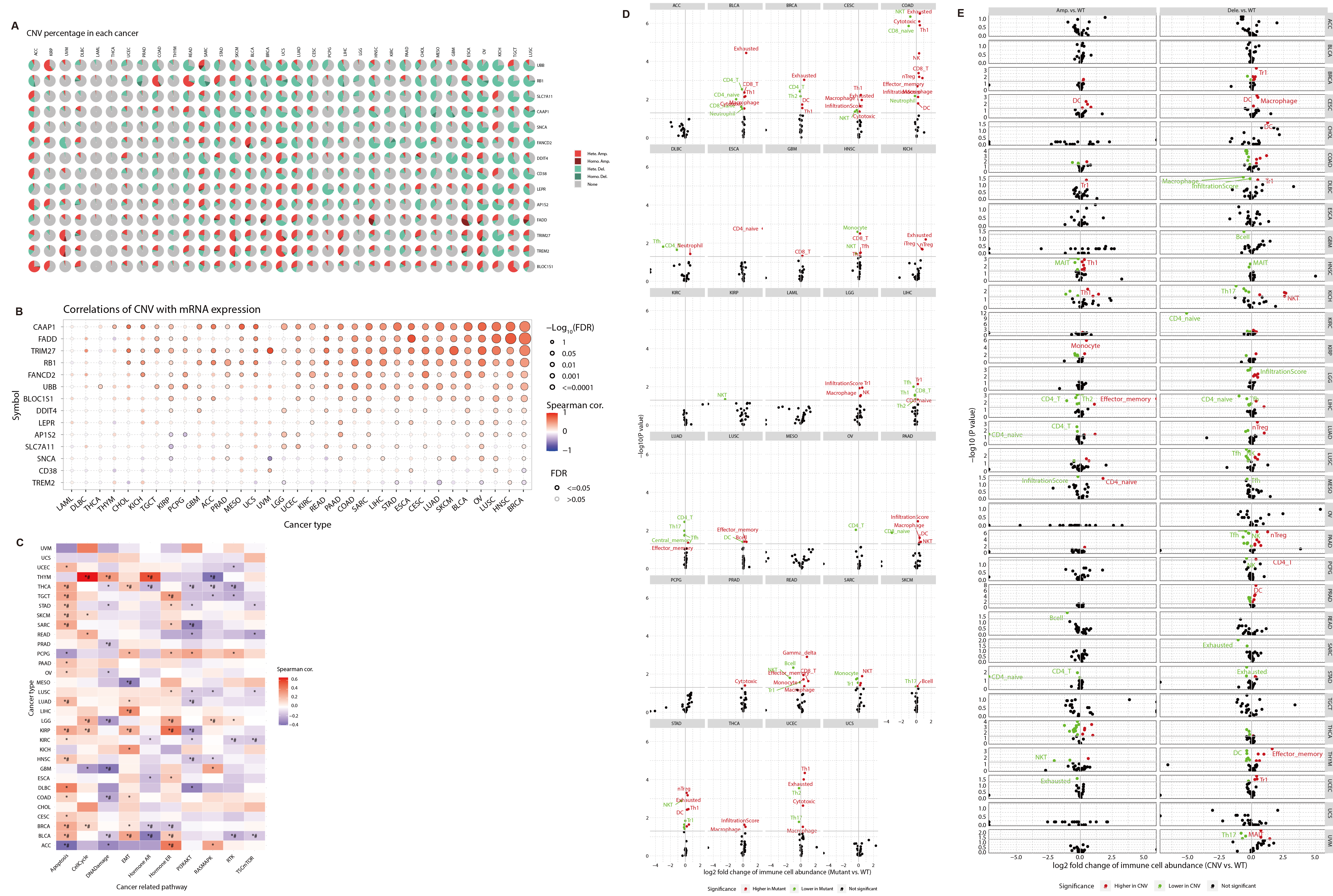


Supplementary Fig. 11. Integrated estimation of 14 PCD-genes in the aspect of mRNA expression, CNV, and SNV levels in Pancancers. (A) Pie plot summarizes the CNV percentage of 14 PCD-genes in 33 TCGA-OV cancers. Hete Amp: heterozygous amplification, CNV = 1; Hete Del: heterozygous deletion, CNV = -1; Homo Amp: homozygous amplification, CNV = 2; Homo Del: homozygous deletion, CNV = -2. (B) Correlations of CNV with mRNA expression for 14 PCD-genes in Pancancers. Size represents false discovery rate (FDR) value, the larger the circle, the smaller the FDR value. Black circles represent FDR less than or equal to 0.05 and gray circles represent FDR larger than 0.05. Red: positive correlation; Blue: negative correlation. (C) The correlation between GSVA score of PCD-genes model and activity of cancer related pathways in Pancancers. Red: positive correlation; Blue: negative correlation. *: P value ≤ 0.05; #: FDR ≤ 0.05. (D) The difference of immune cell infiltration between Mutant vs. WT groups of PCD-genes in Pancancers. Red: high in Mutant; Green: lower in Mutant; Black: not significant. (E) The difference of immune cell infiltration between CNV vs. WT of PCD-genes in Pancancers. Red: higher in CNV; Green: lower in CNV; Black: not significant.

Additional Tables

Table S1. Information on twelve types of PCD-related genes.

| Gene description | Gene name | Gene stable ID | PCD Type |
| --- | --- | --- | --- |
| inhibitor of nuclear factor kappa B kinase subunit beta | IKBKB | ENSG00000104365 | Alkaliptosis |
| carbonic anhydrase 9 | CA9 | ENSG00000107159 | Alkaliptosis |
| nuclear factor kappa B subunit 1 | NFKB1 | ENSG00000109320 | Alkaliptosis |
| component of inhibitor of nuclear factor kappa B kinase complex | CHUK | ENSG00000213341 | Alkaliptosis |
| BR serine/threonine kinase 2 | BRSK2 | ENSG00000291428 | Apoptosis |
| cell death inducing DFFA like effector b | CIDEB | ENSG00000285199 | Apoptosis |
| nuclear factor of activated T cells 4 | NFATC4 | ENSG00000285485 | Apoptosis |
| BCL2 like 14 | BCL2L14 | ENSG00000281449 | Apoptosis |
| caspase 8 associated protein 2 | CASP8AP2 | ENSG00000288475 | Apoptosis |
| phospholipid scramblase 3 | PLSCR3 | ENSG00000284009 | Apoptosis |
| transmembrane protein 102 | TMEM102 | ENSG00000284228 | Apoptosis |
| hypoxia up-regulated 1 | HYOU1 | ENSG00000280682 | Apoptosis |
| POU class 4 homeobox 1 | POU4F1 | ENSG00000152192 | Apoptosis |
| scratch family transcriptional repressor 2 | SCRT2 | ENSG00000215397 | Apoptosis |
| EYA transcriptional coactivator and phosphatase 2 | EYA2 | ENSG00000064655 | Apoptosis |
| microRNA 16-1 | MIR16-1 | ENSG00000208006 | Apoptosis |
| microRNA 15a | MIR15A | ENSG00000283785 | Apoptosis |
| phosphatase and tensin homolog | PTEN | ENSG00000284792 | Apoptosis |
| prostaglandin I2 synthase | PTGIS | ENSG00000124212 | Apoptosis |
| snail family transcriptional repressor 1 | SNAI1 | ENSG00000124216 | Apoptosis |
| SRC proto-oncogene / non-receptor tyrosine kinase | SRC | ENSG00000291971 | Apoptosis |
| poly(ADP-ribose) polymerase 2 | PARP2 | ENSG00000291803 | Apoptosis |
| serine/threonine kinase 24 | STK24 | ENSG00000102572 | Apoptosis |
| serine incorporator 3 | SERINC3 | ENSG00000132824 | Apoptosis |
| BCL2 interacting killer | BIK | ENSG00000100290 | Apoptosis |
| HRas proto-oncogene / GTPase | HRAS | ENSG00000276536 | Apoptosis |
| caspase 12 (gene/pseudogene) | CASP12 | ENSG00000291983 | Apoptosis |
| sushi repeat containing protein X-linked | SRPX | ENSG00000101955 | Apoptosis |
| PTTG1 interacting protein | PTTG1IP | ENSG00000183255 | Apoptosis |
| tyrosine 3-monooxygenase/tryptophan 5-monooxygenase activation protein eta | YWHAH | ENSG00000128245 | Apoptosis |
| phorbol-12-myristate-13-acetate-induced protein 1 | PMAIP1 | ENSG00000141682 | Apoptosis |
| somatostatin receptor 3 | SSTR3 | ENSG00000278195 | Apoptosis |
| PLAG1 like zinc finger 2 | PLAGL2 | ENSG00000126003 | Apoptosis |
| NK3 homeobox 1 | NKX3-1 | ENSG00000167034 | Apoptosis |
| pancreatic and duodenal homeobox 1 | PDX1 | ENSG00000139515 | Apoptosis |
| arginine vasopressin | AVP | ENSG00000101200 | Apoptosis |
| bone morphogenetic protein 4 | BMP4 | ENSG00000125378 | Apoptosis |
| interferon alpha inducible protein 27 | IFI27 | ENSG00000275214 | Apoptosis |
| modulator of apoptosis 1 | MOAP1 | ENSG00000278268 | Apoptosis |
| interferon alpha inducible protein 27 like 1 | IFI27L1 | ENSG00000276880 | Apoptosis |
| endoplasmic reticulum oxidoreductase 1 alpha | ERO1A | ENSG00000197930 | Apoptosis |
| interferon alpha inducible protein 27 like 2 | IFI27L2 | ENSG00000276879 | Apoptosis |
| matrix metallopeptidase 9 | MMP9 | ENSG00000100985 | Apoptosis |
| superoxide dismutase 1 | SOD1 | ENSG00000142168 | Apoptosis |
| E2F transcription factor 1 | E2F1 | ENSG00000101412 | Apoptosis |
| transcription factor Dp-1 | TFDP1 | ENSG00000198176 | Apoptosis |
| ectodysplasin A2 receptor | EDA2R | ENSG00000131080 | Apoptosis |
| p21 (RAC1) activated kinase 5 | PAK5 | ENSG00000101349 | Apoptosis |
| MPV17 mitochondrial inner membrane protein like | MPV17L | ENSG00000275543 | Apoptosis |
| cell death inducing p53 target 1 | CDIP1 | ENSG00000089486 | Apoptosis |
| fem-1 homolog B | FEM1B | ENSG00000169018 | Apoptosis |
| sphingosine-1-phosphate phosphatase 1 | SGPP1 | ENSG00000285281 | Apoptosis |
| PML nuclear body scaffold | PML | ENSG00000140464 | Apoptosis |
| protein phosphatase 1 regulatory subunit 13B | PPP1R13B | ENSG00000088808 | Apoptosis |
| tyrosine 3-monooxygenase/tryptophan 5-monooxygenase activation protein epsilon | YWHAE | ENSG00000274474 | Apoptosis |
| msh homeobox 1 | MSX1 | ENSG00000163132 | Apoptosis |
| mitogen-activated protein kinase kinase 5 | MAP2K5 | ENSG00000137764 | Apoptosis |
| cell death inducing p53 target 1 | CDIP1 | ENSG00000274336 | Apoptosis |
| proteasome 26S subunit / non-ATPase 10 | PSMD10 | ENSG00000101843 | Apoptosis |
| GSK3B interacting protein | GSKIP | ENSG00000100744 | Apoptosis |
| death inducer-obliterator 1 | DIDO1 | ENSG00000101191 | Apoptosis |
| tyrosine 3-monooxygenase/tryptophan 5-monooxygenase activation protein zeta | YWHAZ | ENSG00000164924 | Apoptosis |
| siah E3 ubiquitin protein ligase 1 | SIAH1 | ENSG00000196470 | Apoptosis |
| fibroblast growth factor 10 | FGF10 | ENSG00000070193 | Apoptosis |
| DnaJ heat shock protein family (Hsp40) member A1 | DNAJA1 | ENSG00000086061 | Apoptosis |
| MPV17 mitochondrial inner membrane protein like | MPV17L | ENSG00000156968 | Apoptosis |
| Y-box binding protein 3 | YBX3 | ENSG00000060138 | Apoptosis |
| casein kinase 2 alpha 1 | CSNK2A1 | ENSG00000101266 | Apoptosis |
| cullin 4A | CUL4A | ENSG00000139842 | Apoptosis |
| polycomb group ring finger 2 | PCGF2 | ENSG00000278644 | Apoptosis |
| apoptosis antagonizing transcription factor | AATF | ENSG00000276072 | Apoptosis |
| uveal autoantigen with coiled-coil domains and ankyrin repeats | UACA | ENSG00000137831 | Apoptosis |
| CD24 molecule | CD24 | ENSG00000272398 | Apoptosis |
| junction mediating and regulatory protein / p53 cofactor | JMY | ENSG00000152409 | Apoptosis |
| glial cell derived neurotrophic factor | GDNF | ENSG00000168621 | Apoptosis |
| GDNF family receptor alpha like | GFRAL | ENSG00000187871 | Apoptosis |
| microRNA 221 | MIR221 | ENSG00000207870 | Apoptosis |
| microRNA 222 | MIR222 | ENSG00000207725 | Apoptosis |
| EYA transcriptional coactivator and phosphatase 1 | EYA1 | ENSG00000104313 | Apoptosis |
| maternal embryonic leucine zipper kinase | MELK | ENSG00000165304 | Apoptosis |
| BR serine/threonine kinase 2 | BRSK2 | ENSG00000174672 | Apoptosis |
| tumor protein / translationally-controlled 1 | TPT1 | ENSG00000133112 | Apoptosis |
| modulator of apoptosis 1 | MOAP1 | ENSG00000165943 | Apoptosis |
| TCF3 fusion partner | TFPT | ENSG00000274073 | Apoptosis |
| BCL2 like 14 | BCL2L14 | ENSG00000121380 | Apoptosis |
| TCF3 fusion partner | TFPT | ENSG00000273833 | Apoptosis |
| snail family transcriptional repressor 2 | SNAI2 | ENSG00000019549 | Apoptosis |
| TCF3 fusion partner | TFPT | ENSG00000276022 | Apoptosis |
| transmembrane protein 117 | TMEM117 | ENSG00000139173 | Apoptosis |
| DEP domain containing MTOR interacting protein | DEPTOR | ENSG00000155792 | Apoptosis |
| TCF3 fusion partner | TFPT | ENSG00000278161 | Apoptosis |
| TCF3 fusion partner | TFPT | ENSG00000276323 | Apoptosis |
| protein tyrosine phosphatase receptor type C | PTPRC | ENSG00000262418 | Apoptosis |
| TCF3 fusion partner | TFPT | ENSG00000276263 | Apoptosis |
| inositol 1 /4 /5-trisphosphate receptor interacting protein | ITPRIP | ENSG00000148841 | Apoptosis |
| SNW domain containing 1 | SNW1 | ENSG00000100603 | Apoptosis |
| TCF3 fusion partner | TFPT | ENSG00000275086 | Apoptosis |
| apoptosis enhancing nuclease | AEN | ENSG00000181026 | Apoptosis |
| ATPase sarcoplasmic/endoplasmic reticulum Ca2+ transporting 3 | ATP2A3 | ENSG00000074370 | Apoptosis |
| BCL2 associated transcription factor 1 | BCLAF1 | ENSG00000029363 | Apoptosis |
| TCF3 fusion partner | TFPT | ENSG00000276296 | Apoptosis |
| BRCA2 DNA repair associated | BRCA2 | ENSG00000139618 | Apoptosis |
| C-X3-C motif chemokine ligand 1 | CX3CL1 | ENSG00000006210 | Apoptosis |
| serine/threonine kinase 3 | STK3 | ENSG00000104375 | Apoptosis |
| microRNA 17 | MIR17 | ENSG00000284536 | Apoptosis |
| TCF3 fusion partner | TFPT | ENSG00000276504 | Apoptosis |
| coiled-coil-helix-coiled-coil-helix domain containing 10 | CHCHD10 | ENSG00000273607 | Apoptosis |
| bone morphogenetic protein 5 | BMP5 | ENSG00000112175 | Apoptosis |
| nanos C2HC-type zinc finger 3 | NANOS3 | ENSG00000288505 | Apoptosis |
| CCAAT enhancer binding protein beta | CEBPB | ENSG00000172216 | Apoptosis |
| protein tyrosine phosphatase non-receptor type 1 | PTPN1 | ENSG00000196396 | Apoptosis |
| SRC proto-oncogene / non-receptor tyrosine kinase | SRC | ENSG00000197122 | Apoptosis |
| cAMP responsive element binding protein 3 | CREB3 | ENSG00000107175 | Apoptosis |
| B cell receptor associated protein 31 | BCAP31 | ENSG00000185825 | Apoptosis |
| SIVA1 apoptosis inducing factor | SIVA1 | ENSG00000184990 | Apoptosis |
| pro-apoptotic WT1 regulator | PAWR | ENSG00000177425 | Apoptosis |
| transmembrane BAX inhibitor motif containing 6 | TMBIM6 | ENSG00000139644 | Apoptosis |
| E2F transcription factor 2 | E2F2 | ENSG00000282899 | Apoptosis |
| ATP synthase inhibitory factor subunit 1 | ATP5IF1 | ENSG00000285390 | Apoptosis |
| TNF receptor associated protein 1 | TRAP1 | ENSG00000126602 | Apoptosis |
| elongation factor for RNA polymerase II 3 | ELL3 | ENSG00000128886 | Apoptosis |
| TNF receptor associated factor 1 | TRAF1 | ENSG00000056558 | Apoptosis |
| caspase activity and apoptosis inhibitor 1 | CAAP1 | ENSG00000120159 | Apoptosis |
| p53 apoptosis effector related to PMP22 | PERP | ENSG00000112378 | Apoptosis |
| brain derived neurotrophic factor | BDNF | ENSG00000176697 | Apoptosis |
| proline dehydrogenase 1 | PRODH | ENSG00000100033 | Apoptosis |
| G protein subunit alpha i2 | GNAI2 | ENSG00000114353 | Apoptosis |
| NADH:ubiquinone oxidoreductase subunit A13 | NDUFA13 | ENSG00000186010 | Apoptosis |
| syntaxin 4 | STX4 | ENSG00000103496 | Apoptosis |
| heat shock protein family A (Hsp70) member 1A | HSPA1A | ENSG00000237724 | Apoptosis |
| tripartite motif containing 39 | TRIM39 | ENSG00000224994 | Apoptosis |
| BAG cochaperone 6 | BAG6 | ENSG00000229524 | Apoptosis |
| tripartite motif containing 39 | TRIM39 | ENSG00000226437 | Apoptosis |
| secreted frizzled related protein 1 | SFRP1 | ENSG00000104332 | Apoptosis |
| BAG cochaperone 6 | BAG6 | ENSG00000228760 | Apoptosis |
| transmembrane protein 161A | TMEM161A | ENSG00000064545 | Apoptosis |
| corticotropin releasing hormone | CRH | ENSG00000147571 | Apoptosis |
| TATA-box binding protein associated factor 9 | TAF9 | ENSG00000276463 | Apoptosis |
| heat shock protein family A (Hsp70) member 1B | HSPA1B | ENSG00000224501 | Apoptosis |
| hyaluronidase 2 | HYAL2 | ENSG00000068001 | Apoptosis |
| mitogen-activated protein kinase 8 interacting protein 2 | MAPK8IP2 | ENSG00000008735 | Apoptosis |
| serpin family E member 1 | SERPINE1 | ENSG00000106366 | Apoptosis |
| tripartite motif containing 39 | TRIM39 | ENSG00000232839 | Apoptosis |
| heat shock protein family A (Hsp70) member 1A | HSPA1A | ENSG00000234475 | Apoptosis |
| heat shock protein family A (Hsp70) member 1B | HSPA1B | ENSG00000231555 | Apoptosis |
| ribosomal protein S7 | RPS7 | ENSG00000171863 | Apoptosis |
| death domain associated protein | DAXX | ENSG00000227046 | Apoptosis |
| protein phosphatase / Mg2+/Mn2+ dependent 1F | PPM1F | ENSG00000100034 | Apoptosis |
| nuclear factor of activated T cells 4 | NFATC4 | ENSG00000100968 | Apoptosis |
| rhotekin 2 | RTKN2 | ENSG00000182010 | Apoptosis |
| heat shock protein family A (Hsp70) member 1B | HSPA1B | ENSG00000232804 | Apoptosis |
| heat shock protein family A (Hsp70) member 1A | HSPA1A | ENSG00000235941 | Apoptosis |
| BAG cochaperone 6 | BAG6 | ENSG00000227761 | Apoptosis |
| death domain associated protein | DAXX | ENSG00000206206 | Apoptosis |
| tripartite motif containing 39 | TRIM39 | ENSG00000230308 | Apoptosis |
| cullin 5 | CUL5 | ENSG00000166266 | Apoptosis |
| activating transcription factor 4 | ATF4 | ENSG00000128272 | Apoptosis |
| protein phosphatase 2 scaffold subunit Abeta | PPP2R1B | ENSG00000137713 | Apoptosis |
| non-POU domain containing octamer binding | NONO | ENSG00000147140 | Apoptosis |
| frataxin | FXN | ENSG00000165060 | Apoptosis |
| MAGE family member A3 | MAGEA3 | ENSG00000292186 | Apoptosis |
| caspase 8 associated protein 2 | CASP8AP2 | ENSG00000118412 | Apoptosis |
| BAG cochaperone 6 | BAG6 | ENSG00000234651 | Apoptosis |
| death domain associated protein | DAXX | ENSG00000231617 | Apoptosis |
| SH3 domain containing ring finger 1 | SH3RF1 | ENSG00000154447 | Apoptosis |
| armadillo repeat containing 10 | ARMC10 | ENSG00000282813 | Apoptosis |
| folliculin interacting protein 2 | FNIP2 | ENSG00000052795 | Apoptosis |
| tripartite motif containing 39 | TRIM39 | ENSG00000206495 | Apoptosis |
| DAP3 binding cell death enhancer 1 | DELE1 | ENSG00000081791 | Apoptosis |
| BAG cochaperone 6 | BAG6 | ENSG00000096155 | Apoptosis |
| URI1 prefoldin like chaperone | URI1 | ENSG00000105176 | Apoptosis |
| ATPase sarcoplasmic/endoplasmic reticulum Ca2+ transporting 1 | ATP2A1 | ENSG00000196296 | Apoptosis |
| heat shock protein family A (Hsp70) member 1A | HSPA1A | ENSG00000215328 | Apoptosis |
| tripartite motif containing 39 | TRIM39 | ENSG00000229929 | Apoptosis |
| inhibitor of growth family member 2 | ING2 | ENSG00000168556 | Apoptosis |
| plasminogen activator / urokinase receptor | PLAUR | ENSG00000011422 | Apoptosis |
| ATP synthase inhibitory factor subunit 1 | ATP5IF1 | ENSG00000130770 | Apoptosis |
| heat shock protein family A (Hsp70) member 1B | HSPA1B | ENSG00000212866 | Apoptosis |
| TNF receptor superfamily member 12A | TNFRSF12A | ENSG00000006327 | Apoptosis |
| protein kinase C alpha | PRKCA | ENSG00000154229 | Apoptosis |
| death domain associated protein | DAXX | ENSG00000206279 | Apoptosis |
| death domain associated protein | DAXX | ENSG00000229396 | Apoptosis |
| BCL2 like 2 | BCL2L2 | ENSG00000129473 | Apoptosis |
| BAG cochaperone 6 | BAG6 | ENSG00000233348 | Apoptosis |
| EYA transcriptional coactivator and phosphatase 3 | EYA3 | ENSG00000158161 | Apoptosis |
| EPH receptor A2 | EPHA2 | ENSG00000142627 | Apoptosis |
| TNF receptor superfamily member 1B | TNFRSF1B | ENSG00000028137 | Apoptosis |
| ubiquitination factor E4B | UBE4B | ENSG00000130939 | Apoptosis |
| angiotensin II receptor type 2 | AGTR2 | ENSG00000180772 | Apoptosis |
| protein phosphatase 3 catalytic subunit gamma | PPP3CC | ENSG00000120910 | Apoptosis |
| microRNA 27b | MIR27B | ENSG00000207864 | Apoptosis |
| acetyl-CoA acyltransferase 2 | ACAA2 | ENSG00000167315 | Apoptosis |
| KIT ligand | KITLG | ENSG00000049130 | Apoptosis |
| ribosomal protein S3 | RPS3 | ENSG00000149273 | Apoptosis |
| armadillo repeat containing 10 | ARMC10 | ENSG00000170632 | Apoptosis |
| tripartite motif containing 32 | TRIM32 | ENSG00000119401 | Apoptosis |
| poly(ADP-ribose) polymerase 2 | PARP2 | ENSG00000129484 | Apoptosis |
| adaptor protein / phosphotyrosine interacting with PH domain and leucine zipper 1 | APPL1 | ENSG00000157500 | Apoptosis |
| glutamate ionotropic receptor NMDA type subunit associated protein 1 | GRINA | ENSG00000178719 | Apoptosis |
| coiled-coil-helix-coiled-coil-helix domain containing 10 | CHCHD10 | ENSG00000250479 | Apoptosis |
| bradykinin receptor B2 | BDKRB2 | ENSG00000168398 | Apoptosis |
| thioredoxin domain containing 12 | TXNDC12 | ENSG00000117862 | Apoptosis |
| translocase of inner mitochondrial membrane 50 | TIMM50 | ENSG00000105197 | Apoptosis |
| interleukin 2 | IL2 | ENSG00000109471 | Apoptosis |
| microRNA 449a | MIR449A | ENSG00000198983 | Apoptosis |
| integrin subunit alpha M | ITGAM | ENSG00000169896 | Apoptosis |
| CD70 molecule | CD70 | ENSG00000125726 | Apoptosis |
| TATA-box binding protein associated factor 9b | TAF9B | ENSG00000187325 | Apoptosis |
| high mobility group box 2 | HMGB2 | ENSG00000164104 | Apoptosis |
| NCK adaptor protein 1 | NCK1 | ENSG00000158092 | Apoptosis |
| homeodomain interacting protein kinase 2 | HIPK2 | ENSG00000064393 | Apoptosis |
| fission / mitochondrial 1 | FIS1 | ENSG00000214253 | Apoptosis |
| CD38 molecule | CD38 | ENSG00000004468 | Apoptosis |
| siah E3 ubiquitin protein ligase 2 | SIAH2 | ENSG00000181788 | Apoptosis |
| galectin 3 | LGALS3 | ENSG00000131981 | Apoptosis |
| DCC netrin 1 receptor | DCC | ENSG00000187323 | Apoptosis |
| DNA damage inducible transcript 4 | DDIT4 | ENSG00000168209 | Apoptosis |
| peptidylprolyl isomerase F | PPIF | ENSG00000108179 | Apoptosis |
| TOP1 binding arginine/serine rich protein / E3 ubiquitin ligase | TOPORS | ENSG00000197579 | Apoptosis |
| CASP2 and RIPK1 domain containing adaptor with death domain | CRADD | ENSG00000169372 | Apoptosis |
| tyrosine 3-monooxygenase/tryptophan 5-monooxygenase activation protein beta | YWHAB | ENSG00000166913 | Apoptosis |
| serine/threonine kinase 4 | STK4 | ENSG00000101109 | Apoptosis |
| DNA polymerase beta | POLB | ENSG00000070501 | Apoptosis |
| BCL2 related protein A1 | BCL2A1 | ENSG00000140379 | Apoptosis |
| NCK adaptor protein 2 | NCK2 | ENSG00000071051 | Apoptosis |
| sphingomyelin synthase 1 | SGMS1 | ENSG00000198964 | Apoptosis |
| TIR domain containing adaptor molecule 2 | TICAM2 | ENSG00000243414 | Apoptosis |
| zinc finger C3HC-type containing 1 | ZC3HC1 | ENSG00000091732 | Apoptosis |
| histidine triad nucleotide binding protein 1 | HINT1 | ENSG00000169567 | Apoptosis |
| selenoprotein S | SELENOS | ENSG00000131871 | Apoptosis |
| ubiquitin specific peptidase 47 | USP47 | ENSG00000170242 | Apoptosis |
| ERCC excision repair 6 / chromatin remodeling factor | ERCC6 | ENSG00000225830 | Apoptosis |
| phospholipid scramblase 3 | PLSCR3 | ENSG00000187838 | Apoptosis |
| MAGE family member A3 | MAGEA3 | ENSG00000221867 | Apoptosis |
| transmembrane protein 102 | TMEM102 | ENSG00000181284 | Apoptosis |
| heat shock protein family A (Hsp70) member 1A | HSPA1A | ENSG00000204389 | Apoptosis |
| heat shock protein family A (Hsp70) member 1B | HSPA1B | ENSG00000204388 | Apoptosis |
| purinergic receptor P2X 7 | P2RX7 | ENSG00000089041 | Apoptosis |
| ribosomal protein L26 | RPL26 | ENSG00000161970 | Apoptosis |
| somatostatin | SST | ENSG00000157005 | Apoptosis |
| zinc finger protein 205 | ZNF205 | ENSG00000122386 | Apoptosis |
| mitogen-activated protein kinase 8 interacting protein 1 | MAPK8IP1 | ENSG00000121653 | Apoptosis |
| nitric oxide synthase 3 | NOS3 | ENSG00000164867 | Apoptosis |
| cAMP responsive element binding protein 3 like 1 | CREB3L1 | ENSG00000157613 | Apoptosis |
| inhibin subunit beta B | INHBB | ENSG00000163083 | Apoptosis |
| zinc finger protein 385B | ZNF385B | ENSG00000144331 | Apoptosis |
| retrotransposon Gag like 10 | RTL10 | ENSG00000215012 | Apoptosis |
| gamma-glutamylcyclotransferase | GGCT | ENSG00000006625 | Apoptosis |
| interferon alpha inducible protein 27 like 1 | IFI27L1 | ENSG00000165948 | Apoptosis |
| interferon alpha inducible protein 27 like 2 | IFI27L2 | ENSG00000119632 | Apoptosis |
| zinc finger SWIM-type containing 2 | ZSWIM2 | ENSG00000163012 | Apoptosis |
| N-acylsphingosine amidohydrolase 2 | ASAH2 | ENSG00000188611 | Apoptosis |
| cysteine rich protein 1 | CRIP1 | ENSG00000213145 | Apoptosis |
| homocysteine inducible ER protein with ubiquitin like domain 1 | HERPUD1 | ENSG00000051108 | Apoptosis |
| cytochrome c oxidase assembly factor 8 | COA8 | ENSG00000256053 | Apoptosis |
| TIMP metallopeptidase inhibitor 3 | TIMP3 | ENSG00000100234 | Apoptosis |
| tyrosine 3-monooxygenase/tryptophan 5-monooxygenase activation protein gamma | YWHAG | ENSG00000170027 | Apoptosis |
| ring finger protein 183 | RNF183 | ENSG00000165188 | Apoptosis |
| BCL2 like 10 | BCL2L10 | ENSG00000137875 | Apoptosis |
| interferon beta 1 | IFNB1 | ENSG00000171855 | Apoptosis |
| dual specificity tyrosine phosphorylation regulated kinase 2 | DYRK2 | ENSG00000127334 | Apoptosis |
| checkpoint kinase 2 | CHEK2 | ENSG00000183765 | Apoptosis |
| mitogen-activated protein kinase 7 | MAPK7 | ENSG00000166484 | Apoptosis |
| TNF receptor superfamily member 10c | TNFRSF10C | ENSG00000173535 | Apoptosis |
| POU class 4 homeobox 2 | POU4F2 | ENSG00000151615 | Apoptosis |
| PIH1 domain containing 1 | PIH1D1 | ENSG00000104872 | Apoptosis |
| integral membrane protein 2C | ITM2C | ENSG00000135916 | Apoptosis |
| peroxisome proliferator activated receptor delta | PPARD | ENSG00000112033 | Apoptosis |
| MAX network transcriptional repressor | MNT | ENSG00000070444 | Apoptosis |
| atypical chemokine receptor 3 | ACKR3 | ENSG00000144476 | Apoptosis |
| protein phosphatase 3 regulatory subunit B / alpha | PPP3R1 | ENSG00000221823 | Apoptosis |
| SP100 nuclear antigen | SP100 | ENSG00000067066 | Apoptosis |
| TP53 regulated inhibitor of apoptosis 1 | TRIAP1 | ENSG00000170855 | Apoptosis |
| solute carrier family 25 member 5 | SLC25A5 | ENSG00000005022 | Apoptosis |
| mitogen-activated protein kinase 9 | MAPK9 | ENSG00000050748 | Apoptosis |
| galectin 12 | LGALS12 | ENSG00000133317 | Apoptosis |
| caveolin 1 | CAV1 | ENSG00000105974 | Apoptosis |
| microRNA 210 | MIR210 | ENSG00000199038 | Apoptosis |
| lymphocyte antigen 96 | LY96 | ENSG00000154589 | Apoptosis |
| influenza virus NS1A binding protein | IVNS1ABP | ENSG00000116679 | Apoptosis |
| membrane associated ring-CH-type finger 7 | MARCHF7 | ENSG00000136536 | Apoptosis |
| growth hormone inducible transmembrane protein | GHITM | ENSG00000165678 | Apoptosis |
| serine/threonine kinase 25 | STK25 | ENSG00000115694 | Apoptosis |
| protein phosphatase 1 catalytic subunit alpha | PPP1CA | ENSG00000172531 | Apoptosis |
| inhibin subunit beta A | INHBA | ENSG00000122641 | Apoptosis |
| microRNA 132 | MIR132 | ENSG00000267200 | Apoptosis |
| HIC ZBTB transcriptional repressor 1 | HIC1 | ENSG00000177374 | Apoptosis |
| cell cycle and apoptosis regulator 2 | CCAR2 | ENSG00000158941 | Apoptosis |
| peroxiredoxin 2 | PRDX2 | ENSG00000167815 | Apoptosis |
| erythropoietin | EPO | ENSG00000130427 | Apoptosis |
| F-box DNA helicase 1 | FBH1 | ENSG00000134452 | Apoptosis |
| transforming growth factor beta 2 | TGFB2 | ENSG00000092969 | Apoptosis |
| GATA binding protein 1 | GATA1 | ENSG00000102145 | Apoptosis |
| DEAD-box helicase 5 | DDX5 | ENSG00000108654 | Apoptosis |
| uromodulin | UMOD | ENSG00000169344 | Apoptosis |
| transmembrane immune signaling adaptor TYROBP | TYROBP | ENSG00000011600 | Apoptosis |
| NADH:ubiquinone oxidoreductase core subunit S3 | NDUFS3 | ENSG00000213619 | Apoptosis |
| protein tyrosine phosphatase mitochondrial 1 | PTPMT1 | ENSG00000110536 | Apoptosis |
| N-myristoyltransferase 1 | NMT1 | ENSG00000136448 | Apoptosis |
| collagen type II alpha 1 chain | COL2A1 | ENSG00000139219 | Apoptosis |
| zinc finger protein 622 | ZNF622 | ENSG00000173545 | Apoptosis |
| protein disulfide isomerase family A member 3 | PDIA3 | ENSG00000167004 | Apoptosis |
| transforming growth factor beta 1 | TGFB1 | ENSG00000105329 | Apoptosis |
| cyclin dependent kinase inhibitor 2D | CDKN2D | ENSG00000129355 | Apoptosis |
| MYB binding protein 1a | MYBBP1A | ENSG00000132382 | Apoptosis |
| arrestin beta 2 | ARRB2 | ENSG00000141480 | Apoptosis |
| transcription factor Dp-2 | TFDP2 | ENSG00000114126 | Apoptosis |
| calcium and integrin binding 1 | CIB1 | ENSG00000185043 | Apoptosis |
| ribosomal protein S27 like | RPS27L | ENSG00000185088 | Apoptosis |
| Fas cell surface death receptor | FAS | ENSG00000026103 | Apoptosis |
| cell death inducing DFFA like effector b | CIDEB | ENSG00000136305 | Apoptosis |
| death effector domain containing 2 | DEDD2 | ENSG00000160570 | Apoptosis |
| interleukin 20 receptor subunit alpha | IL20RA | ENSG00000016402 | Apoptosis |
| nibrin | NBN | ENSG00000104320 | Apoptosis |
| maelstrom spermatogenic transposon silencer | MAEL | ENSG00000143194 | Apoptosis |
| protein kinase / DNA-activated / catalytic subunit | PRKDC | ENSG00000253729 | Apoptosis |
| unc-5 netrin receptor B | UNC5B | ENSG00000107731 | Apoptosis |
| transmembrane protein 109 | TMEM109 | ENSG00000110108 | Apoptosis |
| zinc finger protein 385A | ZNF385A | ENSG00000161642 | Apoptosis |
| sphingosine-1-phosphate lyase 1 | SGPL1 | ENSG00000166224 | Apoptosis |
| pyruvate dehydrogenase kinase 1 | PDK1 | ENSG00000152256 | Apoptosis |
| mitochondrial E3 ubiquitin protein ligase 1 | MUL1 | ENSG00000090432 | Apoptosis |
| suppressor of tumorigenicity 20 | ST20 | ENSG00000180953 | Apoptosis |
| cystathionine gamma-lyase | CTH | ENSG00000116761 | Apoptosis |
| E2F transcription factor 2 | E2F2 | ENSG00000007968 | Apoptosis |
| interferon alpha inducible protein 27 | IFI27 | ENSG00000165949 | Apoptosis |
| ribosomal protein L11 | RPL11 | ENSG00000142676 | Apoptosis |
| inhibitor of growth family member 5 | ING5 | ENSG00000168395 | Apoptosis |
| endoplasmic reticulum to nucleus signaling 2 | ERN2 | ENSG00000134398 | Apoptosis |
| receptor for activated C kinase 1 | RACK1 | ENSG00000204628 | Apoptosis |
| DNA damage induced apoptosis suppressor | DDIAS | ENSG00000165490 | Apoptosis |
| caspase 2 | CASP2 | ENSG00000106144 | Apoptosis |
| programmed cell death 6 | PDCD6 | ENSG00000249915 | Apoptosis |
| DAB2 interacting protein | DAB2IP | ENSG00000136848 | Apoptosis |
| microRNA 198 | MIR198 | ENSG00000284121 | Apoptosis |
| BAG cochaperone 5 | BAG5 | ENSG00000166170 | Apoptosis |
| programmed cell death 10 | PDCD10 | ENSG00000114209 | Apoptosis |
| PRELI domain containing 1 | PRELID1 | ENSG00000169230 | Apoptosis |
| dopamine beta-hydroxylase | DBH | ENSG00000123454 | Apoptosis |
| ribosomal RNA processing 8 | RRP8 | ENSG00000132275 | Apoptosis |
| apoptosis antagonizing transcription factor | AATF | ENSG00000275700 | Apoptosis |
| huntingtin interacting protein 1 related | HIP1R | ENSG00000130787 | Apoptosis |
| activin A receptor type 1B | ACVR1B | ENSG00000135503 | Apoptosis |
| platelet factor 4 | PF4 | ENSG00000163737 | Apoptosis |
| death associated protein 3 | DAP3 | ENSG00000132676 | Apoptosis |
| harakiri / BCL2 interacting protein | HRK | ENSG00000135116 | Apoptosis |
| lymphotoxin beta receptor | LTBR | ENSG00000111321 | Apoptosis |
| zinc finger MYND-type containing 11 | ZMYND11 | ENSG00000015171 | Apoptosis |
| p21 (RAC1) activated kinase 2 | PAK2 | ENSG00000180370 | Apoptosis |
| TATA-box binding protein associated factor 9 | TAF9 | ENSG00000273841 | Apoptosis |
| prolyl 4-hydroxylase subunit beta | P4HB | ENSG00000185624 | Apoptosis |
| CD5 molecule | CD5 | ENSG00000110448 | Apoptosis |
| glutaminyl-tRNA synthetase 1 | QARS1 | ENSG00000172053 | Apoptosis |
| transcription factor 7 like 2 | TCF7L2 | ENSG00000148737 | Apoptosis |
| ring finger and FYVE like domain containing E3 ubiquitin protein ligase | RFFL | ENSG00000092871 | Apoptosis |
| DEAD-box helicase 3 X-linked | DDX3X | ENSG00000215301 | Apoptosis |
| sonic hedgehog signaling molecule | SHH | ENSG00000164690 | Apoptosis |
| notchless homolog 1 | NLE1 | ENSG00000073536 | Apoptosis |
| proliferation and apoptosis adaptor protein 15 | PEA15 | ENSG00000162734 | Apoptosis |
| NME/NM23 family member 5 | NME5 | ENSG00000112981 | Apoptosis |
| CD74 molecule | CD74 | ENSG00000019582 | Apoptosis |
| fibroblast growth factor receptor 1 | FGFR1 | ENSG00000077782 | Apoptosis |
| frizzled class receptor 9 | FZD9 | ENSG00000188763 | Apoptosis |
| presenilin 1 | PSEN1 | ENSG00000080815 | Apoptosis |
| CD14 molecule | CD14 | ENSG00000170458 | Apoptosis |
| ras homolog family member T1 | RHOT1 | ENSG00000126858 | Apoptosis |
| heterogeneous nuclear ribonucleoprotein K | HNRNPK | ENSG00000165119 | Apoptosis |
| MAPK interacting serine/threonine kinase 2 | MKNK2 | ENSG00000099875 | Apoptosis |
| tyrosine 3-monooxygenase/tryptophan 5-monooxygenase activation protein epsilon | YWHAE | ENSG00000108953 | Apoptosis |
| TCF3 fusion partner | TFPT | ENSG00000105619 | Apoptosis |
| OPA1 mitochondrial dynamin like GTPase | OPA1 | ENSG00000198836 | Apoptosis |
| sphingosine-1-phosphate phosphatase 1 | SGPP1 | ENSG00000126821 | Apoptosis |
| glutathione S-transferase pi 1 | GSTP1 | ENSG00000084207 | Apoptosis |
| diablo IAP-binding mitochondrial protein | DIABLO | ENSG00000184047 | Apoptosis |
| huntingtin interacting protein 1 | HIP1 | ENSG00000127946 | Apoptosis |
| BAG cochaperone 6 | BAG6 | ENSG00000204463 | Apoptosis |
| serine/threonine/tyrosine interacting like 1 | STYXL1 | ENSG00000127952 | Apoptosis |
| phosphoinositide-3-kinase regulatory subunit 1 | PIK3R1 | ENSG00000145675 | Apoptosis |
| CD27 molecule | CD27 | ENSG00000139193 | Apoptosis |
| cyclin dependent kinase inhibitor 1A | CDKN1A | ENSG00000124762 | Apoptosis |
| vanin 1 | VNN1 | ENSG00000112299 | Apoptosis |
| pyruvate dehydrogenase kinase 2 | PDK2 | ENSG00000005882 | Apoptosis |
| Fas activated serine/threonine kinase | FASTK | ENSG00000164896 | Apoptosis |
| cullin 1 | CUL1 | ENSG00000055130 | Apoptosis |
| NACC family member 2 | NACC2 | ENSG00000148411 | Apoptosis |
| interleukin 7 | IL7 | ENSG00000104432 | Apoptosis |
| protein activator of interferon induced protein kinase EIF2AK2 | PRKRA | ENSG00000180228 | Apoptosis |
| thrombospondin 1 | THBS1 | ENSG00000137801 | Apoptosis |
| G protein-coupled estrogen receptor 1 | GPER1 | ENSG00000164850 | Apoptosis |
| cullin 2 | CUL2 | ENSG00000108094 | Apoptosis |
| GABA type A receptor-associated protein | GABARAP | ENSG00000170296 | Apoptosis |
| transmembrane protein 14A | TMEM14A | ENSG00000096092 | Apoptosis |
| mitogen-activated protein kinase kinase kinase 5 | MAP3K5 | ENSG00000197442 | Apoptosis |
| SMAD family member 3 | SMAD3 | ENSG00000166949 | Apoptosis |
| protein inhibitor of activated STAT 4 | PIAS4 | ENSG00000105229 | Apoptosis |
| tripartite motif containing 39 | TRIM39 | ENSG00000204599 | Apoptosis |
| septin 4 | SEPTIN4 | ENSG00000108387 | Apoptosis |
| transforming growth factor beta receptor 1 | TGFBR1 | ENSG00000106799 | Apoptosis |
| pleckstrin homology domain interacting protein | PHIP | ENSG00000146247 | Apoptosis |
| phosphatase and tensin homolog | PTEN | ENSG00000171862 | Apoptosis |
| ras homolog family member T2 | RHOT2 | ENSG00000140983 | Apoptosis |
| p53-induced death domain protein 1 | PIDD1 | ENSG00000177595 | Apoptosis |
| TNF receptor superfamily member 25 | TNFRSF25 | ENSG00000215788 | Apoptosis |
| annexin A6 | ANXA6 | ENSG00000197043 | Apoptosis |
| telomerase reverse transcriptase | TERT | ENSG00000164362 | Apoptosis |
| C-X-C motif chemokine ligand 12 | CXCL12 | ENSG00000107562 | Apoptosis |
| superoxide dismutase 2 | SOD2 | ENSG00000291237 | Apoptosis |
| death domain associated protein | DAXX | ENSG00000204209 | Apoptosis |
| ring finger protein 186 | RNF186 | ENSG00000178828 | Apoptosis |
| WD repeat domain 35 | WDR35 | ENSG00000118965 | Apoptosis |
| CD3 epsilon subunit of T-cell receptor complex | CD3E | ENSG00000198851 | Apoptosis |
| nanos C2HC-type zinc finger 3 | NANOS3 | ENSG00000187556 | Apoptosis |
| apoptotic peptidase activating factor 1 | APAF1 | ENSG00000120868 | Apoptosis |
| ubiquitin B | UBB | ENSG00000170315 | Apoptosis |
| DnaJ heat shock protein family (Hsp40) member C10 | DNAJC10 | ENSG00000077232 | Apoptosis |
| EYA transcriptional coactivator and phosphatase 4 | EYA4 | ENSG00000112319 | Apoptosis |
| DEAD-box helicase 47 | DDX47 | ENSG00000213782 | Apoptosis |
| TNF receptor associated factor 7 | TRAF7 | ENSG00000131653 | Apoptosis |
| FYN proto-oncogene / Src family tyrosine kinase | FYN | ENSG00000010810 | Apoptosis |
| erb-b2 receptor tyrosine kinase 3 | ERBB3 | ENSG00000065361 | Apoptosis |
| endoplasmic reticulum protein 29 | ERP29 | ENSG00000089248 | Apoptosis |
| proteasome activator subunit 3 | PSME3 | ENSG00000131467 | Apoptosis |
| STE20 related adaptor beta | STRADB | ENSG00000082146 | Apoptosis |
| histone deacetylase 1 | HDAC1 | ENSG00000116478 | Apoptosis |
| fibrinogen beta chain | FGB | ENSG00000171564 | Apoptosis |
| fibrinogen alpha chain | FGA | ENSG00000171560 | Apoptosis |
| fibrinogen gamma chain | FGG | ENSG00000171557 | Apoptosis |
| MYC associated zinc finger protein | MAZ | ENSG00000103495 | Apoptosis |
| fibroblast growth factor receptor 3 | FGFR3 | ENSG00000068078 | Apoptosis |
| ubiquitin conjugating enzyme E2 K | UBE2K | ENSG00000078140 | Apoptosis |
| programmed cell death 5 | PDCD5 | ENSG00000105185 | Apoptosis |
| death effector domain containing | DEDD | ENSG00000158796 | Apoptosis |
| polycomb group ring finger 2 | PCGF2 | ENSG00000277258 | Apoptosis |
| keratin 18 | KRT18 | ENSG00000111057 | Apoptosis |
| keratin 8 | KRT8 | ENSG00000170421 | Apoptosis |
| nucleolar protein 3 | NOL3 | ENSG00000140939 | Apoptosis |
| WW domain containing oxidoreductase | WWOX | ENSG00000186153 | Apoptosis |
| inhibitor of CDK / cyclin A1 interacting protein 1 | INCA1 | ENSG00000196388 | Apoptosis |
| bone morphogenetic protein receptor type 1B | BMPR1B | ENSG00000138696 | Apoptosis |
| purinergic receptor P2X 4 | P2RX4 | ENSG00000135124 | Apoptosis |
| BCL2 like 12 | BCL2L12 | ENSG00000126453 | Apoptosis |
| MDM2 proto-oncogene | MDM2 | ENSG00000135679 | Apoptosis |
| TPD52 like 1 | TPD52L1 | ENSG00000111907 | Apoptosis |
| activin A receptor type 1 | ACVR1 | ENSG00000115170 | Apoptosis |
| interferon alpha inducible protein 6 | IFI6 | ENSG00000126709 | Apoptosis |
| selenoprotein K | SELENOK | ENSG00000113811 | Apoptosis |
| nerve growth factor | NGF | ENSG00000134259 | Apoptosis |
| NOC2 like nucleolar associated transcriptional repressor | NOC2L | ENSG00000188976 | Apoptosis |
| shisa family member 5 | SHISA5 | ENSG00000164054 | Apoptosis |
| secreted frizzled related protein 2 | SFRP2 | ENSG00000145423 | Apoptosis |
| sodium voltage-gated channel alpha subunit 2 | SCN2A | ENSG00000136531 | Apoptosis |
| eukaryotic translation initiation factor 2 alpha kinase 3 | EIF2AK3 | ENSG00000172071 | Apoptosis |
| BCL2 binding component 3 | BBC3 | ENSG00000105327 | Apoptosis |
| transmembrane channel like 8 | TMC8 | ENSG00000167895 | Apoptosis |
| hypoxia up-regulated 1 | HYOU1 | ENSG00000149428 | Apoptosis |
| ring finger protein 34 | RNF34 | ENSG00000170633 | Apoptosis |
| TNF superfamily member 12 | TNFSF12 | ENSG00000239697 | Apoptosis |
| insulin | INS | ENSG00000254647 | Apoptosis |
| noggin | NOG | ENSG00000183691 | Apoptosis |
| family with sequence similarity 162 member A | FAM162A | ENSG00000114023 | Apoptosis |
| fidgetin like 1 | FIGNL1 | ENSG00000132436 | Apoptosis |
| synoviolin 1 | SYVN1 | ENSG00000162298 | Apoptosis |
| Fas apoptotic inhibitory molecule | FAIM | ENSG00000158234 | Apoptosis |
| protein phosphatase 1 regulatory subunit 15A | PPP1R15A | ENSG00000087074 | Apoptosis |
| ubiquitin specific peptidase 28 | USP28 | ENSG00000048028 | Apoptosis |
| BCL3 transcription coactivator | BCL3 | ENSG00000069399 | Apoptosis |
| cullin 3 | CUL3 | ENSG00000036257 | Apoptosis |
| protein kinase C delta | PRKCD | ENSG00000163932 | Apoptosis |
| C-X3-C motif chemokine receptor 1 | CX3CR1 | ENSG00000168329 | Apoptosis |
| caspase 12 (gene/pseudogene) | CASP12 | ENSG00000204403 | Apoptosis |
| parathyroid hormone | PTH | ENSG00000152266 | Apoptosis |
| double PHD fingers 2 | DPF2 | ENSG00000133884 | Apoptosis |
| cholecystokinin | CCK | ENSG00000187094 | Apoptosis |
| insulin like growth factor 1 | IGF1 | ENSG00000017427 | Apoptosis |
| MLLT11 transcription factor 7 cofactor | MLLT11 | ENSG00000213190 | Apoptosis |
| transmembrane BAX inhibitor motif containing 1 | TMBIM1 | ENSG00000135926 | Apoptosis |
| microRNA 26b | MIR26B | ENSG00000199121 | Apoptosis |
| Wnt family member 4 | WNT4 | ENSG00000162552 | Apoptosis |
| inhibitor of nuclear factor kappa B kinase subunit epsilon | IKBKE | ENSG00000263528 | Apoptosis |
| SUMO specific peptidase 1 | SENP1 | ENSG00000079387 | Apoptosis |
| Raf-1 proto-oncogene / serine/threonine kinase | RAF1 | ENSG00000132155 | Apoptosis |
| integrin subunit alpha 6 | ITGA6 | ENSG00000091409 | Apoptosis |
| interleukin 12A | IL12A | ENSG00000168811 | Apoptosis |
| lysine demethylase 1A | KDM1A | ENSG00000004487 | Apoptosis |
| Fas apoptotic inhibitory molecule 2 | FAIM2 | ENSG00000135472 | Apoptosis |
| MAP kinase activating death domain | MADD | ENSG00000110514 | Apoptosis |
| CD28 molecule | CD28 | ENSG00000178562 | Apoptosis |
| caspase 10 | CASP10 | ENSG00000003400 | Apoptosis |
| pellino E3 ubiquitin protein ligase family member 3 | PELI3 | ENSG00000174516 | Apoptosis |
| stratifin | SFN | ENSG00000175793 | Apoptosis |
| glutathione peroxidase 1 | GPX1 | ENSG00000233276 | Apoptosis |
| integrin subunit alpha V | ITGAV | ENSG00000138448 | Apoptosis |
| nerve growth factor receptor | NGFR | ENSG00000064300 | Apoptosis |
| Yes1 associated transcriptional regulator | YAP1 | ENSG00000137693 | Apoptosis |
| BRCA1 DNA repair associated | BRCA1 | ENSG00000012048 | Apoptosis |
| zinc finger DHHC-type palmitoyltransferase 3 | ZDHHC3 | ENSG00000163812 | Apoptosis |
| mucin 1 / cell surface associated | MUC1 | ENSG00000185499 | Apoptosis |
| microRNA 21 | MIR21 | ENSG00000284190 | Apoptosis |
| ribosomal protein S6 kinase B1 | RPS6KB1 | ENSG00000108443 | Apoptosis |
| HRas proto-oncogene / GTPase | HRAS | ENSG00000174775 | Apoptosis |
| interleukin 19 | IL19 | ENSG00000142224 | Apoptosis |
| SKI like proto-oncogene | SKIL | ENSG00000136603 | Apoptosis |
| pleckstrin homology like domain family A member 3 | PHLDA3 | ENSG00000174307 | Apoptosis |
| fragile histidine triad diadenosine triphosphatase | FHIT | ENSG00000189283 | Apoptosis |
| ADP ribosylation factor like GTPase 6 interacting protein 5 | ARL6IP5 | ENSG00000144746 | Apoptosis |
| tyrosine 3-monooxygenase/tryptophan 5-monooxygenase activation protein theta | YWHAQ | ENSG00000134308 | Apoptosis |
| secretogranin II | SCG2 | ENSG00000171951 | Apoptosis |
| mutL homolog 1 | MLH1 | ENSG00000076242 | Apoptosis |
| nuclear receptor subfamily 4 group A member 2 | NR4A2 | ENSG00000153234 | Apoptosis |
| baculoviral IAP repeat containing 6 | BIRC6 | ENSG00000115760 | Apoptosis |
| G0/G1 switch 2 | G0S2 | ENSG00000123689 | Apoptosis |
| splicing factor proline and glutamine rich | SFPQ | ENSG00000116560 | Apoptosis |
| Rho/Rac guanine nucleotide exchange factor 2 | ARHGEF2 | ENSG00000116584 | Apoptosis |
| mitochondrial fission factor | MFF | ENSG00000168958 | Apoptosis |
| angiotensinogen | AGT | ENSG00000135744 | Apoptosis |
| LCK proto-oncogene / Src family tyrosine kinase | LCK | ENSG00000182866 | Apoptosis |
| tumor protein p73 | TP73 | ENSG00000078900 | Apoptosis |
| homeodomain interacting protein kinase 1 | HIPK1 | ENSG00000163349 | Apoptosis |
| solute carrier family 35 member F6 | SLC35F6 | ENSG00000213699 | Apoptosis |
| cytochrome P450 family 1 subfamily B member 1 | CYP1B1 | ENSG00000138061 | Apoptosis |
| protein tyrosine phosphatase receptor type C | PTPRC | ENSG00000081237 | Apoptosis |
| tumor protein p53 binding protein 2 | TP53BP2 | ENSG00000143514 | Apoptosis |
| mutS homolog 2 | MSH2 | ENSG00000095002 | Apoptosis |
| mutS homolog 6 | MSH6 | ENSG00000116062 | Apoptosis |
| S100 calcium binding protein A9 | S100A9 | ENSG00000163220 | Apoptosis |
| S100 calcium binding protein A8 | S100A8 | ENSG00000143546 | Apoptosis |
| interleukin 6 receptor | IL6R | ENSG00000160712 | Apoptosis |
| Fas ligand | FASLG | ENSG00000117560 | Apoptosis |
| BCL10 immune signaling adaptor | BCL10 | ENSG00000142867 | Apoptosis |
| enolase 1 | ENO1 | ENSG00000074800 | Apoptosis |
| adenosine A1 receptor | ADORA1 | ENSG00000163485 | Apoptosis |
| Jun proto-oncogene / AP-1 transcription factor subunit | JUN | ENSG00000177606 | Apoptosis |
| TM2 domain containing 1 | TM2D1 | ENSG00000162604 | Apoptosis |
| activating transcription factor 3 | ATF3 | ENSG00000162772 | Apoptosis |
| RELA proto-oncogene / NF-kB subunit | RELA | ENSG00000173039 | Apoptosis/Alkaliptosis |
| cortactin | CTTN | ENSG00000288401 | Apoptosis/Autophagy |
| protein tyrosine phosphatase non-receptor type 2 | PTPN2 | ENSG00000175354 | Apoptosis/Autophagy |
| RB transcriptional corepressor 1 | RB1 | ENSG00000139687 | Apoptosis/Autophagy |
| GATA binding protein 4 | GATA4 | ENSG00000285109 | Apoptosis/Autophagy |
| Bcl2 modifying factor | BMF | ENSG00000104081 | Apoptosis/Autophagy |
| BCL2 interacting protein 3 like | BNIP3L | ENSG00000104765 | Apoptosis/Autophagy |
| BCL2 like 1 | BCL2L1 | ENSG00000171552 | Apoptosis/Autophagy |
| ubiquilin 1 | UBQLN1 | ENSG00000135018 | Apoptosis/Autophagy |
| death associated protein kinase 1 | DAPK1 | ENSG00000196730 | Apoptosis/Autophagy |
| RB1 inducible coiled-coil 1 | RB1CC1 | ENSG00000023287 | Apoptosis/Autophagy |
| tribbles pseudokinase 3 | TRIB3 | ENSG00000101255 | Apoptosis/Autophagy |
| hepatocyte growth factor | HGF | ENSG00000019991 | Apoptosis/Autophagy |
| BAG cochaperone 3 | BAG3 | ENSG00000151929 | Apoptosis/Autophagy |
| regulator of MON1-CCZ1 | RMC1 | ENSG00000141452 | Apoptosis/Autophagy |
| E1A binding protein p300 | EP300 | ENSG00000100393 | Apoptosis/Autophagy |
| intercellular adhesion molecule 1 | ICAM1 | ENSG00000090339 | Apoptosis/Autophagy |
| hypoxia inducible factor 1 subunit alpha | HIF1A | ENSG00000100644 | Apoptosis/Autophagy |
| ret proto-oncogene | RET | ENSG00000165731 | Apoptosis/Autophagy |
| sirtuin 1 | SIRT1 | ENSG00000096717 | Apoptosis/Autophagy |
| BCL2 interacting protein 3 | BNIP3 | ENSG00000176171 | Apoptosis/Autophagy |
| GATA binding protein 4 | GATA4 | ENSG00000136574 | Apoptosis/Autophagy |
| BCL2 family apoptosis regulator BOK | BOK | ENSG00000176720 | Apoptosis/Autophagy |
| casein kinase 2 alpha 2 | CSNK2A2 | ENSG00000070770 | Apoptosis/Autophagy |
| mitogen-activated protein kinase 8 | MAPK8 | ENSG00000107643 | Apoptosis/Autophagy |
| nuclear protein 1 / transcriptional regulator | NUPR1 | ENSG00000176046 | Apoptosis/Autophagy |
| BCL2 associated agonist of cell death | BAD | ENSG00000002330 | Apoptosis/Autophagy |
| huntingtin | HTT | ENSG00000197386 | Apoptosis/Autophagy |
| glycogen synthase kinase 3 alpha | GSK3A | ENSG00000105723 | Apoptosis/Autophagy |
| death associated protein like 1 | DAPL1 | ENSG00000163331 | Apoptosis/Autophagy |
| PTEN induced kinase 1 | PINK1 | ENSG00000158828 | Apoptosis/Autophagy |
| cortactin | CTTN | ENSG00000085733 | Apoptosis/Autophagy |
| death associated protein kinase 2 | DAPK2 | ENSG00000035664 | Apoptosis/Autophagy |
| endoplasmic reticulum to nucleus signaling 1 | ERN1 | ENSG00000178607 | Apoptosis/Autophagy |
| glycogen synthase kinase 3 beta | GSK3B | ENSG00000082701 | Apoptosis/Autophagy |
| ABL proto-oncogene 1 / non-receptor tyrosine kinase | ABL1 | ENSG00000097007 | Apoptosis/Autophagy |
| pleckstrin homology and FYVE domain containing 1 | PLEKHF1 | ENSG00000166289 | Apoptosis/Autophagy |
| AKT serine/threonine kinase 1 | AKT1 | ENSG00000142208 | Apoptosis/Autophagy |
| F-box and WD repeat domain containing 7 | FBXW7 | ENSG00000109670 | Apoptosis/Autophagy |
| death associated protein kinase 3 | DAPK3 | ENSG00000167657 | Apoptosis/Autophagy |
| ring finger protein 41 | RNF41 | ENSG00000181852 | Apoptosis/Autophagy |
| MCL1 apoptosis regulator / BCL2 family member | MCL1 | ENSG00000143384 | Apoptosis/Autophagy |
| death associated protein | DAP | ENSG00000112977 | Apoptosis/Autophagy |
| inositol 1 /4 /5-trisphosphate receptor type 1 | ITPR1 | ENSG00000150995 | Apoptosis/Autophagy |
| DNA damage inducible transcript 3 | DDIT3 | ENSG00000175197 | Apoptosis/Autophagy |
| parkin RBR E3 ubiquitin protein ligase | PRKN | ENSG00000185345 | Apoptosis/Autophagy |
| TIR domain containing adaptor molecule 1 | TICAM1 | ENSG00000127666 | Apoptosis/Autophagy |
| ATM serine/threonine kinase | ATM | ENSG00000149311 | Apoptosis/Autophagy |
| serine/threonine kinase 11 | STK11 | ENSG00000118046 | Apoptosis/Autophagy |
| HtrA serine peptidase 2 | HTRA2 | ENSG00000115317 | Apoptosis/Autophagy |
| interferon gamma inducible protein 16 | IFI16 | ENSG00000163565 | Apoptosis/Autophagy |
| BCL2 like 11 | BCL2L11 | ENSG00000153094 | Apoptosis/Autophagy |
| Parkinsonism associated deglycase | PARK7 | ENSG00000116288 | Apoptosis/Autophagy |
| G protein subunit alpha i3 | GNAI3 | ENSG00000065135 | Apoptosis/Autophagy |
| beclin 1 | BECN1 | ENSG00000126581 | Apoptosis/Autophagy/Entotic.cell.death |
| interleukin 4 | IL4 | ENSG00000113520 | Apoptosis/Autophagy/Lysosome.dependent.cell.death |
| clusterin | CLU | ENSG00000120885 | Apoptosis/Autophagy/Lysosome.dependent.cell.death |
| leucine rich repeat kinase 2 | LRRK2 | ENSG00000188906 | Apoptosis/Autophagy/Lysosome.dependent.cell.death |
| BCL2 apoptosis regulator | BCL2 | ENSG00000171791 | Apoptosis/Autophagy/Necroptosis |
| dynamin 1 like | DNM1L | ENSG00000087470 | Apoptosis/Autophagy/Necroptosis |
| interferon gamma | IFNG | ENSG00000111537 | Apoptosis/Autophagy/Necroptosis |
| catenin alpha 1 | CTNNA1 | ENSG00000044115 | Apoptosis/Entotic.cell.death |
| colony stimulating factor 2 | CSF2 | ENSG00000164400 | Apoptosis/Ferroptosis |
| glutamate-cysteine ligase modifier subunit | GCLM | ENSG00000023909 | Apoptosis/Ferroptosis |
| ChaC glutathione specific gamma-glutamylcyclotransferase 1 | CHAC1 | ENSG00000128965 | Apoptosis/Ferroptosis |
| NADPH oxidase 1 | NOX1 | ENSG00000007952 | Apoptosis/Ferroptosis |
| CD44 molecule (Indian blood group) | CD44 | ENSG00000026508 | Apoptosis/Ferroptosis |
| NFE2 like bZIP transcription factor 2 | NFE2L2 | ENSG00000116044 | Apoptosis/Ferroptosis |
| heat shock protein family B (small) member 1 | HSPB1 | ENSG00000106211 | Apoptosis/Ferroptosis/Autophagy |
| transferrin | TF | ENSG00000091513 | Apoptosis/Ferroptosis/Autophagy/Cuproptosis/Lysosome.dependent.cell.death |
| heme oxygenase 1 | HMOX1 | ENSG00000100292 | Apoptosis/Ferroptosis/Autophagy/Lysosome.dependent.cell.death |
| voltage dependent anion channel 2 | VDAC2 | ENSG00000165637 | Apoptosis/Ferroptosis/Necroptosis |
| 3-phosphoinositide dependent protein kinase 1 | PDPK1 | ENSG00000140992 | Apoptosis/Lysosome.dependent.cell.death |
| X-box binding protein 1 | XBP1 | ENSG00000100219 | Apoptosis/Lysosome.dependent.cell.death |
| Bruton tyrosine kinase | BTK | ENSG00000010671 | Apoptosis/Lysosome.dependent.cell.death |
| cathepsin C | CTSC | ENSG00000109861 | Apoptosis/Lysosome.dependent.cell.death |
| KIT proto-oncogene / receptor tyrosine kinase | KIT | ENSG00000157404 | Apoptosis/Lysosome.dependent.cell.death |
| biogenesis of lysosomal organelles complex 1 subunit 2 | BLOC1S2 | ENSG00000196072 | Apoptosis/Lysosome.dependent.cell.death |
| sortilin 1 | SORT1 | ENSG00000134243 | Apoptosis/Lysosome.dependent.cell.death |
| receptor interacting serine/threonine kinase 3 | RIPK3 | ENSG00000285379 | Apoptosis/Necroptosis |
| BH3 interacting domain death agonist | BID | ENSG00000015475 | Apoptosis/Necroptosis |
| Janus kinase 2 | JAK2 | ENSG00000096968 | Apoptosis/Necroptosis |
| receptor interacting serine/threonine kinase 3 | RIPK3 | ENSG00000129465 | Apoptosis/Necroptosis |
| RANBP2-type and C3HC4-type zinc finger containing 1 | RBCK1 | ENSG00000125826 | Apoptosis/Necroptosis |
| TNF superfamily member 10 | TNFSF10 | ENSG00000121858 | Apoptosis/Necroptosis |
| receptor interacting serine/threonine kinase 1 | RIPK1 | ENSG00000137275 | Apoptosis/Necroptosis |
| CYLD lysine 63 deubiquitinase | CYLD | ENSG00000083799 | Apoptosis/Necroptosis |
| interleukin 33 | IL33 | ENSG00000137033 | Apoptosis/Necroptosis |
| TNFRSF1A associated via death domain | TRADD | ENSG00000102871 | Apoptosis/Necroptosis |
| TNF receptor superfamily member 10b | TNFRSF10B | ENSG00000120889 | Apoptosis/Necroptosis |
| TNF receptor superfamily member 10a | TNFRSF10A | ENSG00000104689 | Apoptosis/Necroptosis |
| TNF receptor associated factor 2 | TRAF2 | ENSG00000127191 | Apoptosis/Necroptosis |
| peptidylprolyl isomerase A | PPIA | ENSG00000196262 | Apoptosis/Necroptosis |
| toll like receptor 4 | TLR4 | ENSG00000136869 | Apoptosis/Necroptosis |
| toll like receptor 3 | TLR3 | ENSG00000164342 | Apoptosis/Necroptosis |
| Fas associated via death domain | FADD | ENSG00000168040 | Apoptosis/Necroptosis |
| poly(ADP-ribose) polymerase 1 | PARP1 | ENSG00000143799 | Apoptosis/Necroptosis |
| TNF alpha induced protein 3 | TNFAIP3 | ENSG00000118503 | Apoptosis/Necroptosis |
| TNF receptor superfamily member 1A | TNFRSF1A | ENSG00000067182 | Apoptosis/Necroptosis |
| CASP8 and FADD like apoptosis regulator | CFLAR | ENSG00000003402 | Apoptosis/Necroptosis |
| Fas associated factor 1 | FAF1 | ENSG00000185104 | Apoptosis/Necroptosis |
| apoptosis inducing factor mitochondria associated 1 | AIFM1 | ENSG00000156709 | Apoptosis/Necroptosis/Parthanatos/Oxeiptosis |
| macrophage migration inhibitory factor | MIF | ENSG00000276701 | Apoptosis/Parthanatos |
| macrophage migration inhibitory factor | MIF | ENSG00000240972 | Apoptosis/Parthanatos |
| gasdermin E | GSDME | ENSG00000105928 | Apoptosis/Pyroptosis |
| granzyme B | GZMB | ENSG00000100453 | Apoptosis/Pyroptosis |
| caspase 4 | CASP4 | ENSG00000196954 | Apoptosis/Pyroptosis |
| caspase 5 | CASP5 | ENSG00000137757 | Apoptosis/Pyroptosis |
| tumor protein p63 | TP63 | ENSG00000073282 | Apoptosis/Pyroptosis |
| interleukin 6 | IL6 | ENSG00000136244 | Apoptosis/Pyroptosis |
| BCL2 antagonist/killer 1 | BAK1 | ENSG00000030110 | Apoptosis/Pyroptosis |
| absent in melanoma 2 | AIM2 | ENSG00000163568 | Apoptosis/Pyroptosis |
| caspase 9 | CASP9 | ENSG00000132906 | Apoptosis/Pyroptosis |
| caspase 3 | CASP3 | ENSG00000164305 | Apoptosis/Pyroptosis/Autophagy |
| PYD and CARD domain containing | PYCARD | ENSG00000103490 | Apoptosis/Pyroptosis/Autophagy/Necroptosis |
| caspase 1 | CASP1 | ENSG00000137752 | Apoptosis/Pyroptosis/Autophagy/Necroptosis |
| tumor protein p53 | TP53 | ENSG00000141510 | Apoptosis/Pyroptosis/Ferroptosis/Autophagy |
| citrate synthase | CS | ENSG00000062485 | Apoptosis/Pyroptosis/Ferroptosis/Autophagy/Cuproptosis/Lysosome.dependent.cell.death |
| androgen receptor | AR | ENSG00000169083 | Apoptosis/Pyroptosis/Ferroptosis/Autophagy/Necroptosis/Parthanatos/Lysosome.dependent.cell.death |
| tumor necrosis factor | TNF | ENSG00000204490 | Apoptosis/Pyroptosis/Necroptosis |
| tumor necrosis factor | TNF | ENSG00000228321 | Apoptosis/Pyroptosis/Necroptosis |
| tumor necrosis factor | TNF | ENSG00000228849 | Apoptosis/Pyroptosis/Necroptosis |
| tumor necrosis factor | TNF | ENSG00000228978 | Apoptosis/Pyroptosis/Necroptosis |
| tumor necrosis factor | TNF | ENSG00000223952 | Apoptosis/Pyroptosis/Necroptosis |
| tumor necrosis factor | TNF | ENSG00000230108 | Apoptosis/Pyroptosis/Necroptosis |
| tumor necrosis factor | TNF | ENSG00000206439 | Apoptosis/Pyroptosis/Necroptosis |
| interleukin 1 beta | IL1B | ENSG00000125538 | Apoptosis/Pyroptosis/Necroptosis |
| tumor necrosis factor | TNF | ENSG00000232810 | Apoptosis/Pyroptosis/Necroptosis |
| BCL2 associated X / apoptosis regulator | BAX | ENSG00000087088 | Apoptosis/Pyroptosis/Necroptosis |
| caspase 8 | CASP8 | ENSG00000064012 | Apoptosis/Pyroptosis/Necroptosis |
| interleukin 1 alpha | IL1A | ENSG00000115008 | Apoptosis/Pyroptosis/Necroptosis |
| dehydrogenase/reductase X-linked | DHRSX | ENSG00000292338 | Autophagy |
| stimulator of interferon response cGAMP interactor 1 | STING1 | ENSG00000288243 | Autophagy |
| ubiquitin fold modifier 1 | UFM1 | ENSG00000120686 | Autophagy |
| forkhead box O1 | FOXO1 | ENSG00000150907 | Autophagy |
| suppressor of glucose / autophagy associated 1 | SOGA1 | ENSG00000149639 | Autophagy |
| ring finger protein 152 | RNF152 | ENSG00000176641 | Autophagy |
| tripartite motif containing 13 | TRIM13 | ENSG00000204977 | Autophagy |
| sirtuin 2 | SIRT2 | ENSG00000283100 | Autophagy |
| myotubularin related protein 9 | MTMR9 | ENSG00000285032 | Autophagy |
| sorting nexin 6 | SNX6 | ENSG00000129515 | Autophagy |
| dehydrogenase/reductase X-linked | DHRSX | ENSG00000169084 | Autophagy |
| translocator protein | TSPO | ENSG00000100300 | Autophagy |
| adrenoceptor alpha 1A | ADRA1A | ENSG00000120907 | Autophagy |
| DDRGK domain containing 1 | DDRGK1 | ENSG00000198171 | Autophagy |
| myotubularin related protein 3 | MTMR3 | ENSG00000100330 | Autophagy |
| myotubularin related protein 8 | MTMR8 | ENSG00000102043 | Autophagy |
| HECT and RLD domain containing E3 ubiquitin protein ligase family member 1 | HERC1 | ENSG00000103657 | Autophagy |
| sorting nexin 5 | SNX5 | ENSG00000089006 | Autophagy |
| ATPase H+ transporting V1 subunit E1 | ATP6V1E1 | ENSG00000131100 | Autophagy |
| autophagy related 2B | ATG2B | ENSG00000066739 | Autophagy |
| solute carrier family 38 member 9 | SLC38A9 | ENSG00000177058 | Autophagy |
| microtubule associated protein tau | MAPT | ENSG00000276155 | Autophagy |
| tripartite motif containing 27 | TRIM27 | ENSG00000204713 | Autophagy |
| microtubule associated protein tau | MAPT | ENSG00000277956 | Autophagy |
| forkhead box K1 | FOXK1 | ENSG00000164916 | Autophagy |
| metadherin | MTDH | ENSG00000147649 | Autophagy |
| WD repeat domain 81 | WDR81 | ENSG00000276021 | Autophagy |
| SMCR8-C9orf72 complex subunit | SMCR8 | ENSG00000283741 | Autophagy |
| RAB39B / member RAS oncogene family | RAB39B | ENSG00000155961 | Autophagy |
| DEPP autophagy regulator 1 | DEPP1 | ENSG00000165507 | Autophagy |
| CDGSH iron sulfur domain 2 | CISD2 | ENSG00000145354 | Autophagy |
| kelch like family member 22 | KLHL22 | ENSG00000099910 | Autophagy |
| lacritin | LACRT | ENSG00000135413 | Autophagy |
| triggering receptor expressed on myeloid cells 2 | TREM2 | ENSG00000095970 | Autophagy |
| ATPase H+ transporting V1 subunit C1 | ATP6V1C1 | ENSG00000155097 | Autophagy |
| cell division cycle 37 / HSP90 cochaperone | CDC37 | ENSG00000105401 | Autophagy |
| small VCP interacting protein | SVIP | ENSG00000198168 | Autophagy |
| sestrin 2 | SESN2 | ENSG00000285069 | Autophagy |
| synaptopodin 2 | SYNPO2 | ENSG00000172403 | Autophagy |
| forkhead box O3 | FOXO3 | ENSG00000118689 | Autophagy |
| NPR2 like / GATOR1 complex subunit | NPRL2 | ENSG00000114388 | Autophagy |
| ubiquilin 2 | UBQLN2 | ENSG00000188021 | Autophagy |
| late endosomal/lysosomal adaptor / MAPK and MTOR activator 3 | LAMTOR3 | ENSG00000109270 | Autophagy |
| transmembrane protein 150C | TMEM150C | ENSG00000249242 | Autophagy |
| lysine acetyltransferase 8 | KAT8 | ENSG00000103510 | Autophagy |
| Ras related GTP binding B | RRAGB | ENSG00000083750 | Autophagy |
| tripartite motif containing 27 | TRIM27 | ENSG00000237071 | Autophagy |
| ATPase H+ transporting V1 subunit G2 | ATP6V1G2 | ENSG00000226850 | Autophagy |
| ring finger protein 5 | RNF5 | ENSG00000228907 | Autophagy |
| ring finger protein 5 | RNF5 | ENSG00000228405 | Autophagy |
| tripartite motif containing 27 | TRIM27 | ENSG00000237462 | Autophagy |
| ATPase H+ transporting V1 subunit G2 | ATP6V1G2 | ENSG00000227587 | Autophagy |
| ORMDL sphingolipid biosynthesis regulator 3 | ORMDL3 | ENSG00000172057 | Autophagy |
| tripartite motif containing 27 | TRIM27 | ENSG00000234495 | Autophagy |
| ATPase H+ transporting V1 subunit G2 | ATP6V1G2 | ENSG00000234668 | Autophagy |
| ring finger protein 5 | RNF5 | ENSG00000223767 | Autophagy |
| mitogen-activated protein kinase 15 | MAPK15 | ENSG00000274205 | Autophagy |
| mitogen-activated protein kinase 3 | MAPK3 | ENSG00000102882 | Autophagy |
| Pim-2 proto-oncogene / serine/threonine kinase | PIM2 | ENSG00000292210 | Autophagy |
| vacuolar protein sorting 13 homolog D | VPS13D | ENSG00000048707 | Autophagy |
| tripartite motif containing 27 | TRIM27 | ENSG00000233948 | Autophagy |
| ATPase H+ transporting V1 subunit G2 | ATP6V1G2 | ENSG00000230900 | Autophagy |
| ring finger protein 5 | RNF5 | ENSG00000225452 | Autophagy |
| tripartite motif containing 27 | TRIM27 | ENSG00000215641 | Autophagy |
| tripartite motif containing 27 | TRIM27 | ENSG00000229006 | Autophagy |
| transmembrane protein 39A | TMEM39A | ENSG00000176142 | Autophagy |
| midline 2 | MID2 | ENSG00000080561 | Autophagy |
| fasciculation and elongation protein zeta 2 | FEZ2 | ENSG00000171055 | Autophagy |
| microtubule crosslinking factor 1 | MTCL1 | ENSG00000168502 | Autophagy |
| microRNA let-7b | MIRLET7B | ENSG00000284520 | Autophagy |
| ATPase H+ transporting V1 subunit G2 | ATP6V1G2 | ENSG00000234920 | Autophagy |
| ATPase H+ transporting V1 subunit G2 | ATP6V1G2 | ENSG00000206445 | Autophagy |
| sestrin 2 | SESN2 | ENSG00000130766 | Autophagy |
| ring finger protein 5 | RNF5 | ENSG00000183574 | Autophagy |
| EPM2A glucan phosphatase / laforin | EPM2A | ENSG00000112425 | Autophagy |
| ring finger protein 5 | RNF5 | ENSG00000227277 | Autophagy |
| fasciculation and elongation protein zeta 1 | FEZ1 | ENSG00000149557 | Autophagy |
| tripartite motif family like 1 | TRIML1 | ENSG00000184108 | Autophagy |
| ATPase H+ transporting V1 subunit D | ATP6V1D | ENSG00000100554 | Autophagy |
| Ras related GTP binding C | RRAGC | ENSG00000116954 | Autophagy |
| forkhead box K2 | FOXK2 | ENSG00000141568 | Autophagy |
| sec1 family domain containing 1 | SCFD1 | ENSG00000092108 | Autophagy |
| mitogen-activated protein kinase 15 | MAPK15 | ENSG00000181085 | Autophagy |
| VPS26 retromer complex component A | VPS26A | ENSG00000122958 | Autophagy |
| ATPase H+ transporting V1 subunit G1 | ATP6V1G1 | ENSG00000136888 | Autophagy |
| nuclear receptor binding protein 2 | NRBP2 | ENSG00000185189 | Autophagy |
| tumor protein p53 inducible nuclear protein 1 | TP53INP1 | ENSG00000164938 | Autophagy |
| Ras related GTP binding A | RRAGA | ENSG00000155876 | Autophagy |
| SPT5 homolog / DSIF elongation factor subunit | SUPT5H | ENSG00000196235 | Autophagy |
| tripartite motif containing 6 | TRIM6 | ENSG00000121236 | Autophagy |
| DNA polymerase delta interacting protein 2 | POLDIP2 | ENSG00000004142 | Autophagy |
| autophagy related 14 | ATG14 | ENSG00000126775 | Autophagy |
| calcium/calmodulin dependent protein kinase kinase 2 | CAMKK2 | ENSG00000110931 | Autophagy |
| tripartite motif containing 38 | TRIM38 | ENSG00000112343 | Autophagy |
| Rho associated coiled-coil containing protein kinase 1 | ROCK1 | ENSG00000067900 | Autophagy |
| Ras homolog / mTORC1 binding | RHEB | ENSG00000106615 | Autophagy |
| leucine rich repeat and sterile alpha motif containing 1 | LRSAM1 | ENSG00000148356 | Autophagy |
| DNA damage regulated autophagy modulator 1 | DRAM1 | ENSG00000136048 | Autophagy |
| lysine demethylase 4A | KDM4A | ENSG00000066135 | Autophagy |
| TGF-beta activated kinase 1 (MAP3K7) binding protein 3 | TAB3 | ENSG00000157625 | Autophagy |
| eukaryotic translation elongation factor 1 alpha 1 | EEF1A1 | ENSG00000156508 | Autophagy |
| ubiquitin specific peptidase 30 | USP30 | ENSG00000135093 | Autophagy |
| transmembrane protein 150B | TMEM150B | ENSG00000180061 | Autophagy |
| myotubularin 1 | MTM1 | ENSG00000171100 | Autophagy |
| G protein signaling modulator 1 | GPSM1 | ENSG00000160360 | Autophagy |
| ubiquitin specific peptidase 10 | USP10 | ENSG00000103194 | Autophagy |
| endosome-lysosome associated apoptosis and autophagy regulator 1 | ELAPOR1 | ENSG00000116299 | Autophagy |
| cyclin dependent kinase 5 | CDK5 | ENSG00000164885 | Autophagy |
| ATPase H+ transporting V1 subunit G2 | ATP6V1G2 | ENSG00000213760 | Autophagy |
| eukaryotic translation elongation factor 1 alpha 2 | EEF1A2 | ENSG00000101210 | Autophagy |
| RAS like proto-oncogene B | RALB | ENSG00000144118 | Autophagy |
| DEP domain containing 5 / GATOR1 subcomplex subunit | DEPDC5 | ENSG00000100150 | Autophagy |
| phosphatidylinositol-4 /5-bisphosphate 3-kinase catalytic subunit beta | PIK3CB | ENSG00000051382 | Autophagy |
| phosphoinositide-3-kinase regulatory subunit 2 | PIK3R2 | ENSG00000105647 | Autophagy |
| phosphatidylinositol-5-phosphate 4-kinase type 2 gamma | PIP4K2C | ENSG00000166908 | Autophagy |
| tumor protein p53 inducible nuclear protein 2 | TP53INP2 | ENSG00000078804 | Autophagy |
| myotubularin related protein 9 | MTMR9 | ENSG00000104643 | Autophagy |
| F-box protein 7 | FBXO7 | ENSG00000100225 | Autophagy |
| SEC22 homolog B / vesicle trafficking protein | SEC22B | ENSG00000265808 | Autophagy |
| protein kinase D1 | PRKD1 | ENSG00000184304 | Autophagy |
| platelet activating factor acetylhydrolase 1b catalytic subunit 2 | PAFAH1B2 | ENSG00000168092 | Autophagy |
| sterol regulatory element binding transcription factor 2 | SREBF2 | ENSG00000198911 | Autophagy |
| ring finger protein 5 | RNF5 | ENSG00000204308 | Autophagy |
| two pore segment channel 2 | TPCN2 | ENSG00000162341 | Autophagy |
| SH3 domain binding protein 4 | SH3BP4 | ENSG00000130147 | Autophagy |
| tripartite motif containing 34 | TRIM34 | ENSG00000258659 | Autophagy |
| tripartite motif containing 65 | TRIM65 | ENSG00000141569 | Autophagy |
| decorin | DCN | ENSG00000011465 | Autophagy |
| adrenoceptor beta 2 | ADRB2 | ENSG00000169252 | Autophagy |
| WD repeat domain 81 | WDR81 | ENSG00000167716 | Autophagy |
| calcium binding and coiled-coil domain 2 | CALCOCO2 | ENSG00000136436 | Autophagy |
| small nuclear ribonucleoprotein U1 subunit 70 | SNRNP70 | ENSG00000104852 | Autophagy |
| eukaryotic translation initiation factor 4 gamma 1 | EIF4G1 | ENSG00000114867 | Autophagy |
| serine palmitoyltransferase long chain base subunit 1 | SPTLC1 | ENSG00000090054 | Autophagy |
| alkaline ceramidase 2 | ACER2 | ENSG00000177076 | Autophagy |
| kinase insert domain receptor | KDR | ENSG00000128052 | Autophagy |
| exocyst complex component 7 | EXOC7 | ENSG00000182473 | Autophagy |
| TP53 induced glycolysis regulatory phosphatase | TIGAR | ENSG00000078237 | Autophagy |
| transmembrane protein 59 | TMEM59 | ENSG00000116209 | Autophagy |
| short coiled-coil protein | SCOC | ENSG00000153130 | Autophagy |
| TBC1 domain family member 25 | TBC1D25 | ENSG00000068354 | Autophagy |
| ATPase H+ transporting V1 subunit E2 | ATP6V1E2 | ENSG00000250565 | Autophagy |
| heat shock protein family B (small) member 8 | HSPB8 | ENSG00000152137 | Autophagy |
| starch binding domain 1 | STBD1 | ENSG00000118804 | Autophagy |
| polo like kinase 2 | PLK2 | ENSG00000145632 | Autophagy |
| exocyst complex component 8 | EXOC8 | ENSG00000116903 | Autophagy |
| WASH complex subunit 1 | WASHC1 | ENSG00000181404 | Autophagy |
| AUP1 lipid droplet regulating VLDL assembly factor | AUP1 | ENSG00000115307 | Autophagy |
| microRNA 199a-1 | MIR199A1 | ENSG00000207752 | Autophagy |
| optineurin | OPTN | ENSG00000123240 | Autophagy |
| receptor interacting serine/threonine kinase 2 | RIPK2 | ENSG00000104312 | Autophagy |
| lysine acetyltransferase 5 | KAT5 | ENSG00000172977 | Autophagy |
| tripartite motif family like 2 | TRIML2 | ENSG00000179046 | Autophagy |
| sorting nexin 32 | SNX32 | ENSG00000172803 | Autophagy |
| reticulophagy regulator family member 3 | RETREG3 | ENSG00000141699 | Autophagy |
| reticulophagy regulator 1 | RETREG1 | ENSG00000154153 | Autophagy |
| UFM1 specific ligase 1 | UFL1 | ENSG00000014123 | Autophagy |
| C9orf72-SMCR8 complex subunit | C9orf72 | ENSG00000147894 | Autophagy |
| kinesin family member 25 | KIF25 | ENSG00000125337 | Autophagy |
| zinc finger CCCH-type containing 12A | ZC3H12A | ENSG00000163874 | Autophagy |
| HECT / UBA and WWE domain containing E3 ubiquitin protein ligase 1 | HUWE1 | ENSG00000086758 | Autophagy |
| two pore segment channel 1 | TPCN1 | ENSG00000186815 | Autophagy |
| serine palmitoyltransferase long chain base subunit 2 | SPTLC2 | ENSG00000100596 | Autophagy |
| ERCC excision repair 4 / endonuclease catalytic subunit | ERCC4 | ENSG00000175595 | Autophagy |
| 5-hydroxytryptamine receptor 2B | HTR2B | ENSG00000135914 | Autophagy |
| golgin A2 | GOLGA2 | ENSG00000167110 | Autophagy |
| heat shock protein 90 alpha family class A member 1 | HSP90AA1 | ENSG00000080824 | Autophagy |
| tripartite motif containing 14 | TRIM14 | ENSG00000106785 | Autophagy |
| ubiquilin 4 | UBQLN4 | ENSG00000160803 | Autophagy |
| Ras interacting protein 1 | RASIP1 | ENSG00000105538 | Autophagy |
| ATPase H+ transporting V0 subunit e1 | ATP6V0E1 | ENSG00000113732 | Autophagy |
| leptin receptor | LEPR | ENSG00000116678 | Autophagy |
| synuclein alpha | SNCA | ENSG00000145335 | Autophagy |
| glyceraldehyde-3-phosphate dehydrogenase | GAPDH | ENSG00000111640 | Autophagy |
| La ribonucleoprotein 1 / translational regulator | LARP1 | ENSG00000155506 | Autophagy |
| ATPase H+ transporting V0 subunit e2 | ATP6V0E2 | ENSG00000171130 | Autophagy |
| calpain small subunit 1 | CAPNS1 | ENSG00000126247 | Autophagy |
| protein kinase AMP-activated non-catalytic subunit beta 1 | PRKAB1 | ENSG00000111725 | Autophagy |
| protein kinase AMP-activated catalytic subunit alpha 1 | PRKAA1 | ENSG00000132356 | Autophagy |
| prohibitin 2 | PHB2 | ENSG00000215021 | Autophagy |
| SMG1 nonsense mediated mRNA decay associated PI3K related kinase | SMG1 | ENSG00000157106 | Autophagy |
| Pim-2 proto-oncogene / serine/threonine kinase | PIM2 | ENSG00000102096 | Autophagy |
| tripartite motif containing 5 | TRIM5 | ENSG00000132256 | Autophagy |
| ubiquitin-fold modifier conjugating enzyme 1 | UFC1 | ENSG00000143222 | Autophagy |
| ubiquitin C-terminal hydrolase L1 | UCHL1 | ENSG00000154277 | Autophagy |
| WW domain containing adaptor with coiled-coil | WAC | ENSG00000095787 | Autophagy |
| vacuolar protein sorting 13 homolog C | VPS13C | ENSG00000129003 | Autophagy |
| mitofusin 2 | MFN2 | ENSG00000116688 | Autophagy |
| tousled like kinase 2 | TLK2 | ENSG00000146872 | Autophagy |
| zinc finger with KRAB and SCAN domains 3 | ZKSCAN3 | ENSG00000189298 | Autophagy |
| zinc metallopeptidase STE24 | ZMPSTE24 | ENSG00000084073 | Autophagy |
| TANK binding kinase 1 | TBK1 | ENSG00000183735 | Autophagy |
| ubiquitin specific peptidase 13 | USP13 | ENSG00000058056 | Autophagy |
| polo like kinase 3 | PLK3 | ENSG00000173846 | Autophagy |
| eukaryotic translation initiation factor 2 alpha kinase 4 | EIF2AK4 | ENSG00000128829 | Autophagy |
| WD repeat domain 41 | WDR41 | ENSG00000164253 | Autophagy |
| unc-51 like autophagy activating kinase 1 | ULK1 | ENSG00000177169 | Autophagy |
| translocase of outer mitochondrial membrane 7 | TOMM7 | ENSG00000196683 | Autophagy |
| myotubularin related protein 4 | MTMR4 | ENSG00000108389 | Autophagy |
| stimulator of interferon response cGAMP interactor 1 | STING1 | ENSG00000184584 | Autophagy |
| ATPase H+ transporting V1 subunit B2 | ATP6V1B2 | ENSG00000147416 | Autophagy |
| leucine zipper tumor suppressor 1 | LZTS1 | ENSG00000061337 | Autophagy |
| PHD finger protein 23 | PHF23 | ENSG00000040633 | Autophagy |
| glial fibrillary acidic protein | GFAP | ENSG00000131095 | Autophagy |
| MEFV innate immunity regulator / pyrin | MEFV | ENSG00000103313 | Autophagy |
| TSC complex subunit 1 | TSC1 | ENSG00000165699 | Autophagy |
| tripartite motif containing 8 | TRIM8 | ENSG00000171206 | Autophagy |
| STIP1 homology and U-box containing protein 1 | STUB1 | ENSG00000103266 | Autophagy |
| WD repeat domain 24 | WDR24 | ENSG00000127580 | Autophagy |
| phosphatidylinositol-4 /5-bisphosphate 3-kinase catalytic subunit alpha | PIK3CA | ENSG00000121879 | Autophagy |
| TGF-beta activated kinase 1 (MAP3K7) binding protein 2 | TAB2 | ENSG00000055208 | Autophagy |
| late endosomal/lysosomal adaptor / MAPK and MTOR activator 4 | LAMTOR4 | ENSG00000188186 | Autophagy |
| SOGA family member 3 | SOGA3 | ENSG00000214338 | Autophagy |
| autophagy and beclin 1 regulator 1 | AMBRA1 | ENSG00000110497 | Autophagy |
| ubiquitin like modifier activating enzyme 5 | UBA5 | ENSG00000081307 | Autophagy |
| galectin 8 | LGALS8 | ENSG00000116977 | Autophagy |
| phosphatidylinositol-4-phosphate 3-kinase catalytic subunit type 2 alpha | PIK3C2A | ENSG00000011405 | Autophagy |
| C-type lectin domain containing 16A | CLEC16A | ENSG00000038532 | Autophagy |
| MTOR associated protein / LST8 homolog | MLST8 | ENSG00000167965 | Autophagy |
| WD repeat and FYVE domain containing 3 | WDFY3 | ENSG00000163625 | Autophagy |
| protein kinase AMP-activated non-catalytic subunit gamma 1 | PRKAG1 | ENSG00000181929 | Autophagy |
| autophagy related 101 | ATG101 | ENSG00000123395 | Autophagy |
| late endosomal/lysosomal adaptor / MAPK and MTOR activator 5 | LAMTOR5 | ENSG00000134248 | Autophagy |
| exocyst complex component 4 | EXOC4 | ENSG00000131558 | Autophagy |
| TBC1 domain family member 14 | TBC1D14 | ENSG00000132405 | Autophagy |
| mitogen-activated protein kinase kinase kinase 7 | MAP3K7 | ENSG00000135341 | Autophagy |
| ubiquitin specific peptidase 36 | USP36 | ENSG00000055483 | Autophagy |
| exocyst complex component 1 | EXOC1 | ENSG00000090989 | Autophagy |
| DNA damage regulated autophagy modulator 2 | DRAM2 | ENSG00000156171 | Autophagy |
| kelch like family member 3 | KLHL3 | ENSG00000146021 | Autophagy |
| VPS35 retromer complex component | VPS35 | ENSG00000069329 | Autophagy |
| Ras related GTP binding D | RRAGD | ENSG00000025039 | Autophagy |
| VPS26 retromer complex component B | VPS26B | ENSG00000151502 | Autophagy |
| immunity related GTPase M | IRGM | ENSG00000237693 | Autophagy |
| sestrin 1 | SESN1 | ENSG00000080546 | Autophagy |
| TSC complex subunit 2 | TSC2 | ENSG00000103197 | Autophagy |
| WD repeat domain 6 | WDR6 | ENSG00000178252 | Autophagy |
| activating transcription factor 6 | ATF6 | ENSG00000118217 | Autophagy |
| WD repeat domain / phosphoinositide interacting 2 | WIPI2 | ENSG00000157954 | Autophagy |
| regulatory associated protein of MTOR complex 1 | RPTOR | ENSG00000141564 | Autophagy |
| testis expressed 264 / ER-phagy receptor | TEX264 | ENSG00000164081 | Autophagy |
| transmembrane protein 39B | TMEM39B | ENSG00000121775 | Autophagy |
| FYVE and coiled-coil domain autophagy adaptor 1 | FYCO1 | ENSG00000163820 | Autophagy |
| interleukin 10 receptor subunit alpha | IL10RA | ENSG00000110324 | Autophagy |
| MET proto-oncogene / receptor tyrosine kinase | MET | ENSG00000105976 | Autophagy |
| RAB8A / member RAS oncogene family | RAB8A | ENSG00000167461 | Autophagy |
| leptin | LEP | ENSG00000174697 | Autophagy |
| protein kinase AMP-activated non-catalytic subunit gamma 2 | PRKAG2 | ENSG00000106617 | Autophagy |
| sestrin 3 | SESN3 | ENSG00000149212 | Autophagy |
| sirtuin 2 | SIRT2 | ENSG00000068903 | Autophagy |
| tripartite motif containing 22 | TRIM22 | ENSG00000132274 | Autophagy |
| RAB3 GTPase activating protein catalytic subunit 1 | RAB3GAP1 | ENSG00000115839 | Autophagy |
| SMCR8-C9orf72 complex subunit | SMCR8 | ENSG00000176994 | Autophagy |
| eukaryotic translation initiation factor 4 gamma 2 | EIF4G2 | ENSG00000110321 | Autophagy |
| microtubule associated protein tau | MAPT | ENSG00000186868 | Autophagy |
| transmembrane protein 150A | TMEM150A | ENSG00000168890 | Autophagy |
| cyclin dependent kinase 5 regulatory subunit 1 | CDK5R1 | ENSG00000176749 | Autophagy |
| ATPase H+ transporting V1 subunit B1 | ATP6V1B1 | ENSG00000116039 | Autophagy |
| ATPase H+ transporting V1 subunit C2 | ATP6V1C2 | ENSG00000143882 | Autophagy |
| autophagy related 13 | ATG13 | ENSG00000175224 | Autophagy |
| frizzled class receptor 5 | FZD5 | ENSG00000163251 | Autophagy |
| tripartite motif containing 68 | TRIM68 | ENSG00000167333 | Autophagy |
| RUN and FYVE domain containing 4 | RUFY4 | ENSG00000188282 | Autophagy |
| tripartite motif containing 21 | TRIM21 | ENSG00000132109 | Autophagy |
| G protein-coupled receptor 137 | GPR137 | ENSG00000173264 | Autophagy |
| protein kinase AMP-activated catalytic subunit alpha 2 | PRKAA2 | ENSG00000162409 | Autophagy |
| autophagy related 2A | ATG2A | ENSG00000110046 | Autophagy |
| sterol regulatory element binding transcription factor 1 | SREBF1 | ENSG00000072310 | Autophagy |
| ATPase H+ transporting V1 subunit A | ATP6V1A | ENSG00000114573 | Autophagy |
| oxysterol binding protein like 7 | OSBPL7 | ENSG00000006025 | Autophagy |
| RAB3 GTPase activating non-catalytic protein subunit 2 | RAB3GAP2 | ENSG00000118873 | Autophagy |
| F-box and leucine rich repeat protein 2 | FBXL2 | ENSG00000153558 | Autophagy |
| G protein-coupled receptor 137B | GPR137B | ENSG00000077585 | Autophagy |
| protein kinase AMP-activated non-catalytic subunit beta 2 | PRKAB2 | ENSG00000131791 | Autophagy |
| protein kinase AMP-activated non-catalytic subunit gamma 3 | PRKAG3 | ENSG00000115592 | Autophagy |
| interleukin 10 | IL10 | ENSG00000136634 | Autophagy |
| late endosomal/lysosomal adaptor / MAPK and MTOR activator 2 | LAMTOR2 | ENSG00000116586 | Autophagy |
| protein tyrosine phosphatase non-receptor type 22 | PTPN22 | ENSG00000134242 | Autophagy |
| HCLS1 associated protein X-1 | HAX1 | ENSG00000143575 | Autophagy |
| ceramide-1-phosphate transfer protein | CPTP | ENSG00000224051 | Autophagy |
| ABL proto-oncogene 2 / non-receptor tyrosine kinase | ABL2 | ENSG00000143322 | Autophagy |
| quiescin sulfhydryl oxidase 1 | QSOX1 | ENSG00000116260 | Autophagy |
| SH3 domain containing GRB2 like / endophilin B1 | SH3GLB1 | ENSG00000097033 | Autophagy |
| ubiquitin specific peptidase 33 | USP33 | ENSG00000077254 | Autophagy |
| inhibitor of nuclear factor kappa B kinase regulatory subunit gamma | IKBKG | ENSG00000269335 | Autophagy/Alkaliptosis |
| UV radiation resistance associated | UVRAG | ENSG00000198382 | Autophagy/Entotic.cell.death |
| rubicon autophagy regulator | RUBCN | ENSG00000145016 | Autophagy/Entotic.cell.death/Lysosome.dependent.cell.death |
| lysosomal associated membrane protein 1 | LAMP1 | ENSG00000185896 | Autophagy/Lysosome.dependent.cell.death |
| phosphatidylinositol-5-phosphate 4-kinase type 2 alpha | PIP4K2A | ENSG00000150867 | Autophagy/Lysosome.dependent.cell.death |
| phosphatidylinositol-5-phosphate 4-kinase type 2 beta | PIP4K2B | ENSG00000277292 | Autophagy/Lysosome.dependent.cell.death |
| ATPase H+ transporting V0 subunit d2 | ATP6V0D2 | ENSG00000147614 | Autophagy/Lysosome.dependent.cell.death |
| phosphatidylinositol 3-kinase catalytic subunit type 3 | PIK3C3 | ENSG00000078142 | Autophagy/Lysosome.dependent.cell.death |
| ATPase H+ transporting V1 subunit H | ATP6V1H | ENSG00000047249 | Autophagy/Lysosome.dependent.cell.death |
| metallothionein 3 | MT3 | ENSG00000087250 | Autophagy/Lysosome.dependent.cell.death |
| heat shock protein family A (Hsp70) member 8 | HSPA8 | ENSG00000109971 | Autophagy/Lysosome.dependent.cell.death |
| aspartylglucosaminidase | AGA | ENSG00000038002 | Autophagy/Lysosome.dependent.cell.death |
| NPC intracellular cholesterol transporter 1 | NPC1 | ENSG00000141458 | Autophagy/Lysosome.dependent.cell.death |
| ATPase H+ transporting V0 subunit c | ATP6V0C | ENSG00000185883 | Autophagy/Lysosome.dependent.cell.death |
| folliculin | FLCN | ENSG00000154803 | Autophagy/Lysosome.dependent.cell.death |
| transcription factor EB | TFEB | ENSG00000112561 | Autophagy/Lysosome.dependent.cell.death |
| late endosomal/lysosomal adaptor / MAPK and MTOR activator 1 | LAMTOR1 | ENSG00000149357 | Autophagy/Lysosome.dependent.cell.death |
| cathepsin A | CTSA | ENSG00000064601 | Autophagy/Lysosome.dependent.cell.death |
| prosaposin | PSAP | ENSG00000197746 | Autophagy/Lysosome.dependent.cell.death |
| NEDD4 E3 ubiquitin protein ligase | NEDD4 | ENSG00000069869 | Autophagy/Lysosome.dependent.cell.death |
| RAB7A / member RAS oncogene family | RAB7A | ENSG00000075785 | Autophagy/Lysosome.dependent.cell.death |
| CLN3 lysosomal/endosomal transmembrane protein / battenin | CLN3 | ENSG00000188603 | Autophagy/Lysosome.dependent.cell.death |
| histone deacetylase 6 | HDAC6 | ENSG00000094631 | Autophagy/Lysosome.dependent.cell.death |
| ATPase H+ transporting V0 subunit a2 | ATP6V0A2 | ENSG00000185344 | Autophagy/Lysosome.dependent.cell.death |
| lysosomal associated membrane protein 2 | LAMP2 | ENSG00000005893 | Autophagy/Lysosome.dependent.cell.death |
| ATPase H+ transporting V0 subunit d1 | ATP6V0D1 | ENSG00000159720 | Autophagy/Lysosome.dependent.cell.death |
| ATPase cation transporting 13A2 | ATP13A2 | ENSG00000159363 | Autophagy/Lysosome.dependent.cell.death |
| major facilitator superfamily domain containing 8 | MFSD8 | ENSG00000164073 | Autophagy/Lysosome.dependent.cell.death |
| ATPase H+ transporting V0 subunit b | ATP6V0B | ENSG00000117410 | Autophagy/Lysosome.dependent.cell.death |
| phosphatidylinositol-5-phosphate 4-kinase type 2 beta | PIP4K2B | ENSG00000276293 | Autophagy/Lysosome.dependent.cell.death |
| ATPase H+ transporting V0 subunit a1 | ATP6V0A1 | ENSG00000033627 | Autophagy/Lysosome.dependent.cell.death |
| lysosomal associated membrane protein 3 | LAMP3 | ENSG00000078081 | Autophagy/Lysosome.dependent.cell.death |
| galactosidase beta 1 | GLB1 | ENSG00000170266 | Autophagy/Lysosome.dependent.cell.death |
| mechanistic target of rapamycin kinase | MTOR | ENSG00000198793 | Autophagy/Lysosome.dependent.cell.death |
| signal transducer and activator of transcription 3 | STAT3 | ENSG00000168610 | Autophagy/Necroptosis |
| voltage dependent anion channel 1 | VDAC1 | ENSG00000213585 | Autophagy/Necroptosis |
| calpain 1 | CAPN1 | ENSG00000014216 | Autophagy/Necroptosis |
| sequestosome 1 | SQSTM1 | ENSG00000284099 | Autophagy/Necroptosis/Lysosome.dependent.cell.death |
| sequestosome 1 | SQSTM1 | ENSG00000161011 | Autophagy/Necroptosis/Lysosome.dependent.cell.death |
| glycine cleavage system protein H | GCSH | ENSG00000291958 | Cuproptosis |
| ATPase copper transporting beta | ATP7B | ENSG00000123191 | Cuproptosis |
| ferredoxin 1 | FDX1 | ENSG00000137714 | Cuproptosis |
| glycine cleavage system protein H | GCSH | ENSG00000140905 | Cuproptosis |
| lipoic acid synthetase | LIAS | ENSG00000121897 | Cuproptosis |
| ATPase copper transporting alpha | ATP7A | ENSG00000165240 | Cuproptosis |
| solute carrier family 31 member 1 | SLC31A1 | ENSG00000136868 | Cuproptosis |
| pyruvate dehydrogenase E1 subunit alpha 1 | PDHA1 | ENSG00000131828 | Cuproptosis |
| cyclin dependent kinase inhibitor 2A | CDKN2A | ENSG00000147889 | Cuproptosis |
| dihydrolipoamide S-acetyltransferase | DLAT | ENSG00000150768 | Cuproptosis |
| lipoyltransferase 1 | LIPT1 | ENSG00000144182 | Cuproptosis |
| pyruvate dehydrogenase E1 subunit beta | PDHB | ENSG00000168291 | Cuproptosis |
| dihydrolipoamide dehydrogenase | DLD | ENSG00000091140 | Cuproptosis |
| metal regulatory transcription factor 1 | MTF1 | ENSG00000188786 | Cuproptosis |
| linker for activation of T cells | LAT | ENSG00000213658 | Cuproptosis/Lysosome.dependent.cell.death |
| cadherin 1 | CDH1 | ENSG00000039068 | Entotic.cell.death |
| ras homolog family member A | RHOA | ENSG00000067560 | Entotic.cell.death |
| myosin heavy chain 14 | MYH14 | ENSG00000105357 | Entotic.cell.death |
| cell division cycle 42 | CDC42 | ENSG00000070831 | Entotic.cell.death |
| BTB domain and CNC homolog 1 | BACH1 | ENSG00000156273 | Ferroptosis |
| cystathionine beta-synthase | CBS | ENSG00000160200 | Ferroptosis |
| farnesyl-diphosphate farnesyltransferase 1 | FDFT1 | ENSG00000284967 | Ferroptosis |
| arachidonate 5-lipoxygenase | ALOX5 | ENSG00000275565 | Ferroptosis |
| aldo-keto reductase family 1 member C3 | AKR1C3 | ENSG00000196139 | Ferroptosis |
| ATP binding cassette subfamily C member 1 | ABCC1 | ENSG00000278183 | Ferroptosis |
| prion protein | PRNP | ENSG00000171867 | Ferroptosis |
| NFS1 cysteine desulfurase | NFS1 | ENSG00000244005 | Ferroptosis |
| iron responsive element binding protein 2 | IREB2 | ENSG00000136381 | Ferroptosis |
| acetyl-CoA carboxylase alpha | ACACA | ENSG00000275176 | Ferroptosis |
| prominin 2 | PROM2 | ENSG00000292084 | Ferroptosis |
| acetyl-CoA carboxylase alpha | ACACA | ENSG00000278540 | Ferroptosis |
| phosphorylase kinase catalytic subunit gamma 2 | PHKG2 | ENSG00000156873 | Ferroptosis |
| solute carrier family 7 member 11 | SLC7A11 | ENSG00000151012 | Ferroptosis |
| glutathione synthetase | GSS | ENSG00000100983 | Ferroptosis |
| ferritin mitochondrial | FTMT | ENSG00000181867 | Ferroptosis |
| apoptosis inducing factor mitochondria associated 2 | AIFM2 | ENSG00000042286 | Ferroptosis |
| acyl-CoA synthetase long chain family member 4 | ACSL4 | ENSG00000068366 | Ferroptosis |
| ER membrane protein complex subunit 2 | EMC2 | ENSG00000104412 | Ferroptosis |
| spermidine/spermine N1-acetyltransferase family member 2 | SAT2 | ENSG00000141504 | Ferroptosis |
| spermidine/spermine N1-acetyltransferase 1 | SAT1 | ENSG00000130066 | Ferroptosis |
| aconitase 1 | ACO1 | ENSG00000122729 | Ferroptosis |
| acyl-CoA synthetase long chain family member 6 | ACSL6 | ENSG00000164398 | Ferroptosis |
| crystallin alpha B | CRYAB | ENSG00000109846 | Ferroptosis |
| solute carrier family 39 member 14 | SLC39A14 | ENSG00000104635 | Ferroptosis |
| phosphatidylethanolamine binding protein 1 | PEBP1 | ENSG00000089220 | Ferroptosis |
| zinc finger E-box binding homeobox 1 | ZEB1 | ENSG00000148516 | Ferroptosis |
| acyl-CoA synthetase long chain family member 5 | ACSL5 | ENSG00000197142 | Ferroptosis |
| ribosomal protein L8 | RPL8 | ENSG00000161016 | Ferroptosis |
| STEAP3 metalloreductase | STEAP3 | ENSG00000115107 | Ferroptosis |
| arachidonate 5-lipoxygenase | ALOX5 | ENSG00000012779 | Ferroptosis |
| lysophosphatidylcholine acyltransferase 3 | LPCAT3 | ENSG00000111684 | Ferroptosis |
| ATP binding cassette subfamily C member 1 | ABCC1 | ENSG00000103222 | Ferroptosis |
| aldo-keto reductase family 1 member C2 | AKR1C2 | ENSG00000151632 | Ferroptosis |
| glucose-6-phosphate dehydrogenase | G6PD | ENSG00000160211 | Ferroptosis |
| 3-hydroxy-3-methylglutaryl-CoA reductase | HMGCR | ENSG00000113161 | Ferroptosis |
| aldo-keto reductase family 1 member C1 | AKR1C1 | ENSG00000187134 | Ferroptosis |
| solute carrier family 39 member 8 | SLC39A8 | ENSG00000138821 | Ferroptosis |
| glutamic-oxaloacetic transaminase 1 | GOT1 | ENSG00000120053 | Ferroptosis |
| metallothionein 1G | MT1G | ENSG00000125144 | Ferroptosis |
| solute carrier family 40 member 1 | SLC40A1 | ENSG00000138449 | Ferroptosis |
| phosphogluconate dehydrogenase | PGD | ENSG00000142657 | Ferroptosis |
| CDGSH iron sulfur domain 1 | CISD1 | ENSG00000122873 | Ferroptosis |
| squalene epoxidase | SQLE | ENSG00000104549 | Ferroptosis |
| fatty acid desaturase 2 | FADS2 | ENSG00000134824 | Ferroptosis |
| dipeptidyl peptidase 4 | DPP4 | ENSG00000197635 | Ferroptosis |
| heat shock factor binding protein 1 | HSBP1 | ENSG00000230989 | Ferroptosis |
| glutamate-cysteine ligase catalytic subunit | GCLC | ENSG00000001084 | Ferroptosis |
| arachidonate 12-lipoxygenase / 12S type | ALOX12 | ENSG00000108839 | Ferroptosis |
| farnesyl-diphosphate farnesyltransferase 1 | FDFT1 | ENSG00000079459 | Ferroptosis |
| ATP synthase membrane subunit c locus 3 | ATP5MC3 | ENSG00000154518 | Ferroptosis |
| NAD(P)H quinone dehydrogenase 1 | NQO1 | ENSG00000181019 | Ferroptosis |
| FA complementation group D2 | FANCD2 | ENSG00000144554 | Ferroptosis |
| acyl-CoA synthetase long chain family member 3 | ACSL3 | ENSG00000123983 | Ferroptosis |
| acyl-CoA synthetase long chain family member 1 | ACSL1 | ENSG00000151726 | Ferroptosis |
| poly(rC) binding protein 2 | PCBP2 | ENSG00000197111 | Ferroptosis |
| poly(rC) binding protein 1 | PCBP1 | ENSG00000169564 | Ferroptosis |
| prostaglandin-endoperoxide synthase 2 | PTGS2 | ENSG00000073756 | Ferroptosis |
| acyl-CoA synthetase family member 2 | ACSF2 | ENSG00000167107 | Ferroptosis |
| solute carrier family 3 member 2 | SLC3A2 | ENSG00000168003 | Ferroptosis |
| solute carrier family 1 member 5 | SLC1A5 | ENSG00000105281 | Ferroptosis |
| transferrin receptor | TFRC | ENSG00000072274 | Ferroptosis |
| glutaminase 2 | GLS2 | ENSG00000135423 | Ferroptosis |
| prominin 2 | PROM2 | ENSG00000155066 | Ferroptosis |
| OTU deubiquitinase / ubiquitin aldehyde binding 1 | OTUB1 | ENSG00000167770 | Ferroptosis |
| microtubule associated protein 1 light chain 3 beta | MAP1LC3B | ENSG00000140941 | Ferroptosis/Autophagy |
| microtubule associated protein 1 light chain 3 gamma | MAP1LC3C | ENSG00000197769 | Ferroptosis/Autophagy |
| autophagy related 5 | ATG5 | ENSG00000057663 | Ferroptosis/Autophagy/Entotic.cell.death |
| autophagy related 7 | ATG7 | ENSG00000197548 | Ferroptosis/Autophagy/Entotic.cell.death |
| ceruloplasmin | CP | ENSG00000047457 | Ferroptosis/Autophagy/Lysosome.dependent.cell.death |
| microtubule associated protein 1 light chain 3 alpha | MAP1LC3A | ENSG00000101460 | Ferroptosis/Autophagy/Lysosome.dependent.cell.death |
| nuclear receptor coactivator 4 | NCOA4 | ENSG00000266412 | Ferroptosis/Autophagy/Lysosome.dependent.cell.death |
| ferritin light chain | FTL | ENSG00000087086 | Ferroptosis/Autophagy/Necroptosis/Lysosome.dependent.cell.death |
| ferritin heavy chain 1 | FTH1 | ENSG00000167996 | Ferroptosis/Autophagy/Necroptosis/Lysosome.dependent.cell.death |
| kelch like ECH associated protein 1 | KEAP1 | ENSG00000079999 | Ferroptosis/Autophagy/Oxeiptosis |
| glutaminase | GLS | ENSG00000115419 | Ferroptosis/Cuproptosis |
| cytochrome b-245 beta chain | CYBB | ENSG00000165168 | Ferroptosis/Entotic.cell.death |
| solute carrier family 11 member 2 | SLC11A2 | ENSG00000110911 | Ferroptosis/Lysosome.dependent.cell.death |
| voltage dependent anion channel 3 | VDAC3 | ENSG00000078668 | Ferroptosis/Necroptosis |
| arachidonate 15-lipoxygenase | ALOX15 | ENSG00000161905 | Ferroptosis/Necroptosis |
| vesicle associated membrane protein 7 | VAMP7 | ENSG00000292366 | Lysosome.dependent.cell.death |
| BLOC-1 related complex subunit 5 | BORCS5 | ENSG00000280689 | Lysosome.dependent.cell.death |
| alpha glucosidase | GAA | ENSG00000291507 | Lysosome.dependent.cell.death |
| WASP family homolog 3 / pseudogene | WASH3P | ENSG00000288207 | Lysosome.dependent.cell.death |
| phosphatidylinositol-4 /5-bisphosphate 4-phosphatase 1 | PIP4P1 | ENSG00000291802 | Lysosome.dependent.cell.death |
| rubicon like autophagy enhancer | RUBCNL | ENSG00000102445 | Lysosome.dependent.cell.death |
| cathepsin B | CTSB | ENSG00000285132 | Lysosome.dependent.cell.death |
| CD300a molecule | CD300A | ENSG00000291918 | Lysosome.dependent.cell.death |
| BLK proto-oncogene / Src family tyrosine kinase | BLK | ENSG00000285369 | Lysosome.dependent.cell.death |
| prostaglandin D2 receptor | PTGDR | ENSG00000168229 | Lysosome.dependent.cell.death |
| CLN5 intracellular trafficking protein | CLN5 | ENSG00000102805 | Lysosome.dependent.cell.death |
| ectonucleoside triphosphate diphosphohydrolase 4 | ENTPD4 | ENSG00000197217 | Lysosome.dependent.cell.death |
| chromogranin A | CHGA | ENSG00000276781 | Lysosome.dependent.cell.death |
| LYN proto-oncogene / Src family tyrosine kinase | LYN | ENSG00000254087 | Lysosome.dependent.cell.death |
| cathepsin Z | CTSZ | ENSG00000101160 | Lysosome.dependent.cell.death |
| pleckstrin homology and RUN domain containing M1 | PLEKHM1 | ENSG00000277111 | Lysosome.dependent.cell.death |
| galactosylceramidase | GALC | ENSG00000054983 | Lysosome.dependent.cell.death |
| legumain | LGMN | ENSG00000100600 | Lysosome.dependent.cell.death |
| pleckstrin homology and RUN domain containing M1 | PLEKHM1 | ENSG00000276358 | Lysosome.dependent.cell.death |
| chromosome 12 open reading frame 4 | C12orf4 | ENSG00000047621 | Lysosome.dependent.cell.death |
| adaptor related protein complex 4 subunit epsilon 1 | AP4E1 | ENSG00000081014 | Lysosome.dependent.cell.death |
| iduronate 2-sulfatase | IDS | ENSG00000010404 | Lysosome.dependent.cell.death |
| spleen associated tyrosine kinase | SYK | ENSG00000165025 | Lysosome.dependent.cell.death |
| arylsulfatase B | ARSB | ENSG00000113273 | Lysosome.dependent.cell.death |
| lysosomal protein transmembrane 4 beta | LAPTM4B | ENSG00000104341 | Lysosome.dependent.cell.death |
| interleukin 13 receptor subunit alpha 2 | IL13RA2 | ENSG00000123496 | Lysosome.dependent.cell.death |
| BLOC-1 related complex subunit 5 | BORCS5 | ENSG00000165714 | Lysosome.dependent.cell.death |
| alpha-N-acetylgalactosaminidase | NAGA | ENSG00000198951 | Lysosome.dependent.cell.death |
| chromogranin A | CHGA | ENSG00000100604 | Lysosome.dependent.cell.death |
| vesicle associated membrane protein 7 | VAMP7 | ENSG00000124333 | Lysosome.dependent.cell.death |
| NPC intracellular cholesterol transporter 2 | NPC2 | ENSG00000119655 | Lysosome.dependent.cell.death |
| forkhead box F1 | FOXF1 | ENSG00000103241 | Lysosome.dependent.cell.death |
| adhesion G protein-coupled receptor E2 | ADGRE2 | ENSG00000127507 | Lysosome.dependent.cell.death |
| adaptor related protein complex 3 subunit sigma 1 | AP3S1 | ENSG00000177879 | Lysosome.dependent.cell.death |
| sulfatase modifying factor 1 | SUMF1 | ENSG00000144455 | Lysosome.dependent.cell.death |
| cathepsin O | CTSO | ENSG00000263238 | Lysosome.dependent.cell.death |
| glucuronidase beta | GUSB | ENSG00000169919 | Lysosome.dependent.cell.death |
| adaptor related protein complex 1 subunit beta 1 | AP1B1 | ENSG00000100280 | Lysosome.dependent.cell.death |
| hyaluronidase 1 | HYAL1 | ENSG00000114378 | Lysosome.dependent.cell.death |
| prostaglandin D2 synthase | PTGDS | ENSG00000107317 | Lysosome.dependent.cell.death |
| palmitoyl-protein thioesterase 2 | PPT2 | ENSG00000206256 | Lysosome.dependent.cell.death |
| palmitoyl-protein thioesterase 2 | PPT2 | ENSG00000168452 | Lysosome.dependent.cell.death |
| N-acetylglucosamine-1-phosphodiester alpha-N-acetylglucosaminidase | NAGPA | ENSG00000103174 | Lysosome.dependent.cell.death |
| adaptor related protein complex 1 subunit sigma 2 | AP1S2 | ENSG00000182287 | Lysosome.dependent.cell.death |
| neuraminidase 1 | NEU1 | ENSG00000227315 | Lysosome.dependent.cell.death |
| solute carrier family 17 member 5 | SLC17A5 | ENSG00000119899 | Lysosome.dependent.cell.death |
| neuraminidase 1 | NEU1 | ENSG00000234343 | Lysosome.dependent.cell.death |
| arylsulfatase A | ARSA | ENSG00000100299 | Lysosome.dependent.cell.death |
| neuraminidase 1 | NEU1 | ENSG00000223957 | Lysosome.dependent.cell.death |
| palmitoyl-protein thioesterase 2 | PPT2 | ENSG00000228116 | Lysosome.dependent.cell.death |
| neuraminidase 1 | NEU1 | ENSG00000228691 | Lysosome.dependent.cell.death |
| myosin heavy chain 9 | MYH9 | ENSG00000100345 | Lysosome.dependent.cell.death |
| N-acylsphingosine amidohydrolase 1 | ASAH1 | ENSG00000104763 | Lysosome.dependent.cell.death |
| palmitoyl-protein thioesterase 2 | PPT2 | ENSG00000236649 | Lysosome.dependent.cell.death |
| adaptor related protein complex 1 subunit sigma 1 | AP1S1 | ENSG00000106367 | Lysosome.dependent.cell.death |
| palmitoyl-protein thioesterase 2 | PPT2 | ENSG00000231618 | Lysosome.dependent.cell.death |
| neuraminidase 1 | NEU1 | ENSG00000184494 | Lysosome.dependent.cell.death |
| N-acetylglucosamine-1-phosphate transferase subunits alpha and beta | GNPTAB | ENSG00000111670 | Lysosome.dependent.cell.death |
| neuraminidase 1 | NEU1 | ENSG00000227129 | Lysosome.dependent.cell.death |
| lysosomal protein transmembrane 5 | LAPTM5 | ENSG00000162511 | Lysosome.dependent.cell.death |
| phosphatidylinositol-4 /5-bisphosphate 4-phosphatase 1 | PIP4P1 | ENSG00000165782 | Lysosome.dependent.cell.death |
| palmitoyl-protein thioesterase 2 | PPT2 | ENSG00000206329 | Lysosome.dependent.cell.death |
| sortilin related receptor 1 | SORL1 | ENSG00000137642 | Lysosome.dependent.cell.death |
| palmitoyl-protein thioesterase 2 | PPT2 | ENSG00000227600 | Lysosome.dependent.cell.death |
| ATPase phospholipid transporting 10B (putative) | ATP10B | ENSG00000118322 | Lysosome.dependent.cell.death |
| neuraminidase 1 | NEU1 | ENSG00000234846 | Lysosome.dependent.cell.death |
| sorting nexin 4 | SNX4 | ENSG00000114520 | Lysosome.dependent.cell.death |
| heparan-alpha-glucosaminide N-acetyltransferase | HGSNAT | ENSG00000165102 | Lysosome.dependent.cell.death |
| cathepsin G | CTSG | ENSG00000100448 | Lysosome.dependent.cell.death |
| zinc finger FYVE-type containing 16 | ZFYVE16 | ENSG00000039319 | Lysosome.dependent.cell.death |
| cathepsin L | CTSL | ENSG00000135047 | Lysosome.dependent.cell.death |
| ATPase H+ transporting accessory protein 1 | ATP6AP1 | ENSG00000071553 | Lysosome.dependent.cell.death |
| HPS6 biogenesis of lysosomal organelles complex 2 subunit 3 | HPS6 | ENSG00000166189 | Lysosome.dependent.cell.death |
| phospholipase A2 group XV | PLA2G15 | ENSG00000103066 | Lysosome.dependent.cell.death |
| ATPase H+ transporting V0 subunit a4 | ATP6V0A4 | ENSG00000105929 | Lysosome.dependent.cell.death |
| CD68 molecule | CD68 | ENSG00000129226 | Lysosome.dependent.cell.death |
| adaptor related protein complex 3 subunit mu 1 | AP3M1 | ENSG00000185009 | Lysosome.dependent.cell.death |
| lysosomal protein transmembrane 4 alpha | LAPTM4A | ENSG00000068697 | Lysosome.dependent.cell.death |
| golgi associated / gamma adaptin ear containing / ARF binding protein 1 | GGA1 | ENSG00000100083 | Lysosome.dependent.cell.death |
| adaptor related protein complex 3 subunit sigma 2 | AP3S2 | ENSG00000157823 | Lysosome.dependent.cell.death |
| cathepsin V | CTSV | ENSG00000136943 | Lysosome.dependent.cell.death |
| Rac family small GTPase 2 | RAC2 | ENSG00000128340 | Lysosome.dependent.cell.death |
| golgi associated / gamma adaptin ear containing / ARF binding protein 2 | GGA2 | ENSG00000103365 | Lysosome.dependent.cell.death |
| lipase A / lysosomal acid type | LIPA | ENSG00000107798 | Lysosome.dependent.cell.death |
| insulin like growth factor 2 receptor | IGF2R | ENSG00000197081 | Lysosome.dependent.cell.death |
| adaptor related protein complex 4 subunit mu 1 | AP4M1 | ENSG00000221838 | Lysosome.dependent.cell.death |
| neuraminidase 1 | NEU1 | ENSG00000204386 | Lysosome.dependent.cell.death |
| BLOC-1 related complex subunit 6 | BORCS6 | ENSG00000196544 | Lysosome.dependent.cell.death |
| cathepsin H | CTSH | ENSG00000103811 | Lysosome.dependent.cell.death |
| clathrin heavy chain like 1 | CLTCL1 | ENSG00000070371 | Lysosome.dependent.cell.death |
| adaptor related protein complex 4 subunit sigma 1 | AP4S1 | ENSG00000100478 | Lysosome.dependent.cell.death |
| phospholipase A2 group III | PLA2G3 | ENSG00000100078 | Lysosome.dependent.cell.death |
| FES proto-oncogene / tyrosine kinase | FES | ENSG00000182511 | Lysosome.dependent.cell.death |
| mannose-6-phosphate receptor / cation dependent | M6PR | ENSG00000003056 | Lysosome.dependent.cell.death |
| adaptor related protein complex 3 subunit beta 2 | AP3B2 | ENSG00000103723 | Lysosome.dependent.cell.death |
| RAB3A / member RAS oncogene family | RAB3A | ENSG00000105649 | Lysosome.dependent.cell.death |
| VPS33A core subunit of CORVET and HOPS complexes | VPS33A | ENSG00000139719 | Lysosome.dependent.cell.death |
| adaptor related protein complex 1 subunit mu 1 | AP1M1 | ENSG00000072958 | Lysosome.dependent.cell.death |
| vacuolar protein sorting 4 homolog A | VPS4A | ENSG00000132612 | Lysosome.dependent.cell.death |
| palmitoyl-protein thioesterase 2 | PPT2 | ENSG00000221988 | Lysosome.dependent.cell.death |
| sphingosine kinase 2 | SPHK2 | ENSG00000063176 | Lysosome.dependent.cell.death |
| synaptotagmin like 4 | SYTL4 | ENSG00000102362 | Lysosome.dependent.cell.death |
| sorting nexin 16 | SNX16 | ENSG00000104497 | Lysosome.dependent.cell.death |
| hexosaminidase subunit alpha | HEXA | ENSG00000213614 | Lysosome.dependent.cell.death |
| nudE neurodevelopment protein 1 like 1 | NDEL1 | ENSG00000166579 | Lysosome.dependent.cell.death |
| cathepsin O | CTSO | ENSG00000256043 | Lysosome.dependent.cell.death |
| ATP binding cassette subfamily A member 2 | ABCA2 | ENSG00000107331 | Lysosome.dependent.cell.death |
| RAB34 / member RAS oncogene family | RAB34 | ENSG00000109113 | Lysosome.dependent.cell.death |
| mucolipin TRP cation channel 1 | MCOLN1 | ENSG00000090674 | Lysosome.dependent.cell.death |
| linker for activation of T cells family member 2 | LAT2 | ENSG00000086730 | Lysosome.dependent.cell.death |
| nuclear receptor subfamily 4 group A member 3 | NR4A3 | ENSG00000119508 | Lysosome.dependent.cell.death |
| mannosidase alpha class 2B member 1 | MAN2B1 | ENSG00000104774 | Lysosome.dependent.cell.death |
| differentially expressed in FDCP 8 homolog | DEF8 | ENSG00000140995 | Lysosome.dependent.cell.death |
| cathepsin D | CTSD | ENSG00000117984 | Lysosome.dependent.cell.death |
| mannosidase beta | MANBA | ENSG00000109323 | Lysosome.dependent.cell.death |
| mast cell immunoglobulin like receptor 1 | MILR1 | ENSG00000271605 | Lysosome.dependent.cell.death |
| ADP ribosylation factor like GTPase 8B | ARL8B | ENSG00000134108 | Lysosome.dependent.cell.death |
| hepatocyte growth factor-regulated tyrosine kinase substrate | HGS | ENSG00000185359 | Lysosome.dependent.cell.death |
| cathepsin F | CTSF | ENSG00000174080 | Lysosome.dependent.cell.death |
| golgi associated / gamma adaptin ear containing / ARF binding protein 3 | GGA3 | ENSG00000125447 | Lysosome.dependent.cell.death |
| adaptor related protein complex 1 subunit mu 2 | AP1M2 | ENSG00000129354 | Lysosome.dependent.cell.death |
| N-acetylglucosamine-1-phosphate transferase subunit gamma | GNPTG | ENSG00000090581 | Lysosome.dependent.cell.death |
| N-acetyl-alpha-glucosaminidase | NAGLU | ENSG00000108784 | Lysosome.dependent.cell.death |
| cathepsin W | CTSW | ENSG00000172543 | Lysosome.dependent.cell.death |
| adaptor related protein complex 1 subunit gamma 1 | AP1G1 | ENSG00000166747 | Lysosome.dependent.cell.death |
| cytokine dependent hematopoietic cell linker | CLNK | ENSG00000109684 | Lysosome.dependent.cell.death |
| interleukin 13 | IL13 | ENSG00000169194 | Lysosome.dependent.cell.death |
| FER tyrosine kinase | FER | ENSG00000151422 | Lysosome.dependent.cell.death |
| VPS33B late endosome and lysosome associated | VPS33B | ENSG00000184056 | Lysosome.dependent.cell.death |
| CD63 molecule | CD63 | ENSG00000135404 | Lysosome.dependent.cell.death |
| scavenger receptor class B member 2 | SCARB2 | ENSG00000138760 | Lysosome.dependent.cell.death |
| synaptosome associated protein 23 | SNAP23 | ENSG00000092531 | Lysosome.dependent.cell.death |
| adaptor related protein complex 3 subunit beta 1 | AP3B1 | ENSG00000132842 | Lysosome.dependent.cell.death |
| arylsulfatase G | ARSG | ENSG00000141337 | Lysosome.dependent.cell.death |
| microtubule associated protein 6 | MAP6 | ENSG00000171533 | Lysosome.dependent.cell.death |
| WASP family homolog 3 / pseudogene | WASH3P | ENSG00000185596 | Lysosome.dependent.cell.death |
| alpha-L-iduronidase | IDUA | ENSG00000127415 | Lysosome.dependent.cell.death |
| syntaxin binding protein 1 | STXBP1 | ENSG00000136854 | Lysosome.dependent.cell.death |
| galactosidase alpha | GLA | ENSG00000102393 | Lysosome.dependent.cell.death |
| clathrin light chain B | CLTB | ENSG00000175416 | Lysosome.dependent.cell.death |
| tripeptidyl peptidase 1 | TPP1 | ENSG00000166340 | Lysosome.dependent.cell.death |
| KxDL motif containing 1 | KXD1 | ENSG00000105700 | Lysosome.dependent.cell.death |
| complexin 2 | CPLX2 | ENSG00000145920 | Lysosome.dependent.cell.death |
| clathrin light chain A | CLTA | ENSG00000122705 | Lysosome.dependent.cell.death |
| ganglioside GM2 activator | GM2A | ENSG00000196743 | Lysosome.dependent.cell.death |
| glucosamine (N-acetyl)-6-sulfatase | GNS | ENSG00000135677 | Lysosome.dependent.cell.death |
| CD164 molecule | CD164 | ENSG00000135535 | Lysosome.dependent.cell.death |
| adaptor related protein complex 3 subunit mu 2 | AP3M2 | ENSG00000070718 | Lysosome.dependent.cell.death |
| BLK proto-oncogene / Src family tyrosine kinase | BLK | ENSG00000136573 | Lysosome.dependent.cell.death |
| galactosamine (N-acetyl)-6-sulfatase | GALNS | ENSG00000141012 | Lysosome.dependent.cell.death |
| cathepsin B | CTSB | ENSG00000164733 | Lysosome.dependent.cell.death |
| interleukin 4 receptor | IL4R | ENSG00000077238 | Lysosome.dependent.cell.death |
| CD300a molecule | CD300A | ENSG00000167851 | Lysosome.dependent.cell.death |
| palmitoyl-protein thioesterase 1 | PPT1 | ENSG00000131238 | Lysosome.dependent.cell.death |
| phosphatidylinositol-4 /5-bisphosphate 3-kinase catalytic subunit gamma | PIK3CG | ENSG00000105851 | Lysosome.dependent.cell.death |
| sperm associated antigen 9 | SPAG9 | ENSG00000008294 | Lysosome.dependent.cell.death |
| prosaposin like 1 | PSAPL1 | ENSG00000178597 | Lysosome.dependent.cell.death |
| cathepsin E | CTSE | ENSG00000196188 | Lysosome.dependent.cell.death |
| acid phosphatase 5 / tartrate resistant | ACP5 | ENSG00000102575 | Lysosome.dependent.cell.death |
| transmembrane protein 106B | TMEM106B | ENSG00000106460 | Lysosome.dependent.cell.death |
| MAS related GPR family member X2 | MRGPRX2 | ENSG00000183695 | Lysosome.dependent.cell.death |
| hexosaminidase subunit beta | HEXB | ENSG00000049860 | Lysosome.dependent.cell.death |
| syntaxin binding protein 2 | STXBP2 | ENSG00000076944 | Lysosome.dependent.cell.death |
| ATP binding cassette subfamily B member 9 | ABCB9 | ENSG00000150967 | Lysosome.dependent.cell.death |
| galectin 9 | LGALS9 | ENSG00000168961 | Lysosome.dependent.cell.death |
| deoxyribonuclease 2 / lysosomal | DNASE2 | ENSG00000105612 | Lysosome.dependent.cell.death |
| cathepsin K | CTSK | ENSG00000143387 | Lysosome.dependent.cell.death |
| cathepsin S | CTSS | ENSG00000163131 | Lysosome.dependent.cell.death |
| cystinosin / lysosomal cystine transporter | CTNS | ENSG00000040531 | Lysosome.dependent.cell.death |
| FGR proto-oncogene / Src family tyrosine kinase | FGR | ENSG00000000938 | Lysosome.dependent.cell.death |
| biogenesis of lysosomal organelles complex 1 subunit 1 | BLOC1S1 | ENSG00000135441 | Lysosome.dependent.cell.death |
| adaptor related protein complex 3 subunit delta 1 | AP3D1 | ENSG00000065000 | Lysosome.dependent.cell.death |
| unc-13 homolog D | UNC13D | ENSG00000092929 | Lysosome.dependent.cell.death |
| GRB2 associated binding protein 2 | GAB2 | ENSG00000033327 | Lysosome.dependent.cell.death |
| pleckstrin homology and RUN domain containing M1 | PLEKHM1 | ENSG00000225190 | Lysosome.dependent.cell.death |
| T cell immune regulator 1 / ATPase H+ transporting V0 subunit a3 | TCIRG1 | ENSG00000110719 | Lysosome.dependent.cell.death |
| napsin A aspartic peptidase | NAPSA | ENSG00000131400 | Lysosome.dependent.cell.death |
| deoxyribonuclease 2 beta | DNASE2B | ENSG00000137976 | Lysosome.dependent.cell.death |
| acid phosphatase 2 / lysosomal | ACP2 | ENSG00000134575 | Lysosome.dependent.cell.death |
| Cbl proto-oncogene | CBL | ENSG00000110395 | Lysosome.dependent.cell.death |
| clathrin heavy chain | CLTC | ENSG00000141367 | Lysosome.dependent.cell.death |
| N-sulfoglucosamine sulfohydrolase | SGSH | ENSG00000181523 | Lysosome.dependent.cell.death |
| solute carrier family 11 member 1 | SLC11A1 | ENSG00000018280 | Lysosome.dependent.cell.death |
| vesicle associated membrane protein 8 | VAMP8 | ENSG00000118640 | Lysosome.dependent.cell.death |
| family with sequence similarity 98 member A | FAM98A | ENSG00000119812 | Lysosome.dependent.cell.death |
| S100 calcium binding protein A13 | S100A13 | ENSG00000189171 | Lysosome.dependent.cell.death |
| SNAP associated protein | SNAPIN | ENSG00000143553 | Lysosome.dependent.cell.death |
| GRIP and coiled-coil domain containing 2 | GCC2 | ENSG00000135968 | Lysosome.dependent.cell.death |
| alpha glucosidase | GAA | ENSG00000171298 | Lysosome.dependent.cell.death |
| alpha-L-fucosidase 1 | FUCA1 | ENSG00000179163 | Lysosome.dependent.cell.death |
| adaptor related protein complex 1 subunit sigma 3 | AP1S3 | ENSG00000152056 | Lysosome.dependent.cell.death |
| GATA binding protein 2 | GATA2 | ENSG00000179348 | Lysosome.dependent.cell.death |
| CD84 molecule | CD84 | ENSG00000066294 | Lysosome.dependent.cell.death |
| kinesin family member 1B | KIF1B | ENSG00000054523 | Lysosome.dependent.cell.death |
| adaptor related protein complex 4 subunit beta 1 | AP4B1 | ENSG00000134262 | Lysosome.dependent.cell.death |
| phosphatidylinositol-4 /5-bisphosphate 3-kinase catalytic subunit delta | PIK3CD | ENSG00000171608 | Lysosome.dependent.cell.death |
| ADP ribosylation factor 1 | ARF1 | ENSG00000143761 | Lysosome.dependent.cell.death |
| pleckstrin homology and RUN domain containing M2 | PLEKHM2 | ENSG00000116786 | Lysosome.dependent.cell.death |
| ring finger protein 31 | RNF31 | ENSG00000285152 | Necroptosis |
| interferon regulatory factor 9 | IRF9 | ENSG00000285048 | Necroptosis |
| spermatogenesis associated 2 | SPATA2 | ENSG00000158480 | Necroptosis |
| glutamate dehydrogenase 2 | GLUD2 | ENSG00000288118 | Necroptosis |
| interferon gamma receptor 2 | IFNGR2 | ENSG00000159128 | Necroptosis |
| interferon alpha and beta receptor subunit 2 | IFNAR2 | ENSG00000159110 | Necroptosis |
| interferon alpha and beta receptor subunit 1 | IFNAR1 | ENSG00000142166 | Necroptosis |
| glutamate dehydrogenase 2 | GLUD2 | ENSG00000182890 | Necroptosis |
| mixed lineage kinase domain like pseudokinase | MLKL | ENSG00000168404 | Necroptosis |
| interferon gamma receptor 2 | IFNGR2 | ENSG00000262795 | Necroptosis |
| transient receptor potential cation channel subfamily M member 7 | TRPM7 | ENSG00000092439 | Necroptosis |
| Z-DNA binding protein 1 | ZBP1 | ENSG00000124256 | Necroptosis |
| SHANK associated RH domain interactor | SHARPIN | ENSG00000179526 | Necroptosis |
| glutamate dehydrogenase 1 | GLUD1 | ENSG00000148672 | Necroptosis |
| X-linked inhibitor of apoptosis | XIAP | ENSG00000101966 | Necroptosis |
| peptidylprolyl isomerase D | PPID | ENSG00000171497 | Necroptosis |
| signal transducer and activator of transcription 6 | STAT6 | ENSG00000166888 | Necroptosis |
| signal transducer and activator of transcription 4 | STAT4 | ENSG00000138378 | Necroptosis |
| interferon regulatory factor 9 | IRF9 | ENSG00000213928 | Necroptosis |
| ring finger protein 31 | RNF31 | ENSG00000092098 | Necroptosis |
| signal transducer and activator of transcription 5A | STAT5A | ENSG00000126561 | Necroptosis |
| interferon gamma receptor 1 | IFNGR1 | ENSG00000027697 | Necroptosis |
| charged multivesicular body protein 5 | CHMP5 | ENSG00000086065 | Necroptosis |
| eukaryotic translation initiation factor 2 alpha kinase 2 | EIF2AK2 | ENSG00000055332 | Necroptosis |
| Janus kinase 3 | JAK3 | ENSG00000105639 | Necroptosis |
| tyrosine kinase 2 | TYK2 | ENSG00000105397 | Necroptosis |
| TNF receptor associated factor 5 | TRAF5 | ENSG00000082512 | Necroptosis |
| signal transducer and activator of transcription 5B | STAT5B | ENSG00000173757 | Necroptosis |
| signal transducer and activator of transcription 2 | STAT2 | ENSG00000170581 | Necroptosis |
| baculoviral IAP repeat containing 3 | BIRC3 | ENSG00000023445 | Necroptosis |
| baculoviral IAP repeat containing 2 | BIRC2 | ENSG00000110330 | Necroptosis |
| signal transducer and activator of transcription 1 | STAT1 | ENSG00000115415 | Necroptosis |
| ubiquitin specific peptidase 21 | USP21 | ENSG00000143258 | Necroptosis |
| calpain 2 | CAPN2 | ENSG00000162909 | Necroptosis |
| Janus kinase 1 | JAK1 | ENSG00000162434 | Necroptosis |
| sphingomyelin phosphodiesterase 1 | SMPD1 | ENSG00000166311 | Necroptosis/Lysosome.dependent.cell.death |
| PGAM family member 5 / mitochondrial serine/threonine protein phosphatase | PGAM5 | ENSG00000247077 | Necroptosis/Oxeiptosis |
| peptidyl arginine deiminase 4 | PADI4 | ENSG00000280908 | Netotic.cell.death |
| peptidyl arginine deiminase 4 | PADI4 | ENSG00000159339 | Netotic.cell.death |
| matrix metallopeptidase 1 | MMP1 | ENSG00000196611 | Netotic.cell.death |
| cathelicidin antimicrobial peptide | CAMP | ENSG00000164047 | Netotic.cell.death |
| MIA SH3 domain containing | MIA | ENSG00000261857 | Netotic.cell.death |
| myeloperoxidase | MPO | ENSG00000005381 | Netotic.cell.death |
| autoimmune regulator | AIRE | ENSG00000160224 | Oxeiptosis |
| 8-oxoguanine DNA glycosylase | OGG1 | ENSG00000114026 | Parthanatos |
| ring finger protein 146 | RNF146 | ENSG00000118518 | Parthanatos/Entotic.cell.death |
| NLR family pyrin domain containing 2 | NLRP2 | ENSG00000275082 | Pyroptosis |
| NLR family pyrin domain containing 7 | NLRP7 | ENSG00000277786 | Pyroptosis |
| NLR family pyrin domain containing 7 | NLRP7 | ENSG00000277071 | Pyroptosis |
| NLR family pyrin domain containing 2 | NLRP2 | ENSG00000278789 | Pyroptosis |
| NLR family pyrin domain containing 7 | NLRP7 | ENSG00000275483 | Pyroptosis |
| NLR family pyrin domain containing 2 | NLRP2 | ENSG00000278682 | Pyroptosis |
| NLR family pyrin domain containing 2 | NLRP2 | ENSG00000277060 | Pyroptosis |
| NLR family pyrin domain containing 7 | NLRP7 | ENSG00000278173 | Pyroptosis |
| NLR family pyrin domain containing 7 | NLRP7 | ENSG00000274174 | Pyroptosis |
| NLR family pyrin domain containing 2 | NLRP2 | ENSG00000275796 | Pyroptosis |
| NLR family pyrin domain containing 2 | NLRP2 | ENSG00000274638 | Pyroptosis |
| NLR family pyrin domain containing 7 | NLRP7 | ENSG00000277179 | Pyroptosis |
| NLR family pyrin domain containing 7 | NLRP7 | ENSG00000276804 | Pyroptosis |
| NLR family pyrin domain containing 2 | NLRP2 | ENSG00000275843 | Pyroptosis |
| NLR family pyrin domain containing 2 | NLRP2 | ENSG00000275399 | Pyroptosis |
| NLR family pyrin domain containing 7 | NLRP7 | ENSG00000277776 | Pyroptosis |
| NLR family pyrin domain containing 7 | NLRP7 | ENSG00000274571 | Pyroptosis |
| NLR family pyrin domain containing 2 | NLRP2 | ENSG00000273992 | Pyroptosis |
| phospholipase C gamma 1 | PLCG1 | ENSG00000124181 | Pyroptosis |
| cytochrome c / somatic | CYCS | ENSG00000172115 | Pyroptosis |
| gasdermin A | GSDMA | ENSG00000167914 | Pyroptosis |
| gasdermin C | GSDMC | ENSG00000285114 | Pyroptosis |
| gasdermin D | GSDMD | ENSG00000278718 | Pyroptosis |
| gasdermin C | GSDMC | ENSG00000147697 | Pyroptosis |
| SR-related CTD associated factor 11 | SCAF11 | ENSG00000139218 | Pyroptosis |
| interleukin 18 | IL18 | ENSG00000150782 | Pyroptosis |
| gasdermin D | GSDMD | ENSG00000104518 | Pyroptosis |
| caspase 6 | CASP6 | ENSG00000138794 | Pyroptosis |
| interferon regulatory factor 1 | IRF1 | ENSG00000125347 | Pyroptosis |
| interferon regulatory factor 2 | IRF2 | ENSG00000168310 | Pyroptosis |
| gasdermin B | GSDMB | ENSG00000073605 | Pyroptosis |
| NLR family pyrin domain containing 2 | NLRP2 | ENSG00000022556 | Pyroptosis |
| NLR family pyrin domain containing 1 | NLRP1 | ENSG00000091592 | Pyroptosis |
| NLR family pyrin domain containing 7 | NLRP7 | ENSG00000167634 | Pyroptosis |
| TIR domain containing adaptor protein | TIRAP | ENSG00000150455 | Pyroptosis |
| NLR family CARD domain containing 4 | NLRC4 | ENSG00000091106 | Pyroptosis |
| charged multivesicular body protein 3 | CHMP3 | ENSG00000115561 | Pyroptosis |
| nucleotide binding oligomerization domain containing 1 | NOD1 | ENSG00000106100 | Pyroptosis/Autophagy |
| protein kinase cAMP-activated catalytic subunit alpha | PRKACA | ENSG00000288516 | Pyroptosis/Autophagy |
| NLR family pyrin domain containing 6 | NLRP6 | ENSG00000174885 | Pyroptosis/Autophagy |
| pejvakin | PJVK | ENSG00000204311 | Pyroptosis/Autophagy |
| nucleotide binding oligomerization domain containing 2 | NOD2 | ENSG00000167207 | Pyroptosis/Autophagy |
| protein kinase cAMP-activated catalytic subunit alpha | PRKACA | ENSG00000072062 | Pyroptosis/Autophagy |
| charged multivesicular body protein 4A | CHMP4A | ENSG00000285302 | Pyroptosis/Autophagy/Necroptosis |
| high mobility group box 1 | HMGB1 | ENSG00000189403 | Pyroptosis/Autophagy/Necroptosis |
| charged multivesicular body protein 4B | CHMP4B | ENSG00000101421 | Pyroptosis/Autophagy/Necroptosis |
| charged multivesicular body protein 4A | CHMP4A | ENSG00000254505 | Pyroptosis/Autophagy/Necroptosis |
| glutathione peroxidase 4 | GPX4 | ENSG00000167468 | Pyroptosis/Ferroptosis |
| charged multivesicular body protein 7 | CHMP7 | ENSG00000147457 | Pyroptosis/Necroptosis |
| charged multivesicular body protein 4C | CHMP4C | ENSG00000164695 | Pyroptosis/Necroptosis |
| charged multivesicular body protein 2B | CHMP2B | ENSG00000083937 | Pyroptosis/Necroptosis |
| charged multivesicular body protein 6 | CHMP6 | ENSG00000176108 | Pyroptosis/Necroptosis |
| charged multivesicular body protein 2A | CHMP2A | ENSG00000130724 | Pyroptosis/Necroptosis |
| NLR family pyrin domain containing 3 | NLRP3 | ENSG00000162711 | Pyroptosis/Necroptosis |
| elastase / neutrophil expressed | ELANE | ENSG00000277571 | Pyroptosis/Netotic.cell.death |
| elastase / neutrophil expressed | ELANE | ENSG00000197561 | Pyroptosis/Netotic.cell.death |

Table S2. Data summary. A list of SOC cohorts used in this study is shown.

| Cancer Type | Tissue | Sequencing type | Number of patients | Data accession number | PMID |
| --- | --- | --- | --- | --- | --- |
| SOC | non-cancer/tumor | bulk RNA-seq | 338 | - | 28398314 |
| SOC | tumor | bulk RNA-seq | 340 | - | 21720365 |
| SOC | tumor | Array | 79 | GSE26193 | 22101765 |
| SOC | tumor | Array | 70 | GSE63885 | 24478986 |
| SOC | tumor | Array | 276 | GSE140082 | 28159814 |
| SOC | tumor | Array | 174 | GSE53963 | 25269487 |
| SOC | tumor | scRNA-seq | 1 | GSE213243 | 36248860 |
| SOC | tumor | scRNA-seq | 7 | GSE184880 | 35675036 |
| SOC | tumor | ST-seq (Visium) | 1 | - | - |

Table S3. ROC values of biomarkers in OC.

| biomarkers | ROC | | References |
| --- | --- | --- | --- |
|  | 1-5 year | 6-9 year |  |
| This study | 0.52-0.82 | 0.68-0.92 | - |
| protein risk scores | 0.596-0.749 | - | (Jiang et al. 2023) |
| metabolism-related gene prognostic index (MRGPI) | 0.621-0.759 | 0.755-0.759 | (Guo et al. 2024) |
| genes associated with copper metabolism (CMRGs) | 0.394-0.740 | - | (Zhao et al. 2023) |
| RNA-modification regulatory genes (RRGs) | 0.835-0.872 | - | (Zheng, Li and Zhan 2022) |
| RNA Adenosine Modification-Related Subtypes（RMW score） | 0.661- 0.761 | - | (Ni et al. 2022) |
| m5C RNA Modification-Related Genes | 0.635-0.721 | - | (Liu et al. 2023b) |
| m5c-related lncRNAs | 0.677-0.715 | - | (Wang et al. 2023a) |
| N6-methyladenosine (m6A) modification and lncRNAs | 0.567-0.662 | - | (Zheng et al. 2021) |
| 7 immune-related prognostic lncRNAs | 0.72 | - | (Feng et al. 2023) |
| immune-related lncRNAs | 0.604-0.613 | - | (Li et al. 2022) |
| ferroptosis-related lncRNA | 0.762-0.793 | - | (Yang et al. 2023) |
| ferroptosis-related lncRNA | 0.6873-0.7354 | - | (Wang et al. 2022b) |
| cuproptosis-related lncRNAs | 0.577-0.750 | - | (Liu et al. 2023a) |
| mitophagy-related lncRNAs | 0.524-0.700 | - | (Wang et al. 2023b) |
| exosome-derived lncRNAs | 0.536-0.872 | - | (Cui et al. 2024) |
| necroptosis-related genes | 0.620-0.707 | - | (Wang et al. 2022c) |
| necroptosis-related genes | 0.596-0.720 | - | (Qin et al. 2024) |
| pyroptosis-related genes (PRGs) | 0.520-0.699 | - | (Zhang et al. 2023) |
| anoikis-related genes (ARGs) | 0.683-0.766 | - | (Duan and Xu 2023) |
| TMB-related risk model (TMBrisk) | 0.714-0.719 | - | (Wang et al. 2022a) |

Reference

Cui Y, et al. An exosome-derived lncRNA signature identified by machine learning associated with prognosis and biomarkers for immunotherapy in ovarian cancer. Frontiers in immunology. 2024;15:1228235.

Duan Y, Xu X. A signature based on anoikis-related genes for the evaluation of prognosis, immunoinfiltration, mutation, and therapeutic response in ovarian cancer. Frontiers in endocrinology. 2023;14:1193622.

Feng J, et al. Development and verification of a 7-lncRNA prognostic model based on tumor immunity for patients with ovarian cancer. Journal of ovarian research. 2023;16(1):31.

Guo S, et al. Metabolic-Related Gene Prognostic Index for Predicting Prognosis, Immunotherapy Response, and Candidate Drugs in Ovarian Cancer. Journal of chemical information and modeling. 2024;64(3):1066-80.

Jiang J, et al. Screening and Identification of a Prognostic Model of Ovarian Cancer by Combination of Transcriptomic and Proteomic Data. Biomolecules. 2023;13(4).

Li H, et al. Identification and validation of an immune-related lncRNAs signature to predict the overall survival of ovarian cancer. Frontiers in oncology. 2022;12:999654.

Liu L, et al. Developing four cuproptosis-related lncRNAs signature to predict prognosis and immune activity in ovarian cancer. Journal of ovarian research. 2023a;16(1):88.

Liu Y, et al. Contribution of m5C RNA Modification-Related Genes to Prognosis and Immunotherapy Prediction in Patients with Ovarian Cancer. Mediators of inflammation. 2023b;2023:1400267.

Ni X, et al. Crosstalk of RNA Adenosine Modification-Related Subtypes, Establishment of a Prognostic Model, and Immune Infiltration Characteristics in Ovarian Cancer. Frontiers in immunology. 2022;13:932876.

Qin Y, et al. Identification of necroptosis-related gene signatures for predicting the prognosis of ovarian cancer. Scientific reports. 2024;14(1):11133.

Wang C, et al. Identification and validation of m5c-related lncRNA risk model for ovarian cancer. Journal of ovarian research. 2023a;16(1):96.

Wang H, et al. A novel tumor mutational burden-based risk model predicts prognosis and correlates with immune infiltration in ovarian cancer. Frontiers in immunology. 2022a;13:943389.

Wang J, et al. Mitophagy-related long non-coding RNA signature predicts prognosis and drug response in Ovarian Cancer. Journal of ovarian research. 2023b;16(1):177.

Wang K, et al. Ferroptosis-Related Long Noncoding RNAs as Prognostic Biomarkers for Ovarian Cancer. Frontiers in oncology. 2022b;12:888699.

Wang Z, et al. Identification and Verification of Necroptosis-Related Gene Signature With Prognosis and Tumor Immune Microenvironment in Ovarian Cancer. Frontiers in immunology. 2022c;13:894718.

Yang S, et al. Construction of Ovarian Cancer Prognostic Model Based on the Investigation of Ferroptosis-Related lncRNA. Biomolecules. 2023;13(2).

Zhang B, et al. Exploration of pyroptosis-associated prognostic gene signature and lncRNA regulatory network in ovarian cancer. Computers in biology and medicine. 2023;164:107343.

Zhao S, et al. Identification of copper metabolism-related subtypes and establishment of the prognostic model in ovarian cancer. Frontiers in endocrinology. 2023;14:1145797.

Zheng J, et al. Identification and validation of lncRNAs involved in m6A regulation for patients with ovarian cancer. Cancer cell international. 2021;21(1):363.

Zheng P, Li N, Zhan X. Ovarian cancer subtypes based on the regulatory genes of RNA modifications: Novel prediction model of prognosis. Frontiers in endocrinology. 2022;13:972341.
